# Supplementary material for: Assessment of appropriateness of hospitalisations in Ukraine: analytical framework, method and findings
Source: BMJ Open. 2019 Dec 8;9(12):e030081. doi: 10.1136/bmjopen-2019-030081 (PMC6924815; doi:10.1136/bmjopen-2019-030081)
Supplement: Supplementary data [file bmjopen-2019-030081supp001.pdf]

Supplementary file 1

CRITERIA FOR APPROPRIATENESS OF HOSPITAL ADMISSIONS AND LENGTH OF INPATIENT STAY

Admission criteria

A. Severity of illness

1. Sudden loss of consciousness or disorientation (coma or unresponsiveness)
2. Acute confusional state, but not because of alcohol or narcotic intoxication
3. Acute loss of sight or hearing
4. Acute loss of ability to move part of the body
5. Acute ataxia (except for repeated paroxysms of acute ataxia in case of period/ episodic ataxias)
6. Persistent fever >38.0 C for 5 days without established diagnosis
7. Active bleeding which can lead to circulatory violations in case of absent homeostasis
8. Progressing acute neurological disorders
9. Acute or progressive sensory, motor, circulatory or respiratory disorder that can incapacitate the patient (inability to move, feed, breathe, urinate, etc.)
10. Unbearable pain (that cannot be managed by outpatient services (including primary or specialized outpatient or emergency service)
11. Acute abdominal pain with laboratory, ultrasound, X-ray disturbances lasting more than 6 hours
12. Severe pain, sudden edema, paling with cold peripheral limbs and decreased distal pulse
13. Inability to empty bowels or intestine movements (during the previous 24 hours), which cannot be attributed to the main neurological disorder
14. Patient's potentially life-threatening condition - for supervision (head injury, etc.)
15. Severe electrolyte/blood gas /CBC abnormality (any of the following, in mmol/L):
    - 123 < Na > 156
    - 2,5 < K > 6,0
    - 20 < HCO_3 > 36
    - 7.30 < Arterial blood pH > 7.45
    - 20 < CO_2 > 36 (unless chronically abnormal)
    - BUN (blood urea nitrogen) > 15 mg/dL (with increasing dynamics)
    - 3.9 ≤ FBS (Fasting blood sugar) ≥ 22.4 (associated with symptoms of weight reduction for no apparent reasons, polyuria, and polydipsia)
16. ECG evidence of acute ischemia (suspicion of a new myocardial infarction).
17. Acute heart rate disturbances (including ventricular fibrillation) and / or circulation with signs of hemodynamic disorders
18. Signs or symptoms of acute hematological disorders.

B. Intensity of Medical Services

1. Surgical or another procedure requiring equipment or other technologies available only in an inpatient facility (pre-surgery preparation, long – for more than 24 hours – clinical monitoring or supervision)
2. Invasive diagnostic procedures on the central nervous system.
3. Treatment requiring frequent dose adjustments in a potentially severe condition under direct medical supervision

C. Medical Care / Life Support Services

1. Intermittent or continuous use of an artificial lung ventilation at least 3 times per 24 hours.
2. Parenteral therapy - intermittent (2 or more times per day) or continuous intravenous fluid administration with any component / intravenous infusion of medication and / or fluid substitution (does not include tube nutrition).
3. Continuous monitoring of vital signs – at least every 30 mins, for at least 4 hours

D. Other admission criteria

1. Indications for hospitalization defined in the national unified clinical protocols (for specific diseases)
2. Advanced hospitalization with sufficiently documented justification in case of a threat of potential deterioration of the patient's condition (e.g., pregnant women with a high perinatal risk during the perinatal period etc.)
3. The need for isolation in case of an epidemiological or social threat (e.g., from the side of patients with psychological disorders etc.)
4. Other

Note. Hospitalization is not justified for the following cases:

- Provision of care is possible on an outpatient basis or using alternative to inpatient services (day care) irrespective of the remoteness of patient's place of living to the care facility (organization of boarding care for these patients if necessary);
- The patient mostly requires social care (lonely people etc.);
- The decision on hospitalization is made solely based on the referral from the primary care doctor or a specialist doctor;
- The need for hospitalization is caused by rigid rules of the Soviet period (for issuance of a disability status, for the conclusion on ability for military service etc.) and is not associated with the real condition of the patient and intensity of care required.

Duration of inpatient stay

Durations of inpatient stay are defined based on case-specific assessment of the dynamics of a patient condition.

Note. Inpatient stay is considered unjustified in the following cases:

- If a diagnostic test of the patient may be performed without or before patient's hospitalization;
- When based on his/her condition, the patient can continue treatment on a day-care or outpatient basis (irrespective of the remoteness of the patient's place of living from the inpatient facility – boarding care can be organized for such patients if necessary);
- When stay in an inpatient facility is caused by the patient's social problems (lonely people, those who have unsatisfactory living conditions, etc.).
