# Supplementary material for: Assessment of appropriateness of hospitalisations in Ukraine: analytical framework, method and findings
Source: BMJ Open. 2019 Dec 8;9(12):e030081. doi: 10.1136/bmjopen-2019-030081 (PMC6924815; doi:10.1136/bmjopen-2019-030081)
Supplement: Supplementary data [file bmjopen-2019-030081supp002.pdf]

Supplementary file 2

Assessment tool for the appropriateness of hospitalization and hospital stay

CARD FOR EXPERT ASSESSMENT OF THE APPROPRIATENESS OF HOSPITALIZATION AND DURATION OF INPATIENT STAY

The expert assessment uses data from the medical record of the hospital episode (form 003/o), health care facility passport or audit of the secondary (specialized) care (Order of the MoH of Ukraine no. 637 dd. 17.08.2012 "On Approval of the Guidelines for the Procedure for Audit of Secondary (Specialized) Health Care", and other available data. The expert applies criteria of appropriateness of admission and patient stay provided separately.

City/town/village ____

Hospital ____

Department____

Inpatient's medical record no.________________ Date of the expert review _______ ______ ______

Full name of the expert____

I. GENERAL DATA AND CONDITION AT THE TIME OF INPATIENT ADMISSION

1. Age: _________ full years **2. Sex:** 1 – male; 2 – female

3. Residence: 1 – rural 2 – urban

4. Living: 1. With a family (specify the number of people, if possible) ______

2. Alone

3. Difficult to tell

5. Clinical diagnosis at the time of admission:

____

6. Diagnosis at discharge (specify the name and ICD code)

Principal ____ICD_______

Complication of the principal disease ____ICD_______

____

Complication of treatment (incl. post-surgical) ____ICD_______

____

Secondary disease(s) that influenced the course of the principal disease ____

____ICD____

7. Inpatient admission:

1. Planned
2. Urgent - ________hours after the start of the disease or injury

8. During the current year, in relation to the disease, the patient:

1. Is admitted for the first time 2. Has been admitted ________ times

9. The patient is referred by:

1 – primary care specialist

2 – outpatient facility specialist *(indicate specialization)* ________________________________

3 – ambulance unit (emergency medicine)

4 – self-referred

5 – other inpatient facility__________________________ *unit specialization* ______________________________

6 - other *(specify)*______________________________

10. Overall condition of the patient state at the time of admission:

1 – satisfactory

2 – moderate severity

3 – severe

4 – critical

II. APPROPRIATENSS OF ADMISSION

11. Please indicate if the admission is appropriate according to the criteria of the appropriateness of admission:

1. Yes, it is appropriate to this inpatient facility
2. Yes, it is appropriate, but to a higher-level inpatient facility (such as another secondary care facility for intensive care, or a tertiary care facility)
3. Yes, it is appropriate but to another secondary inpatient facility for elective care
4. Inappropriate
5. Difficult to tell

Please provide any comments you have here ____

____

12. What justifies the appropriateness of admission (multiple choice allowed)
1. Intensity of the required and provided services
2. Severity of the case
3. Compliance with existing national clinical protocols
4. Other (specify) ____________________

13. If the admission is not appropriate, or if it is appropriate for another inpatient facility of a different level (see health care facility passport or audit materials), please specify the reasons (multiple choices allowed):
1. Overestimation of the severity of the case (incl. the threat of unexpected progress of the disease)
2. Inpatient admission is likely organized to occupy the beds of the facility
3. Premature admission (e.g., admission on Monday for a surgery planned for Wednesday)
4. There was no time or conditions for a quick examination, or a consultation was required to decide on admission
5. The admission was to reduce the cost of examination or patient's costs, e.g. admission of a patient living far from the hospital to avoid accommodation/hotel costs
6. Ignorance of medical standards of indications for inpatient admission (outdated practice)
7. Admission to a lower level inpatient care was impossible due to funding fragmentation
8. Admission was made at the request of a general practitioner (family doctor) or a specialist doctor.
9. Admission was made on the demand of a patient or his/her family
10. Admission was legally mandatory:
    - 10.1. To receive a medical conclusion on disability
    - 10.2. To receive a medical conclusion on eligibility to military service
    - 10.3. Other (specify) ____________________
11. Admission is related to social problems of the patient
    - 11.1. No opportunity to receive care at home (the patient is lonely, or other family members are disabled)
    - 11.2. Unfavorable conditions at home
    - 11.3. Other (specify) ____________________

14. What measures, in your opinion, could improve the appropriateness of admission in this case (multiple choices allowed):
1. Availability of clear indications/criteria for admission to inpatient facilities of different levels
2. Creation of conditions for diagnostic examination in the outpatient setting or day care facility
3. Removal of financial limitations concerning inpatient admission to a health care facility of the required level
4. Bringing facility's capacity (number of beds) in line with the need of certain type and intensity of care
5. Strict observance of existing unified clinical protocols
6. Creation of an opportunity to perform diagnostic examination in the facility to timely decide whether admission is required
7. Organization of a boarding house at the facility for people from remote locations
8. Elimination of the normative for planning number of personnel linked to the number of beds
9. Clear delimitation of indications to medical and social care
10. Creation of conditions for provision of social care where indicated, incl. round the clock care (development of social care facilities network)
11. Other (specify)____________________

III. JUSTIFICATION OF THE DURATION OF INPATIENT STAY

15. The patient required:
1. Intensive conservative treatment at this inpatient facility
2. Intensive conservative treatment at inpatient facility of a higher level (e.g., s/he was receiving care at a secondary care facility while requiring tertiary care)
3. Intensive conservative treatment at inpatient facility of a lower level (e.g., s/he was receiving care at a tertiary care facility while requiring secondary care)
4. Intensive surgical treatment at this inpatient facility
5. Intensive surgical treatment at inpatient facility of a higher level (e.g., s/he was receiving care at a secondary care facility while requiring tertiary care)
6. Intensive surgical treatment at inpatient facility of a lower level (e.g., s/he was receiving care at a tertiary care facility while requiring secondary care)
7. Elective care at this inpatient facility:
    - 7.1. With the need for the 24/7 medical assistance at the facility;
    - 7.2. Without the need for the 24/7 assistance at the facility

8. Elective care at a lower level inpatient facility
9. Social care:
   9.1. At home
   9.2. With temporary accommodation
   9.3. With permanent accommodation
10. No inpatient care
11. Other (specify) ____

IIIA. Diagnostic Stage of Inpatient Stay

16. Duration of the diagnostic stage (the period from inpatient admission to the start of appropriate treatment).
The decision about the final diagnosis is made on of the _________ day or within ________ hours of stay at the facility

17. Duration of the diagnostic stage:
1. Reasonable 2. Unnecessary long 3. Difficult to evaluate

18. The diagnosis recorded at the inpatient facility was:
1. Correct
2. Incorrect (incorrect disease entity/not all the diseases determined)
3. Inaccurate (incorrect form/stage/extent/severity/clinical manifestations of the disease)
4. Difficult to evaluate

IIIB. Treatment Stage of Inpatient Stay

19. Proper treatment (after confirmation of the final diagnosis) started:
on the ________ day or within ________ hours of stay at the inpatient facility

20. Duration of the treatment period (from the final diagnosis to discharge)
__________ days

21. Surgical interventions*:*
1. None performed 2. Performed (specify)
____Surgical code___________

22. Resuscitation/intensive care
1. No 2. Yes

23. Duration of the treatment stage:
1.Reasonable 2. Unnecessary long 3. Difficult to evaluate

24. <u>If the duration of the inpatient stay was not justified, specify the reasons to support this opinion (multiple choices are allowed</u>):

<u>24.1. General</u>
1. Premature admission
2. The need to occupy excessive beds capacity of the inpatient facility
3. Inadequate or absent clinical protocols
4. Inadequate or absent local protocols, or not adhered
5. Ignorance of medical standards (outdated practice)
6. Admission to a facility where care could not be provided adequately to patient’s condition (e.g., the patient required intensive care, but the facility did not have the equipment for it)
7. Lack of equipment and materials at the facility: no possibility to use modern treatment and diagnostic technology (such as MRI machine for diagnostics, laparoscopic equipment for certain surgical interventions, necessary supplies) – please specify____
8. Lack of medical personnel or skills (specify) ____
9. Inefficient organization of specialized medical consultation
10. Lack of opportunity to use remote telemedical assistance
11. Delays in consent (inability of making fast decisions, hesitation) for the patient and his/her family concerning diagnostic/treatment/surgical procedures
12. Patient’s refusal to receive the suggested treatment
13. Other (specify) ____

<u>24.2. At the diagnostic stage</u>
1. Unreasonable duplication of diagnostic laboratory and instrumental exams conducted at previous stages of care or in other inpatient care setting
2. Potential financial incentives to increase the scope and number of the examination
3. Inefficient schedule of operation of the diagnostic units (laboratory etc.) (such as impossibility of conducting diagnostic tests round the clock)
4. Inefficiencies in the interaction with other facilities required to confirm a diagnosis
5. Other (specify)____

24.3. At the treatment stage

1. Absent/insufficient capacities in terms of life support services (anesthesiology and intensive care department)
2. Lack of practice/organizational issues not allowing to discharge patients after deciding on inappropriateness of inpatient admission based on a preliminary observation
3. Necessity of moving/transporting the patient to another health care facility (reduction of the duration of inpatient stay)
4. Deficit (absence) of alternative care - no capacity or practice of use of day care facilities for post-acute care
5. Complications of the case due to incorrect diagnosis
6. Complications of the case due to inadequate treatment
7. Doctor's overcautiousness regarding the discharge time, incl. due to stereotypes (e.g., discharge after receiving a pathologist's report etc.), please specify ______________________________
8. Inefficient planning of the discharge (e.g., no discharge on week-ends)
9. Premature discharge in order to improve the performance indicators of the facility because of a high probability of death of the patient
10. The patient residing in remote location stayed longer at the facility because s/he was unable to get home on the day of discharge
11. Social problems of the patient:
    11.1. No opportunity to receive care at home (the patient is lonely, or other family members are disabled)
    11.2. Unfavorable conditions at home
12. Other (specify) ______________________________

25. Estimates of the duration of inpatient stay

Treatment stage	***Actual bed-days***	***Recommended bed-days***
**I. Diagnostic**		
**II. Treatment**		
**Total**		

Please provide any comments you have ______________________________

26. Which measures, in your opinion, could improve the appropriateness of inpatient admission and stay (multiple choices allowed):

1. Upgrading anesthesiology and intensive care departments to align with the needs in terms of capacity, equipment and staffing
2. Rightsizing capacities of the facility (e.g. number of beds) according to the needs of the specific type and intensity of care
3. Creation of conditions for diagnostics and follow-up care in a day care or outpatient setting
4. Organization of a boarding house at the facility for people from remote locations requiring post-acute care, provision of care at a day care or in an outpatient setting
5. Addressing financial limitations preventing admission to an inpatient facility of the adequate level
6. Setting up wards for short-term stay for patients in emergency health care department/admission and examination department
7. Better adherence to clinical protocols
8. Clear criteria for discharge from inpatient facilities of different levels
9. Provision of needed materials and equipment
10. Elimination of the normative linking the staff size to the number of beds
11. Clear delimitation of indications to medical (including rehabilitation, hospice) and social care
12. Creation of conditions for provision of social care where indicated, incl. round the clock care (development of social care facilities network)
13. Other (specify) ______________________________

Other comments: ______________________________

Signature of the Expert ______________________________
