# Supplementary material for: Assessment of appropriateness of hospitalisations in Ukraine: analytical framework, method and findings
Source: BMJ Open. 2019 Dec 8;9(12):e030081. doi: 10.1136/bmjopen-2019-030081 (PMC6924815; doi:10.1136/bmjopen-2019-030081)
Supplement: Supplementary data [file bmjopen-2019-030081supp003.pdf]

Supplementary table 1

Inappropriate hospitalizations and unnecessary hospital days (results of the pilot)

Department	Facility	Patients	Bed-days	Inappropriate hospitalizations		Admission to a wrong department/facility		Unnecessary inpatient stay		Inappropriate inpatient stay at wrong departments/facilities	
		Number	Number	Number/%	95% CI	Number/ %	95% CI	Number/% of bed-days	95% CI	Number/% of bed-days	95% CI
**General medicine**	Facility 1	40	426	29/72.5%	58.7% to 86.3%	2/5.0%	-1.8% to 11.8%	327/76.8%	72.7% to 80.8%	12/2.8%	1.2% to 4.4%
	Facility 2	30	297	12/40.0%	22.5% to 57.5%	3/10.0%	-0.7% to 20.7%	120/40.4%	34.8% to 46%	21/7.1%	4.2% to 10%
	Facility 3	30	311	13/43.3%	25.6% to 61.1%	1/3.3%	-3.1% to 9.8%	170/54.7%	49.1% to 60.2%	12/3.9%	1.7% to 6%
	**Total**	**100**	**1034**	**54/54.0%**	**44.2% to 63.8%**	**6/6.0%**	**1.3% to 10.7%**	**617/59.7%**	**56.7% to 62.7%**	**45/4.4%**	**3.1% to 5.6%**
**Cardiology**	Facility 1	41	537	10/24.4%	11.2% to 37.5%	-	-	231/43.0%	38.8% to 47.2%	-	-
	Facility 3	29	249	6/20.7%	5.9% to 35.4%	-	-	70/28.1%	22.5% to 33.7%	-	-
	**Total**	**70**	**786**	**16/22.9%**	**13.0% to 32.7%**	-	-	**301/38.3%**	**34.9% to 41.7%**	-	-
**Neurology**	Facility 1	30	306	2/6.7%	-2.3% to 15.6%	-	-	97/31.7%	26.5% to 36.9%	-	-
	Facility 2	59	645	14/23.7%	12.9% to 34.6%	-	-	253/39.2%	35.5% to 43%	-	-
	Facility 3	30	301	11/36.7%	19.4% to 53.9%	3/10.0%	-0.7% to 20.7%	149/49.5%	43.9% to 55.1%	25/8.3%	5.2% to 11.4%
	**Total**	**119**	**1252**	**27/22.7%**	**15.2% to 30.2%**	**3/2.5%**	**-0.3% to 5.3%**	**499/39.9%**	**37.1% to 42.6%**	**25/2.0%**	**1.2% to 2.8%**
**Surgery**	Facility 1	41	465	5/12.2%	2.2% to 22.2%	-	-	244/ 52.5%	47.9% to 57%	-	-
	Facility 2	30	181	10/33.3%	16.5% to 50.2%	2/6.7%	-2.3% to 15.6%	40/22.1%	16.1% to 28.1%	10/5.5%	2.2% to 8.9%
	Facility 3	21	144	5/23.8%	5.6% to 42.0%	5/23.8%	5.6% to 42.0%	45/31.3%	23.7% to 38.8%	27/18.8%	12.4% to 25.1%
	**Total**	**92**	**790**	**20/21.7%**	**13.3% to 30.2%**	**7/7.6%**	**2.2% to 13.0%**	**329/41.6%**	**38.2% to 45.1%**	**37/4.7%**	**3.2% to 6.2%**
**Total**		**381**	**3862**	**117/30.7%**	**26.1% to 35.3%**	**16/4.2**	**2.2% to 6.2%**	**1746/45.2%**	**43.6% to 46.8%**	**107/2.8%**	**2.3% to 3.3%**
