# Supplementary material for: Assessment of appropriateness of hospitalisations in Ukraine: analytical framework, method and findings
Source: BMJ Open. 2019 Dec 8;9(12):e030081. doi: 10.1136/bmjopen-2019-030081 (PMC6924815; doi:10.1136/bmjopen-2019-030081)
Supplement: Supplementary data [file bmjopen-2019-030081supp004.pdf]

Supplementary table 2

Medical records included in the study

Region / *Level* *Profile*	**Western region**			**Central region**			**Total for 2 regions**		
	Secondary care	Tertiary care	Both levels	Secondary care	Tertiary care	Both levels	Secondary care	Tertiary care	Both levels
Neurology	161	10	171	40	20	60	201	30	231
General medicine	71	40	111	120	50	170	191	90	281
Surgery	40	50	90	80	28	108	120	78	198
Cardiology	20	10	30	20	40	60	40	50	90
Total	292	110	402	260	138	398	552	248	800
