# Supplementary material for: Assessment of appropriateness of hospitalisations in Ukraine: analytical framework, method and findings
Source: BMJ Open. 2019 Dec 8;9(12):e030081. doi: 10.1136/bmjopen-2019-030081 (PMC6924815; doi:10.1136/bmjopen-2019-030081)
Supplement: Supplementary data [file bmjopen-2019-030081supp005.pdf]

Supplementary table 3

The possible reasons for inappropriate hospital admissions and inpatient stays (expert opinions)

Reason	Frequency, % (95% CI)
An attempt to keep the current capacities in the inpatient facility	37.6 (30.5 to 44.6)
Hospital admission is defined by the need to fill inpatient beds and improve their occupancy	22.7 (16.6 to 28.7)
Absence of alternatives to inpatient types of care or capacities to treat patients after their discharge	21.0 (15.1 to 26.9)
Absence of organizational possibilities to discharge patients after a decision is made that it is unnecessary to extend inpatient stay	9.9 (5.6 to 14.3)
Lack of timely examinations or consultations for necessary treatment	12.7 (7.8 to 17.6)
Inefficient cooperation with other professionals and facilities that need to be involved to establish a diagnosis	6.6 (3.0 to 10.3)
Non-adherence to clinical protocols or their outdated provisions	7.2 (3.4 to 10.9)
Overestimated severity of patient's condition	20.4 (14.6 to 26.3)
