# Supplementary material for: Assessment of appropriateness of hospitalisations in Ukraine: analytical framework, method and findings
Source: BMJ Open. 2019 Dec 8;9(12):e030081. doi: 10.1136/bmjopen-2019-030081 (PMC6924815; doi:10.1136/bmjopen-2019-030081)
Supplement: Supplementary data [file bmjopen-2019-030081supp006.pdf]

Supplementary table 4

**Key proposed actions to prevent inappropriate hospital admissions or unnecessary inpatient days**

| Actions | Frequency of suggesting by experts, % (95% CI) |
|---|---|
| To use clear indications or criteria for admission to inpatient facilities of different levels | 32.6 (25.8 to 39.4) |
| To create conditions for diagnostic examinations and post-discharge care [follow-up] in outpatient facilities or on a day-care basis | 44.8 (37.5 to 52.0) |
| To stop using personnel employment normative based on a facility or department's beds capacity | 24.3 (18.1 to 30.6) |
| To bring the network and capacities of inpatient care facilities in compliance with the actual needs in a specific type and intensity of care | 14.9 (9.7 to 20.1) |
| To arrange accommodation/ boarding facilities for persons living far from a treatment facility | 33.7 (26.8 to 40.6) |
| To use clear criteria for patients' discharge from hospitals of different levels | 19.9 (14.1 to 25.7) |
