# Supplementary material for: Assessment of appropriateness of hospitalisations in Ukraine: analytical framework, method and findings
Source: BMJ Open. 2019 Dec 8;9(12):e030081. doi: 10.1136/bmjopen-2019-030081 (PMC6924815; doi:10.1136/bmjopen-2019-030081)
Supplement: Author's manuscript [file bmjopen-2019-030081.draft_revisions.pdf]

BMJ Open

BMJ Open is committed to open peer review. As part of this commitment we make the peer review history of every article we publish publicly available.

When an article is published we post the peer reviewers' comments and the authors' responses online. We also post the versions of the paper that were used during peer review. These are the versions that the peer review comments apply to.

The versions of the paper that follow are the versions that were submitted during the peer review process. They are not the versions of record or the final published versions. They should not be cited or distributed as the published version of this manuscript.

BMJ Open is an open access journal and the full, final, typeset and author-corrected version of record of the manuscript is available on our site with no access controls, subscription charges or pay-per-view fees (http://bmjopen.bmj.com).

If you have any questions on BMJ Open's open peer review process please email info.bmjopen@bmj.com

BMJ Open

Assessment of avoidable hospitalizations in Ukraine: analytical framework, method, and findings

Journal:	*BMJ Open*
Manuscript ID	bmjopen-2019-030081
Article Type:	Research
Date Submitted by the Author:	03-Mar-2019
Complete List of Authors:	Zhao, Feng ; World Bank Group, HNP<br>Doroshenko, Olena; World Bank Group, HNP<br>Lekhan, Valery ; Dnipropetrovsk Medical Academy (DMA), Department of Social Medicine and Health Management<br>Kriachkova, Lilia; Dnipropetrovsk Medical Academy (DMA)<br>Goroshko, Alona; National Health Service of Ukraine, Head of the Department for Development of Benefits Package
Keywords:	hospital admissions, avoidable hospitalizations, unjustified inpatient stay, Ukraine version appropriateness evaluation protocol (AEP), reliability and validity of the method

SCHOLARONE™
Manuscripts

Assessment of avoidable hospitalizations in Ukraine: analytical framework, method, and findings

Feng Zhao, Program Leader, World Bank, Washington, DC.

Olena Doroshenko (corresponding author), Health Specialist, World Bank, 1 Dniprovskiy Uzviz, Kyiv, Ukraine, inglecasket@gmail.com, +38044 490-66-71.

ORCID iD https://orcid.org/0000-0002-0741-2863

Valery N. Lekhan, Professor, Department of Social medicine and health management, Dnipropetrovsk Medical Academy (DMA), Dnipro, Ukraine.

ORCID iD https://orcid.org/0000-0003-2953-3292

Lilia V. Kriachkova, PhD/MD, Professor, Dnipropetrovsk Medical Academy (DMA), Dnipro, Ukraine.

ORCID iD https://orcid.org/0000-0001-7635-2609

Alona Goroshko, Head of the Department for Development of Benefits Package, National Health Service of Ukraine.

Word count, excluding title page, abstract, references, figures and tables – 3661

Keywords: hospital admissions, avoidable hospitalizations, unjustified inpatient stay, Ukraine version appropriateness evaluation protocol (AEP), reliability and validity of the method

For peer review only

ABSTRACT

Objectives: This article reviews the magnitude of avoidable hospitalizations in two regions of Ukraine based on a customized version of the Appropriateness Evaluation Protocol (AEP).

Data and methods: We randomly selected 381 medical records to test the feasibility and reliability of the method and 800 medical records to evaluate the scope of unnecessary hospitalizations. A customized tool was developed following the Delphi procedure, which included criteria for the appropriateness of hospitalization and duration of inpatient stay. We used descriptive and analytical statistics, ROC analysis, and Cohen's kappa to check the consistency between the findings of primary reviewers and experts.

Result: We observed a high level of agreement in conclusions of primary reviewers (reference standard) and experts during testing of the reliability and validity of the method. The external validity check showed that the use of the tool by different experts provided high accuracy: 95.1 sensitivity, 76.6 specificity, and AUC=0.948 ($p<0.001$) for analysis of justifications of admissions; 95.3 sensitivity, 84.7 specificity, and AUC=0.900 ($p=0.001$) for the duration of hospitalizations. Cohen's kappa coefficient (κ) indicated agreement in expert evaluations of 0.915 (95% CI 0.799 to 1.000) and 0.812 (95% CI 0.749 to 0.875), respectively.

We found that over one-third of admissions (38.1%; 95% CI 33.9 to 43.5) and over half of total bed-days were unnecessary (57.4%; 95% CI 56.4 to 58.5). The highest levels of stay were observed in hospitals' therapy departments (64.6%; 95% CI 63.0 to 66.3); other departments included in the analysis – neurology, surgery, and cardiology – had significantly lower ($p<0.05$) levels.

Conclusion. The proposed method is robust in assessing the appropriateness of hospitalizations and durations of inpatient stays. The quantified levels of unnecessary hospital care indicate the need for improving efficiency and quality of care and optimizing the excessive hospital capacities in Ukraine.

ARTICLE SUMMARY

Strengths and limitations of this study

- An objective evaluation of inpatient care is possible through application of a unified method for the assessment of the appropriateness of hospital admissions and duration of hospitalizations, which is customized for Ukraine and is based on clear criteria for in-patient treatment.
- The received data on the scope of unnecessary hospitalizations and their excessive duration provide a reliable base to estimate the needs of Ukraine's population in hospital care, which is especially relevant for the current reform of the health sector in the country.
- The study suggests that the criteria for inpatient admissions should be included in contracts with hospital care providers in the course of the health financing reform to maximize efficiency and ensure appropriate quality of care.
- Because electronic medical records were not available, the quality of the assessment of paper-based medical records was compromised by the structure and legibility of hand-written text.
- The future use of the proposed method in practice may require additional training for evaluators; its sustainability will depend on uptake of the method at the national and at facility levels.

For peer review only

INTRODUCTION

Hospital care is a core part of any national health system; it influences health outcomes of the population and requires significant funding. [1, 2] Still, use of hospital care has distinct features across the globe and ranges in terms of level of effectiveness. [3] If inpatient care fails to improve the health outcome when compared with less invasive or less intensive non-hospital care, it is considered an overuse of inpatient services. [4, 5, 6]

Overuse of hospital care is typical for many countries around the world, ranging from 1% to 54% in general [5], and up to 90% for some diseases. [7] Unnecessary hospitalizations are a matter of concern for many nations, specifically due to the rising trend of healthcare expenditures and a significant proportion of hospital expenditures in overall health spending. [8 - 12]

Hospital sector expenditures are mostly dependent on admission processes, length of patient hospital stays, and advancement of medical technologies in hospital facilities. Inpatient length of stay (LOS) is the most important contributing factor influencing the use of resources and cost of care during hospitalization; it closely relates to the effectiveness of hospital treatment, the effectiveness of hospital management, and general efficiency of a health system. [13, 14, 15]

Rationalization of hospital expenditures together with increased, or at least sustained, quality of hospital care are key objectives of health care reform in Ukraine. [15] The current trends of poor health outcomes and suboptimal allocation of resources explain the importance of this objective. Ukraine is a middle-income country with $2,639.82 GDP per capita (2017) located in the Eastern Europe. It is behind other middle-income countries in terms of health sector performance. The average life expectancy at birth in 2017 was 72 years in Ukraine, 9 years less than in countries of the European Union (EU) and Organization for Economic Co-operation and Development (OECD). [17]

The hospital sector in Ukraine is oversized and inefficient by both the numbers of beds and hospitals. The number of beds per 10,000 people was 74.3 in 2016, or three times higher than in the EU; the number of hospitals per 10,000 people exceeded EU numbers by a quarter; the average length of stay was 11.2 days, or 3 days longer than for patients in the EU. [18]

Hospital expenditures are relatively high. Hospitals are responsible for about one-third of total health expenditures (34.2% in 2016), [17] which is slightly lower than the OECD average (38% in 2015). [3] Between 82% to 92.7% [19, 20] of patients, however, pay out-of-pocket for medications needed during hospital treatment. If these costs are included,

For peer review only - http://bmjopen.bmj.com/site/about/guidelines.xhtml

the share of hospital expenditures rises to over 50%. Until just recently, hospital budgets followed a historical Semashko system in which hospital funding was dependent on the number of beds. This budgetary system produced perverse incentives to preserve beds and maintain high bed occupancy rates, regardless of actual need for hospital care, and resulted in cost inefficiencies. [16]

Improvement of hospital efficiency has been among the health policy priorities since Ukraine gained independence in 1991. A new hospital financing reform is planned for 2019-2020. Numerous studies have been dedicated to the use of hospital care, [21 - 27] and the research has shown significant inefficiencies in the hospital sector. Reported results, however, vary substantially: between 8.2 and 70.6% unjustified hospitalizations, and 26.4 - 86.5% of unnecessary bed-days. [21 - 27]

Studies in other countries have reported similar problems with variability of results [7, 8, 14], which led to an international recommendation from researchers that a unified method for the assessment of reasons for hospitalizations and their duration should be used, [7, 8] specifically the Appropriateness Evaluation Protocol (AEP). [28, 29] The AEP has high validity and reliability compared with other methods for assessment of inappropriate hospitalizations [2, 30] and was customized for many countries. [7, 8, 31]

The AEP method has not been used in Ukraine, where assessments are traditionally based on expert evaluations providing retrospective analyses of medical records. Such analyses are typically designed by individual experts through methods that are not validated. [21 - 27] Although the provision of hospital care is guided by legislation and clinical protocols, [18] concrete and evidence-based criteria for hospitalizations are absent.

The objective of this study was to develop and test the customized Ukraine version of the unified method for assessment of the appropriateness of hospitalizations, including reasons for admissions to hospitals and duration of hospital stays, and to evaluate the scope of inappropriate hospitalization of adults in Ukraine using a sample of hospitals from two regions.

METHODS

Ethics statement

Confidentiality of information from medical records was kept by protecting patient identifiers. Researchers received copies of paper-based records with patient identification fields crossed out or covered. Names of physicians and hospital names were replaced with ID codes for reviewers of medical records.

All experts working with primary data signed non-disclosure agreements of confidential data and obligations to follow ethics principles of medical research statements, which are endorsed by international agreements and the Ministry of Health of Ukraine. Compliance with bioethics principles and medical deontology was confirmed in the conclusion of the Committee on bioethics and medical deontology in the State Institution "Dnipropetrovsk Medical Academy of the MOH Ukraine" (Protocol No.6 of September 16, 2016).

Patient and Public Involvement

We did not involve patients or the public in our work.

Tool development

Based on available international tools [7, 8, 28 - 33] and existing approaches used in Ukraine, [21 - 27] the tool for this study was developed and customized for the Ukrainian context in order to assess appropriateness of hospitalizations and duration of inpatient stays. The tool included criteria for appropriateness of hospital admissions and a guide for expert evaluations of justified inpatient lengths of stay. The tool was finalized using the Delphi method for expert evaluations [34] in three rounds as schematically presented in figure 1.

Criteria for hospital admissions were included in the customized tool based on an analysis of seven versions of the AEP: original US version (US-AEP), [28] European (E-AEP), [29] Turkish (TR-AEP), [32] Dutch (D-AEP), [31] German (DE-AEP), [33] Chinese (C-AEP) [8], and Iranian version of the AEP (IR-AEP). [7] The customized Ukrainian AEP tool defined Ukraine-specific indications for hospital admission and criteria for justification of inpatient length of stay – summarized in the Ukrainian Appropriateness Evaluation Protocol (UA-AEP) (see online supplementary file 1).

We used criteria from these versions of the UA-AEP to develop a guide that helps to extract medical information about hospital care episodes from medical records (MoH form 003/o) for evaluation of the appropriateness of admission decisions and justification of lengths of stay (see online supplementary file 2).

The following statistics describe the experts' agreement on the correctness of the customized tool proposed for the assessment of the appropriateness of hospitalizations: the criteria for hospital admissions concordance between evaluators was W=0.96 ($p<0.01$), Cronbach's alpha, α=0.943, approached the maximum possible level for reliability of repeated measurements; on the guide for evaluation of appropriateness of admission decisions and justification of length of stay, W=0.98 ($p<0.05$), Cronbach's alpha, α=0,926.

Piloting of the method

The proposed tool was piloted in three types of hospitals of different size in one of the largest regions in eastern Ukraine. Four department profiles were selected for the analysis: therapy (equivalent to general medicine department), cardiology, neurology, and surgery. These departments are representing a most typical structure of a hospital: on average, 44% of all adult patients are admitted to these departments in Ukraine.

Standard medical records were used for analysis (MoH approved Form 003/o). The two-stage approach was used to sample records for the year preceding the analysis: first, one month was randomly selected (March 2016); second, from all archived records in the four departments in March 2016, 381 cases were randomly selected. All sampled records were proportionally distributed for evaluation to 38 experts with relevant clinical backgrounds for the profile of sampled cases. Experts were selected based on their area of expertise and the results of the initial test of competencies. The total number of experts was determined based on an average anticipated workload of 10 medical records per one expert.

The results of the piloting confirmed the feasibility of the tool for data collection and analysis. The pilot data showed that about one-third of all admissions included in the analysis were classified as avoidable. The length of stay in facilities was often excessive: 45.2% of all bed-days were avoidable (see online supplementary table 1). The yielded results corresponded with the finding from a study of avoidable hospitalization in the same region, [23] and this confirmed the feasibility of the proposed method.

Reliability and validity of the method

The results of the pilot were verified by a review panel, consisting of three primary reviewers (public health scientists). Each primary reviewer was assisted by a clinician in the relevant clinical field (therapist, surgeon, and neurologist).

We assessed the reliability and validity of the method by comparing the evaluation results provided by 38 experts with the conclusions of the review panel. The latter was used as a reference standard for the conclusions.

The level of matching between the two groups (experts and review panel) was analyzed using the coefficient of the overall agreement and Cohen's kappa coefficient (κ). We used ROC analysis to calculate sensitivity and specificity of the method and area under ROC curve (AUC) to show classification capacity of the method to define appropriateness of admissions and their duration.

Statistical methods

The dataset and statistics were prepared using Microsoft Excel (Microsoft Office 2016 Professional Plus, Open License 67528927) and STATISTICA 6.1 software (StatSoftInc.,

serial No. AGAR909E415822FA). We used descriptive and analytical statistics for data analysis. [35] For relative values, 95.0% confidence intervals (95% CI) were calculated based on the corrected Wald method. The assessment of the validity in the differences in relative indices was carried out according to Pearson's Chi-square test (Chi-square test – χ^2).

For the sample calculations, we used Cochran formula with p=0.95 (α=0.05) and t=1.96. The general number of hospital admissions N for the pilot sites was 755,350 and the hospital admission rate 23.3%, making the calculation for the minimal powered sample of 275 admissions for the pilot (sampled 381). For the main stage of the analysis, the recommended sample of 322 records (α=0,05) or 895 records (α=0,03) was calculated using "Power analysis" in STATISTICA 6.1 software for the general number of admissions in Ukraine of 8,594,210, and the level of inappropriate hospitalizations of 29.9% calculated for the pilot (sampled 800).

To analyze agreement in experts' reviews, we used the concordance coefficient (W) and Cronbach's alpha (α). Approximation of coefficient values to 1 indicates an increase in agreement rates.

The inter-rater agreement in experts' reviews was analyzed using overall agreement coefficient and Cohen's kappa coefficient (κ), applying the following ranges: values of 0.61-0.80 were defined as good strength of agreement, values of 0.81 and higher as very good strength of agreement. [35]

ROC curve analysis was conducted to calculate operational characteristics and the area under ROC-curve to evaluate discriminative capacity of the method. The relationship between the area under the ROC curve and diagnostic accuracy was assessed using the following ranges: 0.7-0.8 – good; 0.8-0.9 – very good; 0.9 – 1.0 – excellent. [36]

The statistical significance was taken at the level of p <0.05 (5%) for all tests.

RESULTS

Results of Reliability and Validity tests

We conclude that the pilot of the method proved its reliability and validity for the assessment of the hospitalizations because sensitivity and specificity measures are high for the sample and within the selected medical specialties for both evaluation of the appropriateness of admissions and duration of inpatient stay (Table 1).

Table 1

Inter-rater agreement of experts' evaluations and Operational Characteristics of the Method for Assessment of the Appropriateness of Hospitalizations

Parameter	Therapy	Cardiology	Neurology	Surgery	Total pilot

	n=100	n=70	n=119	n=92	sample n=381
Appropriateness of admissions					
Sensitivity (%)	84.8	96.3	96.4	97.3	95.1
Specificity (%)	73.1	87.5	68.5	94.7	76.6
Area under ROC curve (p-value)	0.865 (0.002)	0.977 (<0.001)	0.945 (<0.001)	0.991 (<0.001)	0.948 (<0.001)
Overall agreement (%)	84.0	94.2	88.2	90.5	92.8
Cohen's κ (95% CI)	0.702 (0.566 to 0.839)	0.916 (0.871 to 0.961)	0.888 (0.781 to 0.996)	0.934 (0.843 to 1.000)	0.915 (0.799 to 1.000)
Duration of inpatient stay					
Sensitivity (%)	88.4	96.3	96.6	97.2	95.3
Specificity (%)	85.9	87.5	75.0	94.4	84.7
Area under ROC curve (p-value)	0.870 (0.009)	0.920 (<0.001)	0.860 (0.007)	0.960 (<0.001)	0.900 (0.001)
Overall agreement (%)	86.7	91.9	90.1	94.3	91.2
Cohen's κ (95% CI)	0.737 (0.604 to 0.870)	0.838 (0.684 to 0.992)	0.772 (0.639 to 0.905)	0.902 (0.794 to 1.000)	0.812 (0.749 to 0.875)

Using Cohen's kappa (κ), we observed a high level of agreement in experts' reviews. Results for agreement on the appropriateness of admissions was 0.915 (95% CI (0.799 to 1.000) and duration of inpatient stay was 0.812 (95% CI 0.749 to 0.875).

The proposed method has very high operational characteristics: the area under the ROC curve only slightly varies for different medical specialties and averages to 0.948 for the appropriateness of admissions and to 0.900 for the duration of stay, which defines the potential to properly distinguish different patterns (discriminative capacity) of the method as excellent.

Core sample

Admissions at inpatient departments of secondary and tertiary care facilities were studied in two regions of Ukraine (central and western). The following 12 healthcare facilities were included in the study: in the central region – 2 regional (tertiary) and 5 city and district (secondary) healthcare facilities; in the western region – 1 regional (tertiary) and 4 city and district (secondary) healthcare facilities. The total number of discharged patients in the sampled facilities amounts to about 110,000 annually.

The expert assessment covered a random sample of 800 medical records in four medical specialties: therapy, neurology, surgery, and cardiology (Supplementary table 2). A similar approach was used to sample records for the core sample as in the pilot. First, all

For peer review only

medical records for patients discharged during the month preceding the beginning of data collection (July 2016) were collected from all departments of the therapy, surgery, neurology, and cardiology profile. Second, for each specialty department in each facility, the medical records were sampled randomly according to the discharge date. The total number of sample medical records made up 8% to 12% of the total number of patients discharged from the selected departments during that month.

The sampled medical records were proportionally distributed among experts according to their specialization. Experts reviewed medical records following the agreed guide for the analysis of the appropriateness of hospitalizations.

Results of data analysis for two regions of Ukraine

The findings of the expert review show that over one-third of cases – 38.1% (95% CI 33.9 to 43.5) in the central region, 38.7% (95% CI 34.8 to 41.5) in the western region and 37.6% (95% CI 32.8 to 42.3) in total were unjustified hospital admissions (figure 2). Every seventh patient (13.3%; 95%CI 6.5% to 20.8%) admitted to an inpatient department of tertiary care facilities could have been more appropriately treated at secondary care facilities with adequate equipping and staffing.

In our sample, over half of the total bed days spent by patients in hospitals were classified as avoidable/longer than necessary (57.4%). The volume of unjustified bed-days in the western region were far greater (63.1%) than in the central region (51.7%, p<0.001), which was related to a higher rate of unjustified admissions at secondary care facilities of patients to all specialized departments except the neurology profile (table 2).

Table 2

Volumes of unnecessary hospitalizations

Characteristic of the sample	Number of inappropriate admissions	Total bed-days for inappropriate hospitalizations	Average length of stay in bed-days (95% CI)	Avoidable days of inpatient stay, % (95% CI)
**Total for 2 regions**				
By facility level				
Secondary care	217	2255	7.1 (6.9 to 7.2)	58.8 (57.5 to 60.1)
Tertiary care	88	1274	8.2 (7.8 to 8.5)	54.8 (52.9 to 56.6)#
Both levels	305	3530	7.4 (7.3 to 7.5)	57.4 (56.4 to 58.5)
By specialty of departments				
Neurology	79	1123	7.9 (7.7 to 8.0)	54.6 (52.6 to 56.5)°
Therapy	151	1083	8.8 (8.6 to 9.0)	64.6 (63.0 to 66.3)
Surgery	54	838	5.9 (5.7 to 6.0)	52.4 (50 to 54.7)°
Cardiology	21	486	7.3 (7.0 to 7.5)	51.2 (48.1 to 54.3)°
**Central region**				
By facility level				
Secondary care	105	1252	8.2 (8 to 8.4)	50.6 (48.7 to 52.6)*

Tertiary care	49	740	8.5 (8.3 to 8.7)	53.4 (51.0 to 55.9)
Both levels	154	1993	8.3 (8.2 to 8.4)	51.7 (50.1 to 53.2)*
By specialty of departments				
Neurology	79	1123	7.9 (7.7 to 8.0)	55.0 (51.2 to 58.8)
Therapy	151	1083	8.8 (8.6 to 9.0)	58.4 (56.1 to 60.7)*
Surgery	54	838	5.9 (5.7 – 6.0)	41.9 (38.9 to 44.9)*°
Cardiology	21	486	7.3 (7.0 to 7.5)	46.6 (42.9 to 50.2)*°
**Western region**				
By facility level				
Secondary care	112	1002	6.0 (5.7 to 6.3)	65.9 (64.2 to 67.6)
Tertiary care	39	533	7.7 (6.6 to 8.8)	56.6 (53.8 to 59.4)#
Both levels	151	1535	6.5 (6.3 to 6.7)	63.1 (61.7 to 64.6)
By specialty of departments				
Neurology	58	830	7.8 (7.5 to 8.2)	54.4 (52.1 to 56.7)°
Therapy	59	366	7.8 (7.3 to 8.3)	72.7 (70.3 to 75.1)
Surgery	27	237	3.8 (3.4 to 4.3)	67.4 (63.9 to 70.8)
Cardiology	7	102	4.9 (4.1 to 5.6)	63.0 (57.3 to 68.7)°

Notes: * - p<0.001 compared with the other region
# - p≤0.001 compared with the secondary level facilities
° - p<0.001 compared with the therapy departments

The analysis of appropriateness of hospital admissions by specialties showed that the highest level of unjustified admissions was recorded for patients treated in therapy departments (53.7%); lower levels (p<0.001) were identified for all the other specialties: neurology – 34.2%; surgery – 27.3%; and cardiology – 23.3%.

The highest share of unjustified stay was determined for patients of therapy departments (64.6%), and the lowest share in cardiology (51.2%); for the neurology and surgery departments these shares were also high at 54.6% and 52.4% of total bed-days respectively.

The experts were asked to specify one of the proposed reasons for unjustified hospitalization in each medical record reviewed. The following statistics describe the major reasons of inappropriate hospital admissions and unjustified inpatient stays: to keep patients under care in the absence of other facilities with capacity to provide care to patients after discharge (21.0%; 95% CI 15.1 to 26.9), and to treat patients with overestimated severity of their condition (20.4%; 95% CI 14.6 to 26.3) (see Supplementary table 3 for all reasons).

DISCUSSION

Our study generates two key results regarding hospital care in Ukraine. First, there is a need for accurate assessment regarding appropriate hospital admissions. To increase the objectivity of assessing the use of hospital resources, we suggest applying a unified method, customized for Ukraine, which gives reliable results for analyzing the

For peer review only

appropriateness and duration of hospital admissions. Second, the reserves for optimization of the hospital sector in Ukraine are high, as evidenced by the volume of inappropriate hospitalizations in the two regions estimated using the proposed method.

There are several limitations that may limit the generalizability of the findings related to possible seasonal variations for hospital care statistics and selection of regions that may not be fully representative for the whole of Ukraine. To minimize and adjust for limitations, we compared the sample data from two regions with the data collected from an additional region during the pilot stage. We did not find any significant differences between the regional statistics: the level of inappropriate admissions in the pilot was 30.7% (95% CI 26.0 to 35.4), which is slightly lower than the total for the surveyed regions but could be explained by the inclusion of municipal hospitals in the pilot, which had lower levels of inappropriate hospitalizations in the main sample as well. We, therefore, assume that the facilities in our research are representative of inpatient facilities in Ukraine.

The results of our study point to a high volume of unnecessary hospitalizations and their unjustified duration in Ukraine. The number of unjustified bed-days for appropriately hospitalized patients exceeded the number of bed-days from inappropriate admissions to the hospital by 27.8% (95% CI 24.4 to 31.1). This means that appropriately hospitalized patients often stay in the hospital longer than necessary. This share is especially large for patients hospitalized in therapy departments – the numbers of unjustified bed-days for appropriately admitted patients exceeds the number of bed-days for inappropriately admitted patients by 37.0% (95% CI 33.7 to 40.2).

We asked experts engaged in the evaluations to propose financial, economic, and organizational measures to prevent inappropriate hospitalizations or excessively long in-patient treatment (Supplementary table 4). Close to a quarter of respondents (24.3%; 95% CI 18.1 to 30.6) proposed to eliminate the normative regulation specifying the number of staff based on the number of beds in the facility. This principle, inherited from the Soviet era, incentivized hospitals to maintain a large number of beds regardless of the actual needs for inpatient care. Although the issue had been included on the reform agenda for several decades, [15] it was only addressed in 2016 when the Ministry of Health abolished the existing normative regulation. This change, however, has not yet resulted in significant optimization of hospital capacities.

Hospitals in Ukraine do not differentiate by the intensity of interventions. In most hospitals, both acute and chronic patients, as well as those who need nursing and social care, can stay in the same departments to receive medical care. Another proposed measure for improvement of hospital efficiency was to right size the hospital network to align it with

actual need in hospital care and categorize capacity of hospitals by intensity of care (14.9% of respondents, 95% CI 9.7 to 20.1), as well as the use of clear indications for admission to inpatient departments for facilities of different levels (32.6%, 95% CI 25.8 to 39.4). Such measures were proposed at the preliminary stage of the health reforms (2010-2014) [15] but not yet implemented.

The most popular measure proposed by experts was the introduction of alternatives to inpatient care: the replacement of inpatient services with outpatient services provided at the primary care level and the organization of treatment services without overnight stay (44.8% of respondents, 95% CI 37.5 – 52.0). This suggestion is in line with the current global trend of changes in the health system organization. [37, 38] Currently, the avoidance of hospitalizations of patients with ambulatory care-sensitive conditions (ACSCs) is included as a special policy direction on the agenda of many countries. [25, 39, 40, 41] The rate of avoidable hospitalization can be seen as an indicator of the quality of primary health care. [42, 43] Larger capacity of primary care to treat ACSCs results in a significant reduction of the hospitalizations of patients with these conditions. [44] Day-care clinics and day-care centers, as well as socially appropriate and less expensive forms of medical care, are widely used in different countries as alternatives to hospital services. [3, 45, 46]

To increase the availability of alternative care for patients from remote areas, an organization of day care coupled with discharge to a boarding house facility has proven to be effective. [47]. In such facilities, a patient, after receiving the relevant service or intervention, is placed in a boarding house where a range of services are available, including capacity for the provision of necessary care in the case of complications. This approach was favored by 33.7% of respondents (95% CI 26.8 to 40.6) [47]

Daycare facilities are also gaining popularity in Ukraine. In 2016, 31.6% more patients received medical care in daycare facilities compared with the total number of hospitalized patients, but the increase in the use of daycare facilities did not replace inpatient care. [15] According to large-scale international studies, the combination of incentives and special training of medical staff can help to improve use of daycare instead of hospitalizations. [48, 49]

An additional measure is the timely discharge of patients from hospitals (proposed by 19.9% of experts, 95% CI 14.1 to 25.7). The issue of appropriate discharge is not unique to Ukraine. European experts point out that there are obstacles to timely discharge in multiple countries. [1]

For peer review only

The current health reform prepared and launched by the Government and the Ministry of Health in 2018 focuses primarily on changing the payment mechanisms, which will influence changes in the hospital sector. At the same time, reforms also cover the primary health care and emergency services to ensure accessibility of services within the scope of limited available resources in the country.

Optimization of inpatient care by reducing unnecessary hospitalizations and excessive lengths of stay in hospitals, given the current economic situation, may become more than just an important direction in the development of the health care system to increase effectiveness. It can essentially help to sustain good progress in the improvement of health sector organization and health outcomes by adequately meeting the needs of the population in hospital care. Although the suggested transformations of the hospital sector may require additional funds for structural reorganization, training, and motivation of medical staff, these investments are likely to be offset by the large efficiency gains from hospital optimization.

For peer review only

FOOTNOTES

Acknowledgments

The authors thank all staff working in Health Departments of the two regions who facilitated the process of data collection, assisted with the preparation of the primary dataset, and participated in the discussion for data interpretation.

Data statement

Technical appendix for dataset available from the Dryad repository, DOI:10.5061/dryad.1td0rm3

Authors' Contributions:

F. Zhao – Critical review of the article for important intellectual content,

O. Doroshenko - Drafting the article, preparing the first concept of the study,

V. Lekhan - Substantial contribution to concept and design of the study, overall guidance and coordination of the study,

L. Kriachkova - Substantial contribution to acquisition of data, analysis and interpretation of data, and the first draft of the article,

A. Goroshko – Contributions to the first draft of the article.

Competing Interests

None declared

Funding and all other required statements

The study was supported by the World Bank (within Improving effectiveness in human development and social accountability Project financed by the Department for International Development).

Figures and supplementary files included in the manuscript

Figure 1 Schematic presentation of the steps followed during the development and evaluation of the method

Figure 2 Shares of inappropriate admissions by department specialties, total and in the two regions of Ukraine (%, 95% CI)

Supplementary file 1 Criteria for appropriateness of hospital admissions and length of inpatient stay

For peer review only

Supplementary file 2 Assessment tool for appropriateness of hospitalization and hospital days

Supplementary table 1 Avoidable hospitalizations and hospital days (results of the pilot)

Supplementary table 2 Description of the medical records included in the study

Supplementary table 3 The possible reasons of unjustified hospital admissions and inpatient stays (expert opinions)

Supplementary table 4 Key proposed actions to prevent unjustified hospital admissions or unnecessary inpatient days

For peer review only

REFERENCES

1. McKee M, Healy J. Hospitals in changing Europe. (European Observatory on Health Care Systems series). Buckingham: Published by Open University Press 2002: 295.

2. McDonagh MS, Smith DH, Goddard M. Measuring appropriate use of acute beds. A systematic review of methods and results. Health Policy. 2000;53(3):157–84.

3. Health at a Glance 2017: OECD Indicators. Paris: OECD Publishing. http://dx.doi.org/10.1787/health_glance-2017-en.

4. Saltman RB, Durán A, Dubois HFW. Governing Public Hospitals. Reform strategies and the movement towards institutional autonomy. World Health Organization: European Observatory on Health Systems and Policies 2011: 261. http://www.euro.who.int/__data/assets/pdf_file/0017/154160/e95981.pdf?ua=1.

5. Brownlee S, Chalkidou K, Doust J, et al. Evidence for overuse of medical services around the world. *Lancet*. 2017;390(10090):156-168. Published online 2017 Jan 9. doi: [10.1016/S0140-6736(16)32585-5].

6. Maslow K, Ouslander JG. Measurement of potentially preventable hospitalizations. Prepared for the long-term quality alliance. February 2012: 86.

7. Purdy S, Griffin T. Reducing hospital admissions. *BMJ*. 2008 Jan 5; 336(7634): 4–5. doi: [10.1136/bmj.39394.402465.BE]

8. Esmaili A, Ravaghi H, Seyedin H, et al. Developing of the Appropriateness Evaluation Protocol for Public Hospitals in Iran. *Iran Red Crescent Med J*. 2015 Mar; 17(3): e19030. Published online 2015 Mar 1. doi: [10.5812/ircmj.19030]

9. Liu W, Yuan S, Wei F, et al. Reliability and Validity of the Chinese Version Appropriateness Evaluation Protocol. *PLoS One*. 2015;10(8):e0136498. Published 2015 Aug 25. doi:10.1371/journal.pone.0136498.

10. Pandey A, Ploubidis GB, Clarke L, et al. Hospitalisation trends in India from serial cross-sectional nationwide surveys: 1995 to 2014. *BMJ Open*. 2017;7(12):e014188. Published 2017 Dec 19. doi:10.1136/bmjopen-2016-014188.

11. Kastor A, Mohanty SK. Disease and age pattern of hospitalisation and associated costs in India: 1995–2014. *BMJ Open* 2018;8:e016990. doi:10.1136/ bmjopen-2017-016990

12. Duckett S, Breadon P. Controlling costly care: a billion-dollar hospital opportunity. Grattan Institute Report No. 2014-2. March 2014: 51.

13. Banham D, Chen T, Karnon J, et al. Sociodemographic variations in the amount, duration and cost of potentially preventable hospitalisation for chronic conditions among

For peer review only

Aboriginal and nonAboriginal Australians: a period prevalence study of linked public hospital data. *BMJ Open* 2017;7:e017331. doi:10.1136/ bmjopen-2017-017331

14. Kulinskaya E, Kornbrot D, Gao H. Length of stay as a performance indicator: robust statistical methodology. *IMA Journal of Management Mathematics* 2005;16:369–81.doi:10.1093/imaman/dpi015.

15. Nekoei MM, Amiresmaili M, Goudarzi R, et al. Investigating the Appropriateness of Admission and Hospitalization at a Teaching Hospital: A Case of a Developing Country. *Iran J Public Health*. 2017;46(12):1720-1725.

16. Lekhan VN, Rudiy VM, Shevchenko MV, et al. Ukraine: Health system review. Health Systems in Transition, 2015; 17(2): 1–153. http://www.euro.who.int/__data/assets/pdf_file/0018/280701/UkraineHiT.pdf

17. European Health for All database (HFA-DB). https://gateway.euro.who.int/en/datasets/european-health-for-all-database. Updated: 15 June 2018.

18. National Health Accounts of Ukraine in 2016: Statistical Bulletin. Kyiv: State Statistics Service of Ukraine 2018: 172.

19. Stepurko TG, Semigina TV. Health Index. Ukraine-2017. Kiev 2018: 98-134. http://health-index.com.ua/upload/files/+Report%20Health%20Index%202017_web.pdf784933025.pdf.

20. Self-assessment by the population of the health status and availability of certain types of medical care in 2017 (according to a sample household survey in October 2017). Kyiv: State Statistics Service 2018: 148. http://www.ukrstat.gov.ua/.

21. Slabkij GO, Podoljaka VL. Use of material and technical resources as the basis of the reform of the stationary health sector of a large industrial city. *Visnyk social'noi' gigijeny ta organizacii' ohorony zdorov'ja Ukrai'ny*. 2004;2:62-66.

22. Smijanov VA. Analysis of outpatient clinics and inpatient facilities in a modern large city (for example, Sumy). *Visnyk social'noi' gigijeny ta organizacii' ohorony zdorov'ja Ukrai'ny*. 2004;2:8-61.

23. Lehan VM, Shherbinina MB, Volchek VV. Reserves for improving efficiency in the stationary health sector. *Medychni perspektyvy*. 2006;3:73-81.

24. Gapon VO, Lysak VP, Durdykulijeva NI. Expert assessment of the quality of provision of medical care at the secondary level in the agro-industrial region. *Ohorona zdorov'ja Ukrai'ny*. 2008;4(32):22-23.

For peer review only

25. Lekhan VM, Hinzburh VG, Kriachkova LV, et al. A new mode of primary health care--pilot regions experienc. *Wiad Lek*. 2014;67(2 Pt 2):210-4.

26. Pariy VD, Hryshchuk SM, Borys VM. The main approaches to the definition of a hospital intensive care when creating hospital districts *Scientific Journal «ScienceRise»*. 2015;10/3 (15): 97-100.

27. Bugro VI. Study of validity of hospitalization and bed capacity use of specialized hospital health institutions of secondary medical care. *Visnyk naukovyh doslidzhen'*. 2016;1:6-9. doi 10.11603/1681-276X.2016.1.6086.

28. Gertman PM, Restuccia JD. The appropriateness evaluation protocol: a technique for assessing unnecessary days of hospital care. *Med Care*. 1981 Aug;19(8):855-71.

29. Lang T, Liberati A, Tampieri A, et al. A European version of the Appropriateness Evaluation Protocol. Goals and presentation. The BIOMED I Group on Appropriateness of Hospital Use. *Int J Technol Assess Health Care*. 1999 Winter;15(1):185-97.

30. Strumwasser I, Paranjpe NV, Ronis DL, et al. Reliability and validity of utilization review criteria. Appropriateness Evaluation Protocol, Standardized Medreview Instrument, and Intensity-Severity-Discharge criteria. *Med Care*. 1990 Feb;28(2):95-111.

31. Panis LJ, Verheggen FW, Pop P. To stay or not to stay. The assessment of appropriate hospital stay: a Dutch report. *Int J Qual Health Care*. 2002 Feb;14(1):55-67.

32. Kaya S, Erdem Y, Doğrusöz S, et al. Reliability of a hospital utilization review method in Turkey. *Int J Qual Health Care*. 1998 Feb;10(1):53-8.

33. Sangha O, Schneeweiss S, Wildner M, et al. Metric properties of the appropriateness evaluation protocol and predictors of inappropriate hospital use in Germany: an approach using longitudinal patient data. *Int J Qual Health Care*. 2002 Dec;14(6):483-92.

34. Dalkey NC. The Delphi method: An experimental study of group opinion. Santa Monica : Rand.org. 1969: 79. http://www.rand.org/content/dam/rand/pubs/research_memoranda/2005/RM5888.pdf.

35. Altman DG. Practical statistics for medical research. London : Chapman and Hall 1991: 611.

36. Measures of Diagnostic Accuracy: Basic Definitions. *EJIFCC*. 2009;19(4):203-11. Published 2009 Jan 20.

37. McPherson K, Gon G, Scott M. International Variations in a Selected Number of Surgical Procedures. Health Working Papers No. 61. Paris : OECD Publishing 2013. http://dx.doi.org/10.1787/5k49h4p5g9mw-en.

For peer review only

38. Nolte E, McKee M. Caring for people with chronic conditions. A health system perspective. New York : Open University Press 2008: 290.

39. Freund T, Campbell SM, Geissler S, et al. Strategies for Reducing Potentially Avoidable Hospitalizations for Ambulatory Care–Sensitive Conditions. *Ann Fam Med*. 2013 Jul-Aug;11(4):363-70. doi: 10.1370/afm.1498.

40. Assessing health services delivery performance with hospitalizations for ambulatory care sensitive conditions. Working document. World Health Organization : Regional Office for Europe April 2016: 52. http://www.euro.who.int/__data/assets/pdf_file/0010/305875/Assessing-HSD-performance-with-ACSH.pdf.

41. Schüssler-Fiorenza Rose SM, Stineman MG, Pan Q, et al. Potentially Avoidable Hospitalizations among People at Different Activity of Daily Living Limitation Stages. *Health Serv Res*. 2017 Feb;52(1):132-155. doi: 10.1111/1475-6773.12484. Epub 2016 Mar 17.

42. Chun SY, Kim W, Park EC. Disparities in avoidable hospitalization by income in South Korea: data from the National Health Insurance cohort. *Eur J Public Health*. 2018 Sep 26. doi: 10.1093/eurpub/cky198.

43. Afonso MPD, Shimizu HE, MerchanHamann E, et al. Association between hospitalisation for ambulatory care-sensitive conditions and primary health care physician specialisation: a cross-sectional ecological study in Curitiba (Brazil). *BMJ Open* 2017;7:e015322. doi:10.1136/ bmjopen-2016-015322.

44. Gibson OR, Segal L, McDermott RA. A systematic review of evidence on the association between hospitalisation for chronic disease related ambulatory care sensitive conditions and primary health care resourcing. *BMC Health Serv Res*. 2013 Aug 26;13:336. doi: 10.1186/1472-6963-13-336.

45. Corbella X, Ortega B, Juan A, et al. Alternatives to conventional hospitalization for improving lack of access to inpatient beds: A 12-year cross-sectional analysis. *J Hosp Administ*. 2013;2:9-21. doi: https://doi.org/10.5430/jha.v2n2p9.

46. Behrndt EM, Straubmeier M, Seidl H, et al. The German day-care study: multicomponent non-drug therapy for people with cognitive impairment in day-care centres supplemented with caregiver counselling (DeTaMAKS) – study protocol of a cluster-randomised controlled trial. *BMC Health Serv*. Res. 2017; 17: 492. doi: [10.1186/s12913-017-2422-x].

47. Fedotkina SA, Karajlanov MG, Rusev IT. Rational use of hospital-replacing technologies and forms of medical care. *Vestnik Sankt Peterburgskogo*

gosudarstvennogo universiteta. Medicina. 2017; 12 (2): 179-189; doi: 10.21638/11701/spbu11.2017.207.

48. Miani C, Ball S, Pitchforth E, et al. Organisational interventions to reduce length of stay in hospital: a rapid evidence assessment. *Health Services and Delivery Research* 2014;2:1–178.

49. Huckfeldt PJ, Kane RL, Yang Z, et al. Degree of Implementation of the Interventions to Reduce Acute Care Transfers (INTERACT) Quality Improvement Program Associated with Number of Hospitalizations. *J Am Geriatr Soc*. 2018 Sep;66(9):1830-1837. doi: 10.1111/jgs.15476. Epub 2018 Aug 10.

For peer review only

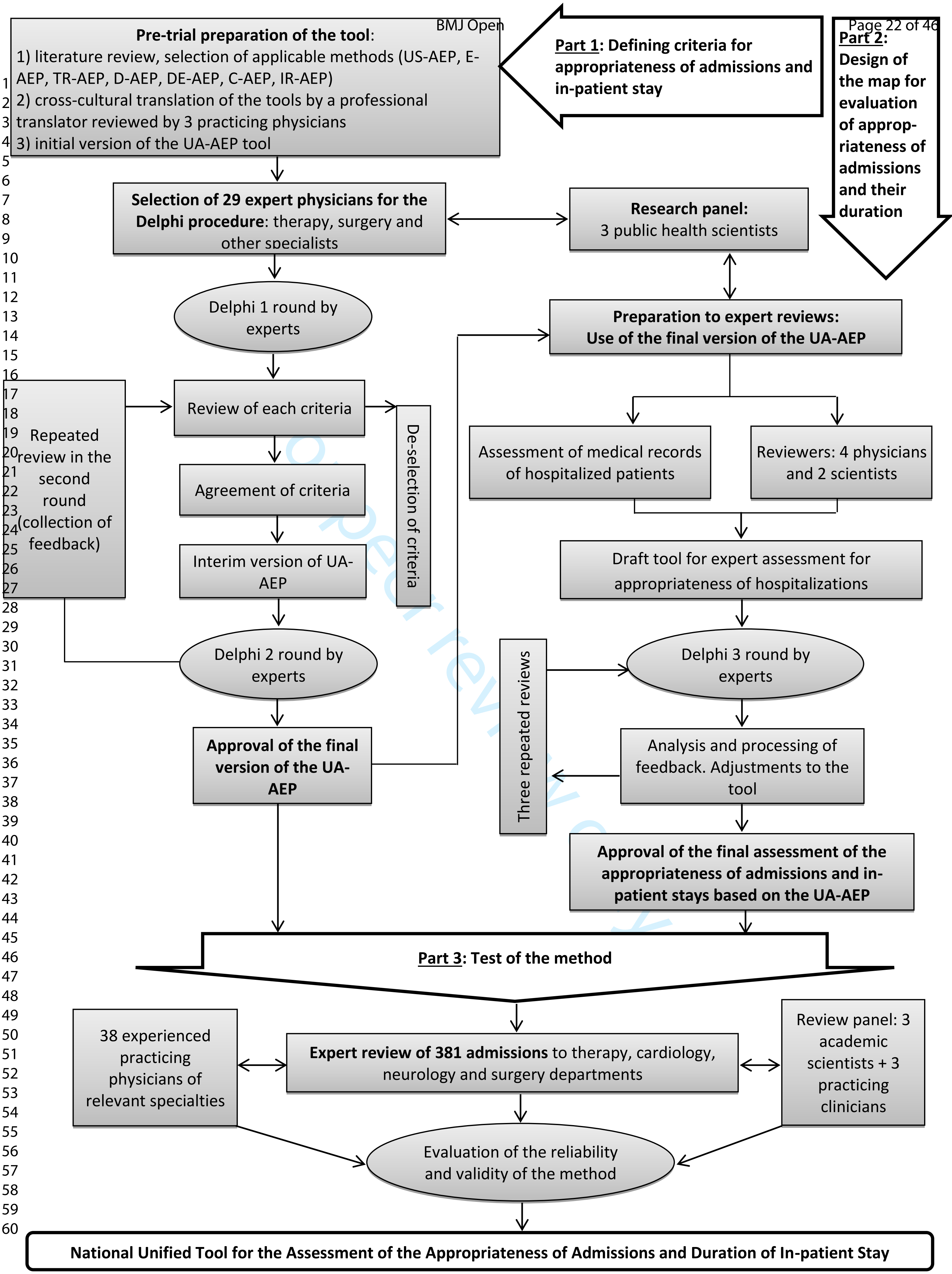

Figure 1 Schematic presentation of the steps followed during the development and evaluation of the method

Notes:

AEP –The Appropriateness Evaluation Protocol

US-AEP – American version [28]

E-AEP – European version [29]

TR-AEP – Turkish version [32]

D-AEP – Danish version [31]

DE-AEP – German version [33]

C-AEP – Chinese version [8]

DE-AEP – Iranian version [7]

For peer review only

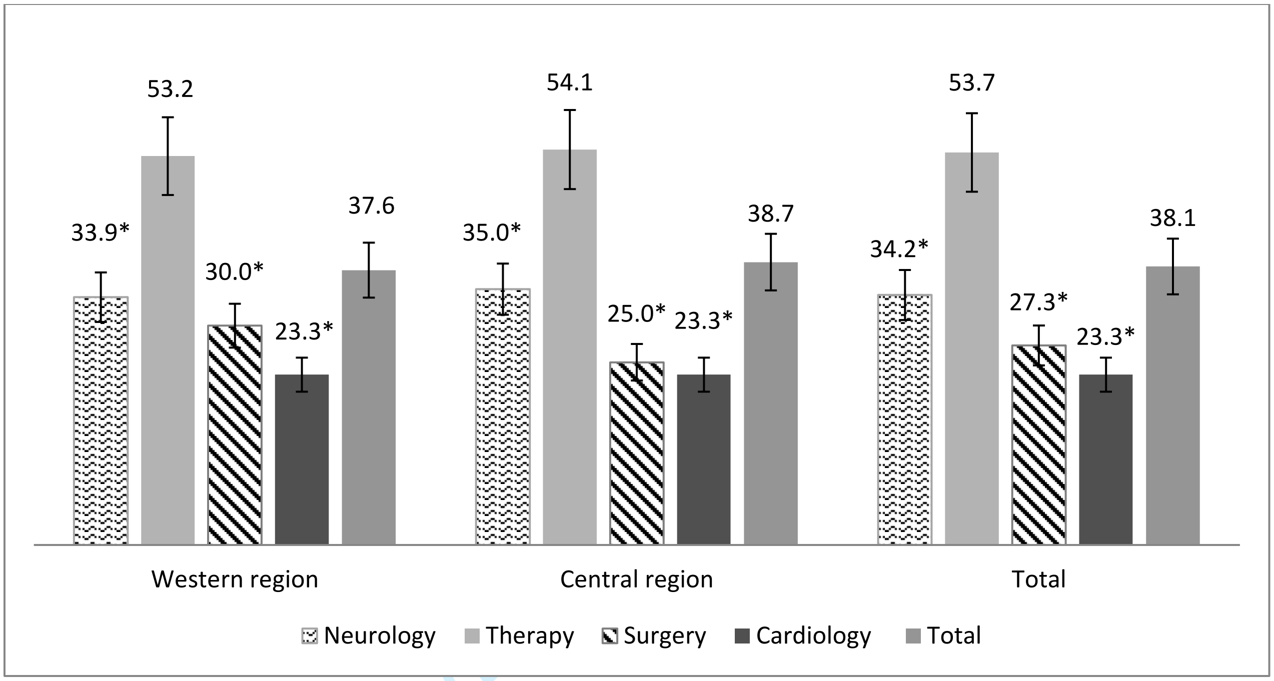

Figure 2 Shares of inappropriate admissions by department specialties, total and in the two regions of Ukraine (%, 95% CI)

Note: * - p<0.001 compared with the therapy department

For peer review only - http://bmjopen.bmj.com/site/about/guidelines.xhtml

Supplementary file 1

CRITERIA FOR APPROPRIATENESS OF HOSPITAL ADMISSIONS AND LENGTH OF INPATIENT STAY

Admission criteria

A. Severity of illness

1. Sudden loss of consciousness or disorientation (coma or unresponsiveness)
2. Acute confusional state, but not because of alcohol or narcotic intoxication
3. Acute loss of sight or hearing
4. Acute loss of ability to move part of the body
5. Acute ataxia (except for repeated paroxysms of acute ataxia in case of period/ episodic ataxias)
6. Persistent fever >38.0 C for 5 days without established diagnosis
7. Active bleeding which can lead to circulatory violations in case of absent homeostasis
8. Progressing acute neurological disorders
9. Acute or progressive sensory, motor, circulatory or respiratory disorder that can incapacitate the patient (inability to move, feed, breathe, urinate, etc.)
10. Unbearable pain (that cannot be managed by outpatient services (including primary or specialized outpatient or emergency service)
11. Acute abdominal pain with laboratory, ultrasound, X-ray disturbances lasting more than 6 hours
12. Severe pain, sudden edema, paling with cold peripheral limbs and decreased distal pulse
13. Inability to empty bowels or intestine movements (during the previous 24 hours), which cannot be attributed to the main neurological disorder
14. Patient's potentially life-threatening condition - for supervision (head injury, etc.)
15. Severe electrolyte/blood gas /CBC abnormality (any of the following, in mmol/L):
    - 123 < Na > 156
    - 2,5 < K > 6,0
    - 20 < HCO_3 > 36
    - 7.30 < Arterial blood pH > 7.45
    - 20 < CO_2 > 36 (unless chronically abnormal)
    - BUN (blood urea nitrogen) > 15 mg/dL (with increasing dynamics)
    - 3.9 ≤ FBS (Fasting blood sugar) ≥ 22.4 (associated with symptoms of weight reduction for no apparent reasons, polyuria, and polydipsia)
16. ECG evidence of acute ischemia (suspicion of a new myocardial infarction).
17. Acute heart rate disturbances (including ventricular fibrillation) and / or circulation with signs of hemodynamic disorders
18. Signs or symptoms of acute hematological disorders.

B. Intensity of Medical Services

1. Surgical or another procedure requiring equipment or other technologies available only in an inpatient facility (pre-surgery preparation, long – for more than 24 hours – clinical monitoring or supervision)
2. Invasive diagnostic procedures on the central nervous system.
3. Treatment requiring frequent dose adjustments in a potentially severe condition under direct medical supervision

C. Medical Care / Life Support Services

1. Intermittent or continuous use of an artificial lung ventilation at least 3 times per 24 hours.
2. Parenteral therapy - intermittent (2 or more times per day) or continuous intravenous fluid administration with any component / intravenous infusion of medication and / or fluid substitution (does not include tube nutrition).
3. Continuous monitoring of vital signs – at least every 30 mins, for at least 4 hours

For peer review only

D. Other admission criteria

1. Indications for hospitalization defined in the national unified clinical protocols (for specific diseases)
2. Advanced hospitalization with sufficiently documented justification in case of a threat of potential deterioration of the patient's condition (e.g., pregnant women with a high perinatal risk during the perinatal period etc.)
3. The need for isolation in case of an epidemiological or social threat (e.g., from the side of patients with psychological disorders etc.)
4. Other

Note. Hospitalization is not justified for the following cases:

- Provision of care is possible on an outpatient basis or using alternative to inpatient services (day care) irrespective of the remoteness of patient's place of living to the care facility (organization of boarding care for these patients if necessary);
- The patient mostly requires social care (lonely people etc.);
- The decision on hospitalization is made solely based on the referral from the primary care doctor or a specialist doctor;
- The need for hospitalization is caused by rigid rules of the Soviet period (for issuance of a disability status, for the conclusion on ability for military service etc.) and is not associated with the real condition of the patient and intensity of care required.

Duration of inpatient stay

Durations of inpatient stay are defined based on case-specific assessment of the dynamics of a patient condition.

Note. Inpatient stay is considered unjustified in the following cases:

- If a diagnostic test of the patient may be performed without or before patient's hospitalization;
- When based on his/her condition, the patient can continue treatment on a day-care or outpatient basis (irrespective of the remoteness of the patient's place of living from the inpatient facility – boarding care can be organized for such patients if necessary);
- When stay in an inpatient facility is caused by the patient's social problems (lonely people, those who have unsatisfactory living conditions, etc.).

For peer review only

Supplementary file 2

Assessment tool for appropriateness of hospitalization and hospital days

CARD FOR EXPERT ASSESSMENT OF APPROPRIATENESS OF ADMISSION AND DURATION OF INPATIENT STAY

The expert assessment uses data of medical record of the inpatient (form 003/o), health care facility passport or audit of secondary (specialist) care (Order of the MoH of Ukraine no. 637 dd. 17.08.2012 "On Approval of the Guidelines on the Procedure for Audit of Secondary (Specialist) Health Care", and other available data, criteria of reasonability of the admission.

City/town/village ____

Hospital ____

Department____

Inpatient's medical record no._______________ **Expert assessment date** _______ ______ ______

Full name of the expert____

I. GENERAL DATA AND CONDITION AT THE TIME OF INPATIENT ADMISSION

1. Age: _________ **complete years** **2. Sex:** 1 – male; 2 – female

3. Residence: 1 – rural 2 – urban

4. Living: 1. With a family (specify the number of people, if possible) ______
2. Alone
3. Difficult to tell

5. Clinical diagnosis at the time of admission:

____

6. Diagnosis at discharge (specify the name and ICD code)

Principal ____ICD_______

Complication of the principal disease ____ICD_______

____

Complication of treatment (incl. post-operational) ____ICD_______

____

Secondary diseases that influenced the course of the principal disease ____

____ICD_____

7. Inpatient admission:

1. Planned
2. Urgent - ________hours after the start of the disease or injury

8. This year, in relation to this disease, the inpatient:

1. Is admitted for the first time
2. Has been admitted ________ times

9. Reference provided by:

1 – primary care specialist
2 – outpatient facility specialist *(indicate specialization)* ______________________________
3 – ambulance unit
4 – sought medical attention herself/himself
5 – inpatient facility______________________________ *unit specialization* ______________________________
6 - other *(specify)*______________________________

10. Overall condition at the time of admission:

1 – satisfactory
2 – moderate severity
3 – severe
4 – critical

II. REASONABILITY OF ADMISSION

11. The admission is justified according to criteria of referring to admission

1. Yes, it is reasonable for this inpatient facility
2. Yes, for a higher-level inpatient facility (such as another secondary care facility for intensive care, or a tertiary care facility)
3. Yes, for another secondary inpatient facility for elective care
4. Unreasonable
5. Difficult to tell

Please provide any comments you have here ____

____

For peer review only

12. What justifies the appropriateness of admission (multiple choice allowed)
1. Intensity of the required and provided services
2. Severity of the case
3. Compliance with existing national unified clinical protocols
4. Other (specify) ____

13. If the admission is not justified, or if it is justified for an inpatient facility of a different level (see health care facility passport or audit materials), please specify the reasons (multiple choices allowed):
1. Overestimation of the severity of the case (incl. the threat of unexpected progress of the disease)
2. Inpatient admission to fill the beds of the facility
3. Premature referral (e.g., admission on Monday for a surgery planned for Wednesday)
4. There was no time or conditions for examination or consultation required to make a decision on admission
5. The admission was to reduce the cost of examination, e.g. admission of a patient living far from the hospital to avoid accommodation/hotel costs
6. Ignoring medical standards of indications to inpatient admission (usual, stereotypic practice)
7. A lower level inpatient care is impossible due to statutory funding fragmentation (for instances of inpatient admission to tertiary level facilities)
8. Admission at the request of a general practitioner (family doctor) or specialist doctor.
9. Admission at demand of the patient or his/her family
10. Admission is legally mandatory:
    - 10.1. To receive a medical conclusion on disability
    - 10.2. To receive a medical conclusion on fitness to military service
    - 10.3. Other (specify) ____
11. Social problems of the patient
    - 11.1. No opportunity to receive care at home (the patient is lonely, or other family members are disabled)
    - 11.2. Unfavorable conditions at home
    - 11.3. Other (specify) ____

14. What measures, in your opinion, could improve the appropriateness of admission (multiple choices allowed):
1. Availability of clear indications/criteria for admission to inpatient facilities of different levels
2. Creation of conditions for diagnostic examination in the outpatient setting or day patient facility
3. Removal of financial limitations concerning inpatient admission to a health care facility of the required level
4. Bringing facility's capacity (number of beds) in line with the need of certain type and intensity of care
5. Strict observance of existing unified clinical protocols
6. Creation of an opportunity to perform diagnostic examination in the facility in order to timely decide whether admission is required
7. Organization of a boarding house at the facility for people from remote locations
8. Elimination of linking the staff size to the number of beds
9. Clear delimitation of indications to medical and social care
10. Creation of conditions for provision of social care where indicated, incl. round the clock (development of social care facilities network)
11. Other (specify)____

III. JUSTIFICATION OF THE DURATION OF INPATIENT STAY

15. The patient required:
1. Intensive conservative treatment at this inpatient facility
2. Intensive conservative treatment at an inpatient facility of a higher level (e.g., he/she was receiving care at a secondary care facility while requiring tertiary care)
3. Intensive conservative treatment at an inpatient facility of a lower level (e.g., he/she was receiving care at a tertiary care facility while requiring secondary care)
4. Intensive surgical treatment at this inpatient facility
5. Intensive surgical treatment at an inpatient facility of a higher level (e.g., he/she was receiving care at a secondary care facility while requiring tertiary care)
6. Intensive surgical treatment at an inpatient facility of a lower level (e.g., he/she was receiving care at a tertiary care facility while requiring secondary care)
7. Elective care at this inpatient facility:
    - 7.1. With the permanent stay of doctor at the facility;
    - 7.2. Without the permanent stay of doctor at the facility

8. Elective care at a lower level inpatient facility
9. Social care:
   9.1. At home
   9.2. With temporary accommodation
   9.3. With permanent accommodation
10. No inpatient care
11. Other (specify) ____

IIIA. Diagnostic Stage of Inpatient Stay

16. Duration of diagnostic stage (the period from inpatient admission to the start of proper treatment) actual - final diagnosis:
as of the _________ day or ________ hour of stay at the facility

17. Duration of the diagnostic stage:
1. Reasonable 2. Unjustified 3. Difficult to estimate

18. The diagnosis determined at the inpatient facility was:
1. Correct
2. Incorrect (incorrect disease entity/not all the diseases determined)
3. Inaccurate (incorrect form/stage/extent/severity/clinical manifestations of the disease)
4. Difficult to estimate

IIIB. Treatment Stage of Inpatient Stay

19. Proper treatment (after determination of the final diagnosis) started:
on the ________ day or ________ hour of stay at the inpatient facility

20. Duration of the treatment period (from the final diagnosis to discharge)
actual - __________ days

21. Surgical interventions*:*
1. None performed 2. Performed (specify)
____Surgery code___________

22. Resuscitation/intensive care
1. None 2. Yes

23. Duration of the treatment stage:
1.Reasonable 2. Unjustified 3. Difficult to estimate

24. <u>If the duration of the inpatient stay was unjustified, name the reasons (multiple choices are allowed)</u>:

<u>24.1. General</u>
1. Premature admission
2. Trying to keep excessive capacity of the inpatient facility
3. Inadequate or absent unified clinical protocols
4. Absent or formalistic local protocols
5. Ignoring medical standards (conservative practice)
6. Admission to a facility where patient's problems could not be adequately resolved (e.g., the patient required intensive care, but the facility did not have the equipment for it)
7. Lack of equipments and materials at the facility: no opportunity for using modern treatment and diagnostic technology (such as MRI machine for diagnostics, laparoscopic equipment for certain surgical interventions, necessary supplies) – please specify____
8. Lack of medical personnel (specify) ____
9. Inefficient organization of specialist medical consultation
10. Lack of opportunity/unreasonably limited use of remote telemedical assistance
11. Irresolution (inability of making fast decisions, hesitation) of the patient and his/her family concerning diagnostic/treatment/surgical procedures
12. Patient's refusal from suggested treatment
13. Other (specify) ____

<u>24.2. Diagnostic stage-specific</u>
1. Unreasonable repetition of diagnostic laboratory and instrumental studies conducted at the previous stage of care, in the inpatient care setting
2. Financial incentives to increase the scope and number of the studies
3. Irrational schedule of diagnostic units (laboratory etc.) (such as impossibility of conducting diagnostic tests round the clock)
4. Insufficient efficiency of interaction with other facilities required to establish a diagnosis
5. Other (specify)____

For peer review only

24.3. Treatment stage-specific

1. Absent/insufficient capacities in terms of life support services provision (anesthesiology and intensive care department)
2. Lack of practice/organizational incapacity of discharging patients after making a decision of unreasonability of inpatient admission based on a short observation
3. Necessity of moving/transporting the patient to another health care facility (reduction of the duration of inpatient stay)
4. Deficit (absence) of an adequate alternative level of care - no capacity or practice of use of day patient facilities for follow-up care
5. Complications of the case due to incorrect diagnosis
6. Complications of the case due to low quality of the treatment
7. Doctor's overcautiousness regarding the discharge time, incl. due to ingrained stereotypes (e.g., discharge after receiving a pathologist's report etc.), please specify
   ____
8. Inefficient planning of the discharge (e.g., no discharging on week-ends)
9. Premature discharge in order to improve the performance indicators of the facility because of a high probability of death of the patient (reduction of the inpatient stay term)
10. The patient residing in another locality stayed at the facility because he/she was unable to get home on the day of discharge
11. Social problems of the patient:
    11.1. No opportunity to receive care at home (the patient is lonely, or other family members are disabled)
    11.2. Unfavorable conditions at home
12. Other (specify) ____
    ____

25. Estimates of the duration of inpatient stay

Treatment stage	*Actual bed-days*	*Recommended bed-days*
**I. Inpatient diagnostic**		
**II. Inpatient treatment**		
**Total**		

Please provide any comments you have ____
____

26. What measures, in your opinion, could improve the appropriateness of inpatient admission and stay (multiple choices allowed):

1. Bringing anesthesiology and intensive care departments of inpatient facilities intended for intensive care in line with the needs in terms of capacity, equipment and staffing
2. Bringing capacities of the facility (number of beds) in line with the needs of the specific type and intensity of care
3. Creation of conditions for diagnostics and follow-up care in day patient care or outpatient setting
4. Organization of a boarding house at the facility for people from remote locations requiring follow-up care at a day hospital or in the outpatient setting
5. Elimination of financial limitations preventing admission to an inpatient facility of the adequate level
6. Setting up wards for short-term stay of patients in emergency health care department/admission and examination department
7. Strict observation of existing unified clinical protocols
8. Clear criteria for discharge from inpatient facilities of different levels
9. Materials and equipment
10. Elimination of linking the staff size to the number of beds
11. Clear delimitation of indications to medical (including rehabilitation, hospice) and social care
12. Creation of conditions for provision of social care where indicated, incl. round the clock (development of social care facilities network)
13. Other (specify) ____
____

Your comments: __________
____
____

Signature of the Expert ___________________________

BMJ Open

Supplementary table 1

Avoidable hospitalizations and hospital days (results of the pilot)

Department	Facility	Patients	Bed-days	Avoidable hospitalizations		Admission to a wrong department/facility		Excessive inpatient stay		Excessive inpatient stay at wrong departments/facilities	
		Number	Number	Number/%	95% CI	Number/ %	95% CI	Number/% of bed-days	95% CI	Number/% of bed-days	95% CI
Therapy	Facility 1	40	426	29/72.5%	58.7% to 86.3%	2/5.0%	-1.8% to 11.8%	327/76.8%	72.7% to 80.8%	12/2.8%	1.2% to 4.4%
	Facility 2	30	297	12/40.0%	22.5% to 57.5%	3/10.0%	-0.7% to 20.7%	120/40.4%	34.8% to 46%	21/7.1%	4.2% to 10%
	Facility 3	30	311	13/43.3%	25.6% to 61.1%	1/3.3%	-3.1% to 9.8%	170/54.7%	49.1% to 60.2%	12/3.9%	1.7% to 6%
	**Total**	**100**	**1034**	**54/54.0%**	**44.2% to 63.8%**	**6/6.0%**	**1.3% to 10.7%**	**617/59.7%**	**56.7% to 62.7%**	**45/4.4%**	**3.1% to 5.6%**
Cardiology	Facility 1	41	537	10/24.4%	11.2% to 37.5%	-	-	231/43.0%	38.8% to 47.2%	-	-
	Facility 3	29	249	6/20.7%	5.9% to 35.4%	-	-	70/28.1%	22.5% to 33.7%	-	-
	**Total**	**70**	**786**	**16/22.9%**	**13.0% to 32.7%**	-	-	**301/38.3%**	**34.9% to 41.7%**	-	-
Neurology	Facility 1	30	306	2/6.7%	-2.3% to 15.6%	-	-	97/31.7%	26.5% to 36.9%	-	-
	Facility 2	59	645	14/23.7%	12.9% to 34.6%	-	-	253/39.2%	35.5% to 43%	-	-
	Facility 3	30	301	11/36.7%	19.4% to 53.9%	3/10.0%	-0.7% to 20.7%	149/49.5%	43.9% to 55.1%	25/8.3%	5.2% to 11.4%
	**Total**	**119**	**1252**	**27/22.7%**	**15.2% to 30.2%**	**3/2.5%**	**-0.3% to 5.3%**	**499/39.9%**	**37.1% to 42.6%**	**25/2.0%**	**1.2% to 2.8%**
Surgery	Facility 1	41	465	5/12.2%	2.2% to 22.2%	-	-	244/ 52.5%	47.9% to 57%	-	-
	Facility 2	30	181	10/33.3%	16.5% to 50.2%	2/6.7%	-2.3% to 15.6%	40/22.1%	16.1% to 28.1%	10/5.5%	2.2% to 8.9%
	Facility 3	21	144	5/23.8%	5.6% to 42.0%	5/23.8%	5.6% to 42.0%	45/31.3%	23.7% to 38.8%	27/18.8%	12.4% to 25.1%
	**Total**	**92**	**790**	**20/21.7%**	**13.3% to 30.2%**	**7/7.6%**	**2.2% to 13.0%**	**329/41.6%**	**38.2% to 45.1%**	**37/4.7%**	**3.2% to 6.2%**
**Total**		**381**	**3862**	**117/30.7%**	**26.1% to 35.3%**	**16/4.2**	**2.2% to 6.2%**	**1746/45.2%**	**43.6% to 46.8%**	**107/2.8%**	**2.3% to 3.3%**

For peer review only

Supplementary table 2

Description of the medical records included in the study

Region / *Level* / *Profile*	**Western region**			**Central region**			**Total for 2 regions**		
	Secondary care	Tertiary care	Both levels	Secondary care	Tertiary care	Both levels	Secondary care	Tertiary care	Both levels
Neurology	161	10	171	40	20	60	201	30	231
Therapy	71	40	111	120	50	170	191	90	281
Surgery	40	50	90	80	28	108	120	78	198
Cardiology	20	10	30	20	40	60	40	50	90
Total	292	110	402	260	138	398	552	248	800

For peer review only

Supplementary table 3

The possible reasons of unjustified hospital admissions and inpatient stays (expert opinions)

Reason	Frequency, % (95% CI)
An attempt to keep the current capacities in the inpatient facility	37.6 (30.5 to 44.6)
Hospital admission is defined by the need to fill inpatient beds and improve their occupancy	22.7 (16.6 to 28.7)
Absence of alternative to inpatient types of care or capacities to treat patients after their discharge	21.0 (15.1 to 26.9)
Absence of organizational possibilities to discharge patients after a decision is made that it is unjustified to keep them on a bed	9.9 (5.6 to 14.3)
Lack of timely examinations or consultations for necessary treatment	12.7 (7.8 to 17.6)
Inefficient cooperation with other professionals and facilities that need to be involved to establish a diagnosis	6.6 (3.0 to 10.3)
Non-adherence to clinical protocols or their outdated provisions	7.2 (3.4 to 10.9)
Overestimated severity of patient's condition	20.4 (14.6 to 26.3)

For peer review only

Supplementary table 4

Key proposed actions to prevent unjustified hospital admissions or unnecessary inpatient days

Actions	Frequency of suggesting by experts, % (95% CI)
To use clear indications or criteria for admission to inpatient facilities of different levels	32.6 (25.8 to 39.4)
To create conditions for diagnostic examinations and post-discharge care [follow-up] in outpatient facilities or on a day-care basis	44.8 (37.5 to 52.0)
To stop using personnel employment normative based on a facility or department's beds capacity	24.3 (18.1 to 30.6)
To bring the network and capacities of inpatient care facilities in compliance with the actual needs in a specific type and intensity of care	14.9 (9.7 to 20.1)
To arrange accommodation/ boarding facilities for persons living far from a treatment facility	33.7 (26.8 to 40.6)
To use clear criteria for patients' discharge from hospitals of different levels	19.9 (14.1 to 25.7)

For peer review only

Title and Abstract

1. Title	Assessment of avoidable hospitalizations in Ukraine: analytical framework, method, and findings (page 1)
2. Abstract	Objectives: This article reviews the magnitude of avoidable hospitalizations in two regions of Ukraine based on a customized version of the Appropriateness Evaluation Protocol (AEP).<br><br>Data and methods: We randomly selected 381 medical records to test the feasibility and reliability of the method and 800 medical records to evaluate the scope of unnecessary hospitalizations. A customized tool was developed following the Delphi procedure, which included criteria for the appropriateness of hospitalization and duration of inpatient stay. We used descriptive and analytical statistics, ROC analysis, and Cohen's kappa to check the consistency between the findings of primary reviewers and experts.<br><br>Result: We observed a high level of agreement in conclusions of primary reviewers (reference standard) and experts during testing of the reliability and validity of the method. The external validity check showed that the use of the tool by different experts provided high accuracy: 95.1 sensitivity, 76.6 specificity, and AUC=0.948 ($p<0.001$) for analysis of justifications of admissions; 95.3 sensitivity, 84.7 specificity, and AUC=0.900 ($p=0.001$) for the duration of hospitalizations. Cohen's kappa coefficient (κ) indicated agreement in expert evaluations of 0.915 (95 % CI 0.799 to 1.000) and 0.812 (95% CI 0.749 to 0.875), respectively.<br><br>We found that over one-third of admissions (38.1 %; 95 % CI 33.9 to 43.5) and over half of total bed-days were unnecessary (57.4 %; 95 % CI 56.4 to 58.5). The highest levels of stay were observed in hospitals' therapy departments (64.6%; 95 % CI 63.0 to 66.3); other departments included in the analysis – neurology, surgery, and cardiology – had significantly lower ($p<0.05$) levels.<br><br>Conclusion. The proposed method is robust in assessing the appropriateness of hospitalizations and durations of inpatient stays. The quantified levels of unnecessary hospital care indicate the need for improving efficiency and quality of care and optimizing the excessive hospital capacities in Ukraine. (page 2)

Introduction	*Why did you start?*
3. Problem Description	Hospital care is a core part of any national health system; it influences health outcomes of the population and requires significant funding. [1, 2] Still, use of hospital care has distinct features across the globe and ranges in terms of level of effectiveness. [3] If inpatient care fails to improve the health outcome when compared with less invasive or less intensive non-hospital care, it is considered an overuse of inpatient services. [4, 5, 6]<br><br>Overuse of hospital care is typical for many countries around the world, ranging from 1% to 54% in general [5], and up to 90% for some diseases. [7] Unnecessary hospitalizations are a matter of concern for many nations, specifically due to the rising trend of healthcare expenditures and a significant proportion of hospital expenditures in overall health spending. [8 - 12]<br><br>Hospital sector expenditures are mostly dependent on admission processes, length of patient hospital stays, and advancement of medical technologies in hospital facilities. Inpatient length of stay (LOS) is the most important contributing factor influencing the use of resources and cost of care during hospitalization; it closely relates to the effectiveness of hospital treatment, the effectiveness of hospital management, and general efficiency of a health system. [13, 14, 15] (page 4)
4. Available Knowledge	Rationalization of hospital expenditures together with increased, or at least sustained, quality of hospital care are key objectives of health care reform in Ukraine. [15] The current trends of poor health outcomes and suboptimal allocation of resources explain the importance of this objective. Ukraine is a middle-income country with $2,639.82 GDP per capita (2017) located in the Eastern Europe. It is behind other middle-income countries in terms of health sector performance. The average life expectancy at birth in 2017 was 72 years in Ukraine, 9 years less than in countries of the European Union (EU) and Organization for Economic Co-operation and Development (OECD). [17]<br><br>The hospital sector in Ukraine is oversized and inefficient by both the numbers of beds and hospitals. The number of beds per 10,000 people was 74.3 in 2016, or three times higher than in the EU; the number of hospitals per 10,000 people exceeded EU numbers by a quarter; the average length of stay was 11.2 days, or 3 days longer than for patients in the EU. [18]

	Hospital expenditures are relatively high. Hospitals are responsible for about one-third of total health expenditures (34.2% in 2016), [17] which is slightly lower than the OECD average (38% in 2015). [3] Between 82% to 92.7% [19, 20] of patients, however, pay out-of-pocket for medications needed during hospital treatment. If these costs are included, the share of hospital expenditures rises to over 50%. Until just recently, hospital budgets followed a historical Semashko system in which hospital funding was dependent on the number of beds. This budgetary system produced perverse incentives to preserve beds and maintain high bed occupancy rates, regardless of actual need for hospital care, and resulted in cost inefficiencies. [16]<br><br>Improvement of hospital efficiency has been among the health policy priorities since Ukraine gained independence in 1991. A new hospital financing reform is planned for 2019-2020. Numerous studies have been dedicated to the use of hospital care, [21 - 27] and the research has shown significant inefficiencies in the hospital sector. Reported results, however, vary substantially: between 8.2 and 70.6% unjustified hospitalizations, and 26.4 - 86.5 % of unnecessary bed-days. [21 - 27] (page 4-5)
5. Rationale	Studies in other countries have reported similar problems with variability of results [7, 8, 14], which led to an international recommendation from researchers that a unified method for the assessment of reasons for hospitalizations and their duration should be used, [7, 8] specifically the Appropriateness Evaluation Protocol (AEP). [28, 29] The AEP has high validity and reliability compared with other methods for assessment of inappropriate hospitalizations [2, 30] and was customized for many countries. [7, 8, 31]<br><br>The AEP method has not been used in Ukraine, where assessments are traditionally based on expert evaluations providing retrospective analyses of medical records. Such analyses are typically designed by individual experts through methods that are not validated. [21 - 27] Although the provision of hospital care is guided by legislation and clinical protocols, [18] concrete and evidence-based criteria for hospitalizations are absent. (page 5)
6. Specific Aims	**The objective** of this study was to develop and test the customized Ukraine version of the unified method for assessment of the appropriateness of hospitalizations, including reasons for admissions to hospitals and duration of hospital stays, and to evaluate the scope of inappropriate hospitalization of adults in Ukraine using a sample of hospitals from two regions. (page 5)

Methods	What did you do?
7. Context	Based on available international tools [7, 8, 28 - 33] and existing approaches used in Ukraine, [21 - 27] the tool for this study was developed and customized for the Ukrainian context in order to assess appropriateness of hospitalizations and duration of inpatient stays. (page 6)
8. Intervention(s)	The tool included criteria for appropriateness of hospital admissions and a guide for expert evaluations of justified inpatient lengths of stay. The tool was finalized using the Delphi method for expert evaluations [34] in three rounds as schematically presented in figure 1. (page 6)
9. Study of the Intervention(s)	Criteria for hospital admissions were included in the customized tool based on an analysis of seven versions of the AEP: original US version (US-AEP), [28] European (E-AEP), [29] Turkish (TR-AEP), [32] Dutch (D-AEP), [31] German (DE-AEP), [33] Chinese (C-AEP) [8], and Iranian version of the AEP (IR-AEP). [7] The customized Ukrainian AEP tool defined Ukraine-specific indications for hospital admission and criteria for justification of inpatient length of stay – summarized in the Ukrainian Appropriateness Evaluation Protocol (UA-AEP) (see online supplementary file 1). (page 6)
10. Measures	We used criteria from these versions of the UA-AEP to develop a guide that helps extract medical information about hospital care episodes from medical records (MoH form 003/o) for evaluation of the appropriateness of admission decisions and justification of length of stays (see online supplementary file 2).<br><br>Piloting of the method<br><br>The proposed tool was piloted in three types of hospitals of different size in one of the largest regions in eastern Ukraine. Four department profiles were selected for the analysis: therapy, cardiology, neurology, and surgery.<br><br>Standard medical records were used for analysis (MoH approved Form 003/o). The two-stage approach was used to sample records for the year preceding the analysis: first, one month was randomly selected (March of 2016), second, from all archived records in March of 2016, 381 cases were randomly selected. All sampled records

were proportionally distributed for evaluation to 38 experts with relevant clinical backgrounds for the profile of sampled cases.

The results of the piloting confirmed the feasibility of the tool for data collection and analysis. The pilot data showed that about the one-third of all admissions included in the analysis were classified as avoidable. The length of stay in facilities was often excessive: 45.2% of all bed-days were avoidable (see online supplementary table 1). The yielded results corresponded with the data received by other research of avoidable hospitalization in the same region, [23] and this confirmed the feasibility of the proposed method.

Reliability and validity of the method

The results of the pilot were verified by a group of 3 primary reviewers (public health scientists) who made up a moderation group. Each primary reviewer was assisted by a scientist in the relevant clinical field (therapist, surgeon and neurologist), making up the total review panel of 6 experts.

We assessed the reliability and validity of the method by comparing the evaluation results provided by 38 experts with the conclusions of the review panel. The latter was used as a reference standard for the conclusions.

The level of matching between two groups (experts and review panel) was analyzed using the coefficient of the overall agreement and Cohen's kappa coefficient (κ). We used ROC analysis to calculate sensitivity and specificity of the method and area under ROC curve (AUC) to show classification capacity of the method to define appropriateness of admissions and their duration.

Statistical methods

The dataset and statistics were prepared using Microsoft Excel (Microsoft Office 2016 Professional Plus, Open License 67528927) and STATISTICA 6.1 software (StatSoftInc., serial No. AGAR909E415822FA). We used descriptive and analytical statistics for data analysis. [35] For relative values, 95.0% confidence intervals (95% CI) were calculated based on the corrected Wald method. The assessment of the validity in the differences in relative indices was carried out according to Pearson's Chi-square test (Chi-square test – $\chi 2$).

For the sample calculations, we used Cochran formula with p=0.95 (α=0.05) and t=1.96. The general number of hospital admissions N for the pilot sites was 755,350

	and the hospital admission rate 23.3%, making the calculation for the minimal powered sample of 275 admissions for the pilot (sampled 381). For the main stage of the analysis, the recommended sample of 322 records (α=0,05) or 895 records (α=0,03) was calculated using "Power analysis" in STATISTICA 6.1 software for the general number of admissions in Ukraine of 8,594,210, and the level of inappropriate hospitalizations of 29.9% calculated for the pilot (sampled 800).<br>To analyze agreement in experts' reviews, we used the concordance coefficient (W) and Cronbach's alpha (α). Approximation of coefficient values to 1 indicates an increase in agreement rates.<br>The inter-rater agreement in experts' reviews was analyzed using overall agreement coefficient and Cohen's kappa coefficient (κ), applying the following ranges: values of 0.61-0.80 were defined as good strength of agreement, values of 0.81 and higher as very good strength of agreement. [35]<br>ROC curve analysis was conducted to calculate operational characteristics and the area under ROC-curve to evaluate discriminative capacity of the method. The relationship between the area under the ROC curve and diagnostic accuracy was assessed using the following ranges: 0.7-0.8 – good; 0.8-0.9 – very good; 0.9 – 1.0 – excellent. [36]<br>The statistical significance was taken at the level of p <0.05 (5%) for all tests.<br>(page 6-8)
**11. Analysis**	The following statistics describe the experts' agreement on the correctness of the customized tool proposed for the assessment of the appropriateness of hospitalizations: the criteria for hospital admissions concordance between evaluators was W=0.96 (p<0.01), Cronbach's alpha, α=0.943, approached the maximum possible level for reliability of repeated measurements; on the guide for evaluation of appropriateness of admission decisions and justification of length of stay, W=0.98 (p<0.05), Cronbach's alpha, α=0,926. (page 6)
**12. Ethical Considerations**	Confidentiality of information from medical records was kept by protecting patient identifiers. Researchers received copies of paper-based records with patient identification fields crossed out or covered. Names of physicians and hospital names were replaced with ID codes for reviewers of medical records.

	All experts working with primary data signed non-disclosure of confidential data and obligations to follow ethics principles of medical research statements, which are endorsed by international agreements and the Ministry of Health of Ukraine. Compliance with bioethics principles and medical deontology was confirmed in the conclusion of the Committee on bioethics and medical deontology in the State Institution "Dnipropetrovsk Medical Academy of the MOH Ukraine" (Protocol No.6 of September 16, 2016). (page 5-6)
**Results**	***What did you find?***
13. Results	Results of Reliability and Validity tests<br><br>We conclude that the pilot of the method proved its reliability and validity for the assessment of the hospitalizations because sensitivity and specificity measures are high for the sample and within the selected medical specialties for both evaluation of the appropriateness of admissions and duration of inpatient stay (Table 1).<br><br>Using Cohen's kappa (κ), we observed a high level of agreement in experts' reviews. Results for agreement on the appropriateness of admissions was 0.915 (95% CI (0.799 to 1.000) and duration of inpatient stay was 0.812 (95% CI 0.749 to 0.875).<br><br>The proposed method has very high operational characteristics: the area under the ROC curve only slightly varies for different medical specialties and averages to 0.948 for the appropriateness of admissions and to 0.900 for the duration of stay, which defines the potential to properly distinguish different patterns (discriminative capacity) of the method as excellent.<br><br>Core sample<br><br>Admissions at inpatient departments of secondary and tertiary care facilities were studied in two regions of Ukraine (central and western). The following 12 healthcare facilities were included in the study: in the central region – 2 regional (tertiary) and 5 city and district (secondary) healthcare facilities; in the western region – 1 regional (tertiary) and 4 city and district (secondary) healthcare facilities. The total number of discharged patients in the sampled facilities amounts to about 110,000 annually.<br><br>The expert assessment covered a random sample of 800 medical records in four medical specialties: therapy, neurology, surgery, and cardiology (Supplementary table 2). A similar approach was used to sample records for the core sample as in the pilot.

First, all medical records for patients discharged during the month preceding the beginning of data collection (July 2016) were collected from all departments of the therapy, surgery, neurology, and cardiology profile. Second, for each specialty department in each facility, the medical records were sampled randomly according to the discharge date. The total number of sample medical records made up 8% to 12% of the total number of patients discharged from the selected departments during that month.

The sampled medical records were proportionally distributed among experts according to their specialization. Experts reviewed medical records following the agreed guide for the analysis of the appropriateness of hospitalizations.

Results of data analysis for two regions of Ukraine

The findings of the expert review show that over one-third of cases – 38.1% (95% CI 33.9 to 43.5) in the central region, 38.7% (95% CI 34.8 to 41.5) in the western region and 37.6% (95% CI 32.8 to 42.3) in total were unjustified hospital admissions (figure 2). Every seventh patient (13.3%; 95%CI 6.5% to 20.8%) admitted to an inpatient department of tertiary care facilities could have been more appropriately treated at secondary care facilities with adequate equipping and staffing.

In our sample, over half of the total bed days spent by patients in hospitals were classified as avoidable/longer than necessary (57.4%). The volume of unjustified bed-days in the western region were far greater (63.1%) than in the central region (51.7%, $p<0.001$), which was related to a higher rate of unjustified admissions at secondary care facilities of patients to all specialized departments except the neurology profile (table 2).

The analysis of appropriateness of hospital admissions by specialties showed that the highest level of unjustified admissions was recorded for patients treated in therapy departments (53.7%); lower levels ($p<0.001$) were identified for all the other specialties: neurology – 34.2%; surgery – 27.3%; and cardiology – 23.3%.

The highest share of unjustified stay was determined for patients of therapy departments (64.6%), and the lowest share in cardiology (51.2%); for the neurology and surgery departments these shares were also high at 54.6% and 52.4% of total bed-days respectively.

	The experts were asked to specify one of the proposed reasons for unjustified hospitalization in each medical record reviewed. The following statistics describe the major reasons of inappropriate hospital admissions and unjustified inpatient stays: to keep patients under care in the absence of other facilities with capacity to provide care to patients after discharge (21.0%; 95% CI 15.1 to 26.9), and to treat patients with overestimated severity of their condition (20.4%; 95% CI 14.6 to 26.3) (see Supplementary table 3 for all reasons). (page 8-11)
**Discussion**	***What does it mean?***
14. Summary	Our study generates two key results regarding hospital care in Ukraine. First, there is a need for accurate assessment regarding appropriate hospital admissions. To increase the objectivity of assessing the use of hospital resources, we suggest applying a unified method, customized for Ukraine, which gives reliable results for analyzing the appropriateness and duration of hospital admissions. Second, the reserves for optimization of the hospital sector in Ukraine are high, as evidenced by the volume of inappropriate hospitalizations in the two regions estimated using the proposed method. (page 11-12)
15. Interpretation	The results of our study point to a high volume of unnecessary hospitalizations and their unjustified duration in Ukraine. The number of unjustified bed-days for appropriately hospitalized patients exceeded the number of bed-days from inappropriate admissions to the hospital by 27.8% (95% CI 24.4 to 31.1). This means that appropriately hospitalized patients often stay in the hospital longer than necessary. This share is especially large for patients hospitalized in therapy departments – the numbers of unjustified bed-days for appropriately admitted patients exceeds the number of bed-days for inappropriately admitted patients by 37.0% (95% CI 33.7 to 40.2).<br><br>We asked experts engaged in the evaluations to propose financial, economic, and organizational measures to prevent inappropriate hospitalizations or excessively long in-patient treatment (Supplementary table 4). Close to a quarter of respondents (24.3%; 95% CI 18.1 to 30.6) proposed to eliminate the normative regulation specifying the number of staff based on the number of beds in the facility. This principle, inherited from the Soviet era, incentivized hospitals to maintain a large number of beds regardless of the actual needs for inpatient care. Although the issue

had been included on the reform agenda for several decades, [15] it was only addressed in 2016 when the Ministry of Health abolished the existing normative regulation. This change, however, has not yet resulted in significant optimization of hospital capacities.

Hospitals in Ukraine do not differentiate by the intensity of interventions. In most hospitals, both acute and chronic patients, as well as those who need nursing and social care, can stay in the same departments to receive medical care. Another proposed measure for improvement of hospital efficiency was to right size the hospital network to align it with actual need in hospital care and categorize capacity of hospitals by intensity of care (14.9% of respondents, 95% CI 9.7 to 20.1), as well as the use of clear indications for admission to inpatient departments for facilities of different levels (32.6%, 95% CI 25.8 to 39.4). Such measures were proposed at the preliminary stage of the health reforms (2010-2014) [15] but not yet implemented.

The most popular measure proposed by experts was the introduction of alternatives to inpatient care: the replacement of inpatient services with outpatient services provided at the primary care level and the organization of treatment services without overnight stay (44.8% of respondents, 95% CI 37.5 – 52.0). This suggestion is in line with the current global trend of changes in the health system organization. [37, 38] Currently, the avoidance of hospitalizations of patients with ambulatory care-sensitive conditions (ACSCs) is included as a special policy direction on the agenda of many countries. [25, 39, 40, 41] The rate of avoidable hospitalization can be seen as an indicator of the quality of primary health care. [42, 43] Larger capacity of primary care to treat ACSCs results in a significant reduction of the hospitalizations of patients with these conditions. [44] Day-care clinics and day-care centers, as well as socially appropriate and less expensive forms of medical care, are widely used in different countries as alternatives to hospital services. [3, 45, 46]

To increase the availability of alternative care for patients from remote areas, an organization of day care coupled with discharge to a boarding house facility has proven to be effective. [47]. In such facilities, a patient, after receiving the relevant service or intervention, is placed in a boarding house where a range of services are available, including capacity for the provision of necessary care in the case of complications. This approach was favored by 33.7% of respondents (95% CI 26.8 to 40.6) [47]

	Daycare facilities are also gaining popularity in Ukraine. In 2016, 31.6% more patients received medical care in daycare facilities compared with the total number of hospitalized patients, but the increase in the use of daycare facilities did not replace inpatient care. [15] According to large-scale international studies, the combination of incentives and special training of medical staff can help to improve use of daycare instead of hospitalizations. [48, 49]<br><br>An additional measure is the timely discharge of patients from hospitals (proposed by 19.9% of experts, 95% CI 14.1 to 25.7). The issue of appropriate discharge is not unique to Ukraine. European experts point out that there are obstacles to timely discharge in multiple countries. [1]<br><br>The current health reform prepared and launched by the Government and the Ministry of Health in 2018 focuses primarily on changing the payment mechanisms, which will influence changes in the hospital sector. At the same time, reforms also cover the primary health care and emergency services to ensure accessibility of services within the scope of limited available resources in the country. (page 12-14)
16. Limitations	There are several limitations that may limit the generalizability of the findings related to possible seasonal variations for hospital care statistics and selection of regions that may not be fully representative for the whole of Ukraine. To minimize and adjust for limitations, we compared the sample data from two regions with the data collected from an additional region during the pilot stage. We did not find any significant differences between the regional statistics: the level of inappropriate admissions in the pilot was 30.7% (95% CI 26.0 to 35.4), which is slightly lower than the total for the surveyed regions but could be explained by the inclusion of municipal hospitals in the pilot, which had lower levels of inappropriate hospitalizations in the main sample as well. We, therefore, assume that the facilities in our research are representative of inpatient facilities in Ukraine. (page 12)
17. Conclusions	Optimization of inpatient care by reducing unnecessary hospitalizations and excessive lengths of stay in hospitals, given the current economic situation, may become more than just an important direction in the development of the health care system to increase effectiveness. It can essentially help to sustain good progress in the improvement of health sector organization and health outcomes by adequately meeting the needs of the population in hospital care. Although the suggested transformations of the hospital sector may require additional funds for structural

For peer review only

	reorganization, training, and motivation of medical staff, these investments are likely to be offset by the large efficiency gains from hospital optimization. (page 14)
**Other Information**	
18. Funding	The study was supported by the World Bank (within Improving effectiveness in human development and social accountability Project financed by the Department for International Development). (page 15)

For peer review only

Assessment of appropriateness of hospitalizations in Ukraine: analytical framework, method, and findings

Journal:	*BMJ Open*
Manuscript ID	bmjopen-2019-030081.R1
Article Type:	Original research
Date Submitted by the Author:	17-Sep-2019
Complete List of Authors:	Zhao, Feng ; World Bank Group, HNP<br>Doroshenko, Olena; World Bank Group, HNP<br>Lekhan, Valery ; Dnipropetrovsk Medical Academy (DMA), Department of Social Medicine and Health Management<br>Kriachkova, Lilia; Dnipropetrovsk Medical Academy (DMA)<br>Goroshko, Alona; National Health Service of Ukraine, Head of the Department for Development of Benefits Package
**Primary Subject Heading**:	Health services research
Secondary Subject Heading:	Health policy, Medical management, Research methods
Keywords:	hospital admissions, avoidable hospitalizations, unjustified inpatient stay, Ukraine version appropriateness evaluation protocol (AEP), reliability and validity of the method

SCHOLARONE™
Manuscripts

Assessment of appropriateness of hospitalizations in Ukraine: analytical framework, method, and findings

Feng Zhao, Program Leader, World Bank, Washington, DC.

Olena Doroshenko (corresponding author), Health Specialist, World Bank,

1 Dniprovskiy Uzviz, Kyiv, Ukraine, inglecasket@gmail.com, +38044 490-66-71.

ORCID iD https://orcid.org/0000-0002-0741-2863

Valery N. Lekhan, Professor, Department of social medicine and health management, Dnipropetrovsk Medical Academy (DMA), Dnipro, Ukraine.

ORCID iD https://orcid.org/0000-0003-2953-3292

Lilia V. Kriachkova, PhD/MD, Professor, Dnipropetrovsk Medical Academy (DMA), Dnipro, Ukraine.

ORCID iD https://orcid.org/0000-0001-7635-2609

Alona Goroshko, Head of the Department for Development of Benefits Package, National Health Service of Ukraine.

Word count, excluding title page, abstract, references, figures and tables – 3689

Keywords: hospital admissions, appropriateness of hospitalizations, unnecessary inpatient stay, Ukraine version appropriateness evaluation protocol (AEP), reliability and validity of the method

For peer review only

ABSTRACT

Objectives: This article reviews the applicability of a customized version of the Appropriateness Evaluation Protocol (AEP) to evaluate the magnitude of inappropriate hospitalizations in two regions of Ukraine.

Data and methods: The original AEP was modified to develop a customized tool, which included criteria for the appropriateness of hospitalization and duration of inpatient stay. The customization of the tool followed the Delphi procedure. We randomly selected 381 medical records to test the feasibility and reliability of the method and 800 medical records to evaluate the scope of inappropriate hospitalizations. We used descriptive and analytical statistics, ROC analysis, and Cohen's kappa to check the consistency between the findings of primary reviewers and experts.

Result: We observed high levels of agreement in conclusions of primary reviewers (reference standard) and experts during testing of the reliability and validity of the method. The external validity check showed that the use of the tool by different experts provided high accuracy: 95.1 sensitivity, 76.6 specificity, and AUC=0.948 ($p<0.001$) for analysis of the appropriateness of admissions; 95.3 sensitivity, 84.7 specificity, and AUC=0.900 ($p=0.001$) for the duration of hospitalizations. Cohen's kappa coefficient (κ) indicated agreement in expert evaluations of 0.915 (95% CI 0.799 to 1.000) and 0.812 (95% CI 0.749 to 0.875), respectively.

We found that over one-third of admissions (38.1%; 95% CI 33.9 to 43.5) and over half of total bed-days were unnecessary (57.4%; 95% CI 56.4 to 58.5). The highest levels of stay were observed in hospitals' general medicine departments (64.6%; 95% CI 63.0 to 66.3) compared to other departments included in the analysis.

Conclusion: The proposed method is robust in assessing the appropriateness of hospitalizations and duration of inpatient stays. The quantified levels of unnecessary hospital care indicate the need for improving efficiency and quality of care and optimizing the excessive hospital capacities in Ukraine.

ARTICLE SUMMARY

Strengths and limitations of this study

- An objective evaluation of inpatient care is possible through the application of a unified method for the assessment of the appropriateness of hospital admissions and duration of hospitalizations, which is customized for Ukraine and is based on clear criteria for in-patient treatment.
- The received data on the scope of inappropriate hospitalizations and their excessive duration provide a reliable base to estimate the needs of Ukraine's population in hospital care, which is especially relevant for the current reform of the health sector in the country.
- We evaluated the appropriateness of hospitalizations but did not evaluate the actual need in hospital care in Ukraine, and the capacities of health care institutions to adequately address the needs.
- Because electronic medical records were not available, the quality of the assessment of paper-based medical records was compromised by the structure and legibility of hand-written text.
- The future use of the proposed method in practice may require additional training for evaluators; its sustainability will depend on the uptake of the method at the national and at facility levels.

For peer review only

INTRODUCTION

Hospital care is a core part of any national health system; it influences health outcomes of the population and requires significant funding. [1, 2] Still, the use of hospital care has distinct features across the globe and ranges in terms of the level of effectiveness. [3] If inpatient care fails to improve the health outcome when compared with less invasive or less intensive non-hospital care, it is considered an overuse of inpatient services. [4, 5, 6]

Overuse of hospital care is typical for many countries around the world, ranging from 1% to 54% in general [5], and up to 90% for some diseases. [7] Unnecessary hospitalizations are a matter of concern for many nations, specifically due to the rising trend of healthcare expenditures and a significant proportion of hospital expenditures in overall health spending. [8 - 12]

Hospital sector expenditures are mostly dependent on admission processes, length of patient hospital stays, and advancement of medical technologies in hospital facilities. Inpatient length of stay (LOS) is the most important contributing factor influencing the use of resources and cost of care during hospitalization; it closely relates to the effectiveness of hospital treatment, the effectiveness of hospital management, and general efficiency of a health system. [13, 14, 15]

Rationalization of hospital expenditures together with increased, or at least sustained, quality of hospital care are key objectives of health care reform in Ukraine. [15, 16] The hospital sector in Ukraine is oversized and inefficient by both the numbers of beds and hospitals. The number of beds per 10,000 people was 74.3 in 2016, or three times higher than in the EU; the number of hospitals per 10,000 people exceeded EU numbers by a quarter; the average length of stay was 11.2 days, or 3 days longer than for patients in the EU. [17]

Numerous studies published in Ukraine within the last 15 years have been dedicated to the use of hospital care, [18-24] and the research has shown significant inefficiencies in the hospital sector. Reported results, however, vary substantially: between 8.2 and 70.6% inappropriate hospitalizations, and 26.4 - 86.5% of unnecessary bed-days.

Studies in other countries have reported similar problems with the variability of results, which led to an international recommendation from researchers that a unified method for the assessment of reasons for hospitalizations and their duration should be used, [7, 8] specifically the Appropriateness Evaluation Protocol (AEP). [25, 26] The AEP has high

For peer review only

validity and reliability compared with other methods for assessment of inappropriate hospitalizations [2, 27] and was customized for many countries. [7, 8, 28]

The AEP method has not been used in Ukraine, where assessments are traditionally based on expert evaluations providing retrospective analyses of medical records. Such analyses are typically designed by individual experts through methods that are not validated. [18-24] Although the provision of hospital care is guided by legislation and clinical protocols, concrete and evidence-based criteria for hospitalizations are absent. Additionally, a more accurate estimate of the needs in hospital care is necessary to support the ongoing health reform in Ukraine, which is implemented improve access and quality to health care, and to address inefficiencies in service delivery.

The objective of this study was to develop and test the customized Ukraine version of the unified method for assessment of the appropriateness of hospitalizations, including reasons for admissions to hospitals and duration of hospital stays, and to evaluate the scope of inappropriate hospitalization of adults in Ukraine using a sample of hospitals from two regions.

METHODS

Ethics statement

Confidentiality of information from medical records was kept by protecting patient identifiers. Researchers received copies of paper-based records with patient identification fields crossed out or covered. Names of physicians and hospital names were replaced with ID codes for reviewers of medical records.

All experts working with primary data signed non-disclosure agreements of confidential data and obligations to follow ethics principles of medical research statements, which are endorsed by international agreements and the Ministry of Health of Ukraine. Compliance with bioethics principles and medical deontology was confirmed in the conclusion of the Committee on bioethics and medical deontology in the State Institution “Dnipropetrovsk Medical Academy of the MOH Ukraine” (Protocol No.6 of September 16, 2016).

Patient and Public Involvement

We did not involve patients or the public in our work.

Tool development

Based on available international tools [8, 9, 25-30] and existing approaches used in Ukraine, [18-24] the tool for this study was developed and customized for the Ukrainian context in order to assess the appropriateness of hospitalizations and duration of inpatient stays. The tool included criteria for appropriateness of hospital admissions and a guide for expert evaluations of justified inpatient lengths of stay. The tool was finalized using

For peer review only

the Delphi method for expert evaluations [31] in three rounds as schematically presented in figure 1. To avoid the potential conflict of interests, experts represented different health care facilities, provided informed consent, received only summarized information of inputs provided before each round, and did not communicate about the study before the final approval of the modifications to the AEP. The group of reviewers controlled the adherence to the Delphi procedure. Criteria for hospital admissions were included in the customized tool based on an analysis of seven versions of the AEP: original US version (US-AEP), [25] European (E-AEP), [26] Turkish (TR-AEP), [29] Dutch (D-AEP), [28] German (DE-AEP), [30] Chinese (C-AEP) [9], and Iranian version of the AEP (IR-AEP). [8] The customized Ukrainian AEP tool defined Ukraine-specific indications for hospital admission and criteria for the justification of inpatient length of stay – summarized in the Ukrainian Appropriateness Evaluation Protocol (UA-AEP) (see online supplementary file 1).

We used criteria from these versions of the UA-AEP to develop a guide that helps to extract medical information about hospital care episodes from medical records (MoH form 003/o) for evaluation of the appropriateness of admission decisions and justification of lengths of stay (see online supplementary file 2).

Piloting of the method

The proposed tool was piloted in three types of hospitals of different size in one of the largest regions in eastern Ukraine. Four department profiles were selected for the analysis: general medicine, cardiology, neurology, and surgery. These departments are representing a most typical structure of a hospital: on average, 44% of all adult patients are admitted to these departments in Ukraine.

Standard medical records were used for analysis (MoH approved Form 003/o). The two-stage approach was used to sample records for the year preceding the analysis: first, one month was randomly selected (March 2016); second, from all archived records in the four departments in March 2016, 381 cases were randomly selected. All sampled records were proportionally distributed for evaluation to 38 experts with relevant clinical backgrounds for the profile of sampled cases. Experts were selected based on their area of expertise and the results of the initial test of competencies. The total number of experts was determined based on an average anticipated workload of 10 medical records per one expert.

The results of the piloting confirmed the feasibility of the tool for data collection and analysis. The pilot data showed that about one-third of all admissions included in the analysis were classified as inappropriate. The length of stay in facilities was often

For peer review only - http://bmjopen.bmj.com/site/about/guidelines.xhtml

excessive: 45.2% of all bed-days were inappropriate (see online supplementary table 1). The yielded results corresponded with the finding from a study of inappropriate hospitalizations in the same region, [20] and this confirmed the feasibility of the proposed method.

Reliability and validity of the method

The results of the pilot were verified by a review panel, consisting of three primary reviewers (public health scientists). Each primary reviewer was assisted by a clinician in the relevant clinical field (therapist, surgeon, and neurologist).

We assessed the reliability and validity of the method by comparing the evaluation results provided by 38 experts with the conclusions of the review panel. The latter was used as a reference standard for the conclusions.

Statistical methods

The dataset and statistics were prepared using Microsoft Excel (Microsoft Office 2016 Professional Plus, Open License 67528927) and STATISTICA 6.1 software (StatSoftInc., serial No. AGAR909E415822FA). We used descriptive and analytical statistics for data analysis. [32] For relative values, 95.0% confidence intervals (95% CI) were calculated based on the corrected Wald method. The assessment of the validity in the differences in relative indices was carried out according to Pearson's Chi-square test (Chi-square test – χ^2).

For the sample calculations, we used Cochran formula with p=0.95 (α=0.05) and t=1.96. The general number of all hospital admissions N for catchment area served by the hospitals included in pilot sites was 755,350 and the hospital admission rate 23.3%, making the calculation for the minimal powered sample of 275 admissions for the pilot study (sampled 381). For the main stage of the analysis, the recommended sample of 322 records (α=0,05) or 895 records (α=0,03) was calculated using "Power analysis" in STATISTICA 6.1 software for the general number of admissions in Ukraine of 8,594,210, and the level of inappropriate hospitalizations of 29.9% calculated for the pilot (sampled 800).

To analyze agreement in experts' reviews, we used the concordance coefficient (W) and Cronbach's alpha (α). Approximation of coefficient values to 1 indicates an increase in agreement rates.

The inter-rater agreement in experts' reviews was analyzed using overall agreement coefficient and Cohen's kappa coefficient (κ), applying the following ranges: values of 0.61-0.80 were defined as good strength of agreement, values of 0.81 and higher as very good strength of agreement. [32]

For peer review only - http://bmjopen.bmj.com/site/about/guidelines.xhtml

ROC curve analysis was conducted to calculate operational characteristics and the area under ROC-curve (AUC) to evaluate the discriminative capacity of the method. The relationship between the area under the ROC curve and diagnostic accuracy was assessed using the following ranges: 0.7-0.8 – good; 0.8-0.9 – very good; 0.9 – 1.0 – excellent. [33]

The statistical significance was taken at the level of p <0.05 (5%) for all tests.

RESULTS

Results of Reliability and Validity tests

The Ukraine version of the AEP proved its reliability and validity for the assessment of the hospitalizations because sensitivity and specificity measures are high for the sample and within the selected medical specialties for evaluation of the appropriateness of admissions and duration of inpatient stay (Table 1).

Table 1

Inter-rater agreement of experts' evaluations and Operational Characteristics of the Method for Assessment of the Appropriateness of Hospitalizations

Statistics	General medicine n=100	Cardiology n=70	Neurology n=119	Surgery n=92	Total pilot sample n=381
Appropriateness of admissions					
Sensitivity (%)	84.8	96.3	96.4	97.3	95.1
Specificity (%)	73.1	87.5	68.5	94.7	76.6
Area under ROC curve (p-value)	0.865 (0.002)	0.977 (<0.001)	0.945 (<0.001)	0.991 (<0.001)	0.948 (<0.001)
Overall agreement (%)	84.0	94.2	88.2	90.5	92.8
Cohen's κ (95% CI)	0.702 (0.566 to 0.839)	0.916 (0.871 to 0.961)	0.888 (0.781 to 0.996)	0.934 (0.843 to 1.000)	0.915 (0.799 to 1.000)
Duration of inpatient stay					
Sensitivity (%)	88.4	96.3	96.6	97.2	95.3
Specificity (%)	85.9	87.5	75.0	94.4	84.7
Area under ROC curve (p-value)	0.870 (0.009)	0.920 (<0.001)	0.860 (0.007)	0.960 (<0.001)	0.900 (0.001)
Overall agreement (%)	86.7	91.9	90.1	94.3	91.2
Cohen's κ (95% CI)	0.737 (0.604 to 0.870)	0.838 (0.684 to 0.992)	0.772 (0.639 to 0.905)	0.902 (0.794 to 1.000)	0.812 (0.749 to 0.875)

Using Cohen's kappa (κ), we observed a high level of agreement in experts' reviews. Results for agreement on the appropriateness of admissions was 0.915 (95% CI (0.799

to 1.000) and duration of inpatient stay was 0.812 (95% CI 0.749 to 0.875).

The proposed method has very high operational characteristics: the area under the ROC curve only slightly varies for different medical specialties and averages to 0.948 for the appropriateness of admissions and to 0.900 for the duration of stay, which defines the potential to properly distinguish different patterns (discriminative capacity) of the method as excellent.

Core sample

Admissions at inpatient departments of secondary and tertiary care facilities were studied in two regions of Ukraine (central and western). The total of 12 healthcare facilities was included in the study, of which 9 facilities are secondary care hospitals, and 3 are tertiary care facilities.

The expert assessment covered a random sample of 800 medical records in four medical specialties: general medicine, neurology, surgery, and cardiology (Supplementary table 2). A similar approach was used to sample records for the core sample as in the pilot. All medical records for patients discharged during the month preceding the beginning of data collection (July 2016) were collected from all departments of the general medicine, surgery, neurology, and cardiology profile. The total number of sample medical records made up 8% to 12% of the total number of patients discharged from the selected departments during that month.

Experts, according to their specialization, reviewed medical records following the agreed guide for the analysis of the appropriateness of hospitalizations.

Results of data analysis for two regions of Ukraine

The findings of the expert review show that over one-third of cases – 38.1% (95% CI 33.9 to 43.5) in the central region, 38.7% (95% CI 34.8 to 41.5) in the western region and 37.6% (95% CI 32.8 to 42.3) in total were inappropriate hospital admissions (figure 2). Every seventh patient (13.3%; 95%CI 6.5% to 20.8%) admitted to an inpatient department of tertiary care facilities could have been more appropriately treated at secondary care facilities with adequate equipping and staffing.

In our sample, over half of the total bed days spent by patients in hospitals were classified as inappropriate or longer than necessary (57.4%). The volume of unnecessary bed-days in the western region was far greater (63.1%) than in the central region (51.7%, $p<0.001$), which was related to a higher rate of inappropriate admissions at secondary care facilities of patients to all specialized departments except the neurology profile (table 2).

Table 2

Volumes of inappropriate hospitalizations

Characteristic of the sample	Number of inappropriate admissions	Total bed-days for inappropriate hospitalizations	Average length of stay in bed-days (95% CI)	Unnecessary days of inpatient stay, % (95% CI)
**Total for 2 regions**				
By facility level				
Secondary care	217	2255	7.1 (6.9 to 7.2)	58.8 (57.5 to 60.1)
Tertiary care	88	1274	8.2 (7.8 to 8.5)	54.8 (52.9 to 56.6)#
Both levels	305	3530	7.4 (7.3 to 7.5)	57.4 (56.4 to 58.5)
By specialty of departments				
Neurology	79	1123	7.9 (7.7 to 8.0)	54.6 (52.6 to 56.5)°
General medicine	151	1083	8.8 (8.6 to 9.0)	64.6 (63.0 to 66.3)
Surgery	54	838	5.9 (5.7 to 6.0)	52.4 (50 to 54.7)°
Cardiology	21	486	7.3 (7.0 to 7.5)	51.2 (48.1 to 54.3)°
**Central region**				
By facility level				
Secondary care	105	1252	8.2 (8 to 8.4)	50.6 (48.7 to 52.6)*
Tertiary care	49	740	8.5 (8.3 to 8.7)	53.4 (51.0 to 55.9)
Both levels	154	1993	8.3 (8.2 to 8.4)	51.7 (50.1 to 53.2)*
By specialty of departments				
Neurology	79	1123	7.9 (7.7 to 8.0)	55.0 (51.2 to 58.8)
General medicine	151	1083	8.8 (8.6 to 9.0)	58.4 (56.1 to 60.7)*
Surgery	54	838	5.9 (5.7 – 6.0)	41.9 (38.9 to 44.9)*°
Cardiology	21	486	7.3 (7.0 to 7.5)	46.6 (42.9 to 50.2)*°
**Western region**				
By facility level				
Secondary care	112	1002	6.0 (5.7 to 6.3)	65.9 (64.2 to 67.6)
Tertiary care	39	533	7.7 (6.6 to 8.8)	56.6 (53.8 to 59.4)#
Both levels	151	1535	6.5 (6.3 to 6.7)	63.1 (61.7 to 64.6)
By specialty of departments				
Neurology	58	830	7.8 (7.5 to 8.2)	54.4 (52.1 to 56.7)°
General medicine	59	366	7.8 (7.3 to 8.3)	72.7 (70.3 to 75.1)
Surgery	27	237	3.8 (3.4 to 4.3)	67.4 (63.9 to 70.8)
Cardiology	7	102	4.9 (4.1 to 5.6)	63.0 (57.3 to 68.7)°

Notes: * - $p<0.001$ compared with the other region
# - $p\leq0.001$ compared with the secondary level facilities
° - $p<0.001$ compared with the general medicine departments

The analysis of appropriateness of hospital admissions by specialties showed that the highest level of inappropriate admissions was recorded for patients treated in general medicine departments (53.7%); lower levels ($p<0.001$) were identified for all the other specialties: neurology – 34.2%; surgery – 27.3%; and cardiology – 23.3%.

The highest share of unnecessary stay was determined for patients of general medicine departments (64.6%), and the lowest share in cardiology (51.2%); for the neurology and

For peer review only

surgery departments these shares were also high at 54.6% and 52.4% of total bed-days respectively.

The experts were asked to specify one of the proposed reasons for inappropriate hospitalization in each medical record reviewed. The following statistics describe the major reasons of inappropriate hospital admissions and unnecessary inpatient stays: to keep patients under care in the absence of other facilities with capacity to provide care to patients after discharge (21.0%; 95% CI 15.1 to 26.9), and to treat patients with overestimated severity of their condition (20.4%; 95% CI 14.6 to 26.3) (see Supplementary table 3 for all reasons).

DISCUSSION

Like in the other countries, [8-9, 28-30] we have customized the European version of the AEP to adapt it to Ukrainian specifics. The scope of changes is marginal: we have amended some criteria, included relevant criteria and modified the wording. Such changes of the AEP are explained by the lower technological intensity of health care provision in Ukraine and peculiarities of service delivery in the country. The proposed Ukrainian version of Appropriateness Evaluation Protocol (AEP-UA) is a reliable and valid tool for assessment of the appropriateness of hospitalizations for Ukraine's hospitals, as confirmed by the high levels of Cohen's kappa coefficient (κ) for agreement on criteria of appropriateness and duration of hospitalizations (0.915 and 0.812 respectively).

Our study generates two key results regarding hospital care in Ukraine. First, there is a need for accurate assessment of the appropriateness of hospital admissions. To increase the objectivity of assessing the use of hospital resources, we suggest applying a unified method, customized for Ukraine, which gives reliable results for analyzing the appropriateness and duration of hospital admissions. Second, the reserves for optimization of the hospital sector in Ukraine are high, as evidenced by the volume of inappropriate hospitalizations in the two regions estimated using the proposed method.

There are several limitations that may limit the generalizability of the findings related to possible seasonal variations for hospital care statistics and selection of regions that may not be fully representative for the whole of Ukraine. To minimize and adjust for limitations, we compared the sample data from two regions with the data collected from an additional region during the pilot stage. We did not find any significant differences between the regional statistics: the level of inappropriate admissions in the pilot was 30.7% (95% CI 26.0 to 35.4), which is slightly lower than the total for the surveyed regions but could be explained by the inclusion of municipal hospitals in the pilot, which had lower levels of

For peer review only

inappropriate hospitalizations in the main sample as well. We, therefore, assume that the facilities in our research are representative of inpatient facilities in Ukraine.

Among other limitations we report that we did not evaluate the actual need in hospital care in Ukraine, and the capacities of health care institutions to adequately address the needs. Because electronic medical records were not available, the quality of the assessment of paper-based medical records was compromised by the structure and legibility of hand-written text. We also suggest that the future use of the proposed method in practice may require additional training for evaluators; its sustainability will depend on uptake of the method at the national and at facility levels.

The results of our study point to a high volume of inappropriate hospitalizations and their unjustified duration in Ukraine. The number of unnecessary bed-days for appropriately hospitalized patients exceeded the number of bed-days from inappropriate admissions to the hospital by 27.8% (95% CI 24.4 to 31.1). This means that appropriately hospitalized patients often stay in the hospital longer than necessary. This share is especially large for patients hospitalized in general medicine departments – the numbers of unnecessary bed-days for appropriately admitted patients exceeds the number of bed-days for inappropriately admitted patients by 37.0% (95% CI 33.7 to 40.2).

We asked experts engaged in the evaluations to propose financial, economic, and organizational measures to prevent inappropriate hospitalizations or excessively long in-patient treatment (Supplementary table 4). Close to a quarter of respondents (24.3%; 95% CI 18.1 to 30.6) proposed to eliminate the normative regulation specifying the number of staff based on the number of beds in the facility. This principle inherited from the Soviet era, incentivized hospitals to maintain a large number of beds regardless of the actual needs for inpatient care. Although the issue had been included in the reform agenda for several decades, [15] it was only addressed in 2016 when the Ministry of Health abolished the existing normative regulation. This change, however, has not yet resulted in significant optimization of hospital capacities.

Hospitals in Ukraine do not differentiate by the intensity of interventions. In most hospitals, both acute and chronic patients, as well as those who need nursing and social care, can stay in the same departments to receive medical care. Another proposed measure for improvement of hospital efficiency was to right-size the hospital network to align it with actual need in hospital care and categorize capacity of hospitals by intensity of care (14.9% of respondents, 95% CI 9.7 to 20.1), as well as the use of clear indications for admission to inpatient departments for facilities of different levels (32.6%, 95% CI 25.8 to

For peer review only

39.4). Such measures were proposed at the preliminary stage of the health reforms (2010-2014) [15] but not yet implemented.

The most popular measure proposed by experts was the introduction of alternatives to inpatient care: the replacement of inpatient services with outpatient services provided at the primary care level and the organization of treatment services without overnight stay (44.8% of respondents, 95% CI 37.5 – 52.0). This suggestion is in line with the current global trend of changes in the health system organization. [34, 35] Currently, the avoidance of hospitalizations of patients with ambulatory care-sensitive conditions (ACSCs) is included as a special policy direction on the agenda of many countries. [36-38] The rate of the appropriateness of hospitalizations can be seen as an indicator of the quality of primary health care. [39, 40] Larger capacity of primary care to treat ACSCs results in a significant reduction of the hospitalizations of patients with these conditions. [41] Day-care clinics and day-care centers, as well as socially appropriate and less expensive forms of medical care, are widely used in different countries as alternatives to hospital services. [3, 42, 43]

To increase the availability of alternative care for patients from remote areas, an organization of day care coupled with discharge to a boarding house facility has proven to be effective. [44]. In such facilities, a patient, after receiving the relevant service or intervention, is placed in a boarding house where a range of services is available, with a capacity for the provision of necessary care in the case of complications. This approach was favored by 33.7% of respondents (95% CI 26.8 to 40.6) [44]

Daycare facilities are also gaining popularity in Ukraine. In 2016, 31.6% more patients received medical care in daycare facilities compared with the total number of hospitalized patients, but the increase in the use of daycare facilities did not replace inpatient care. [15] According to large-scale international studies, the combination of incentives and special training of medical staff can help to improve the use of daycare instead of hospitalizations. [45, 46]

An additional measure is the timely discharge of patients from hospitals (proposed by 19.9% of experts, 95% CI 14.1 to 25.7). The issue of appropriate discharge is not unique to Ukraine. European experts point out that there are obstacles to timely discharge in multiple countries. [1]

Interestingly, experts did not consider better coordination with social services as one of the factors to reduce inappropriate hospitalizations and duration of stay (only 5% respondents agreed with this statement, 95% CI 1,7 to 8,2), which can be explained by the low level of social service development and weak coordination between health and

For peer review only

social sectors in the country. At the same time, strong evidence supports the need to create an interface between health and social care providers, which can help reduce levels of hospitalizations and their duration for patients, especially elderly [47, 48].

The current health reform prepared and launched by the Government and the Ministry of Health in 2018 focuses primarily on changing the payment mechanisms, which will influence changes in the hospital sector. At the same time, reforms also cover the primary health care and emergency services to ensure accessibility of services within the scope of limited available resources in the country. The results received and future use of the proposed method for the assessment of the appropriateness of hospitalizations can inform decisions in hospital restructuring.

Optimization of inpatient care by reducing inappropriate hospitalizations and excessive lengths of stay in hospitals, given the current economic situation, may become more than just an important direction in the development of the health care system to increase effectiveness. It can essentially help to sustain good progress in the improvement of health sector organization and health outcomes by adequately meeting the needs of the population in hospital care. Although the suggested transformations of the hospital sector may require additional funds for structural reorganization, training, and motivation of medical staff, these investments are likely to be offset by the large efficiency gains from hospital optimization.

For peer review only

FOOTNOTES

Acknowledgments

The authors thank all staff working in Health Departments of the two regions who facilitated the process of data collection, assisted with the preparation of the primary dataset, and participated in the discussion for data interpretation.

Data statement

Extra data can be accessed via the Dryad data repository at http://datadryad.org/ with the doi: 10.5061/dryad.2v6wwpzh9

Authors' Contributions:

F. Zhao – Critical review of the article for important intellectual content,
O. Doroshenko - Drafting the article, preparing the first concept of the study,
V. Lekhan - Substantial contribution to the concept and design of the study, overall guidance and coordination of the study,
L. Kriachkova - Substantial contribution to the acquisition of data, analysis, and interpretation of data, and the first draft of the article,
A. Goroshko – Contributions to the first draft of the article.

Competing Interests

None declared

Funding and all other required statements

The study was supported by the World Bank (within Improving effectiveness in human development and social accountability Project financed by the Department for International Development).

Figures and supplementary files included in the manuscript

Figure 1 Schematic presentation of the steps followed during the development and evaluation of the method

Figure 2 Shares of inappropriate admissions by department type, total and in the two regions of Ukraine (%, 95% CI)

Supplementary file 1 Criteria for appropriateness of hospital admissions and length of inpatient stay

For peer review only

Supplementary file 2 Assessment tool for appropriateness of hospitalizations and hospital days

Supplementary table 1 Inappropriate hospitalizations and unnecessary hospital days (results of the pilot)

Supplementary table 2 Medical records included in the study

Supplementary table 3 The possible reasons for inappropriate hospital admissions and inpatient stays (expert opinions)

Supplementary table 4 Key proposed actions to prevent inappropriate hospital admissions or unnecessary inpatient days

For peer review only

REFERENCES

1. McKee M, Healy J. Hospitals in changing Europe. (European Observatory on Health Care Systems series). Buckingham: Published by Open University Press 2002: 295.

2. McDonagh MS, Smith DH, Goddard M. Measuring appropriate use of acute beds. A systematic review of methods and results. Health Policy. 2000;53(3):157–84.

3. Health at a Glance 2017: OECD Indicators. Paris: OECD Publishing. http://dx.doi.org/10.1787/health_glance-2017-en.

4. Saltman RB, Durán A, Dubois HFW. Governing Public Hospitals. Reform strategies and the movement towards institutional autonomy. World Health Organization: European Observatory on Health Systems and Policies 2011: 261. http://www.euro.who.int/__data/assets/pdf_file/0017/154160/e95981.pdf?ua=1.

5. Brownlee S, Chalkidou K, Doust J, et al. Evidence for overuse of medical services around the world. *Lancet*. 2017;390(10090):156-168. Published online 2017 Jan 9. doi: [10.1016/S0140-6736(16)32585-5].

6. Maslow K, Ouslander JG. Measurement of potentially preventable hospitalizations. Prepared for the long-term quality alliance. February 2012: 86.

7. Purdy S, Griffin T. Reducing hospital admissions. *BMJ*. 2008 Jan 5; 336(7634): 4–5. doi: [10.1136/bmj.39394.402465.BE]

8. Esmaili A, Ravaghi H, Seyedin H, et al. Developing of the Appropriateness Evaluation Protocol for Public Hospitals in Iran. *Iran Red Crescent Med J*. 2015 Mar; 17(3): e19030. Published online 2015 Mar 1. doi: [10.5812/ircmj.19030]

9. Liu W, Yuan S, Wei F, et al. Reliability and Validity of the Chinese Version Appropriateness Evaluation Protocol. *PLoS One*. 2015;10(8):e0136498. Published 2015 Aug 25. doi:10.1371/journal.pone.0136498.

10. Pandey A, Ploubidis GB, Clarke L, et al. Hospitalisation trends in India from serial cross-sectional nationwide surveys: 1995 to 2014. *BMJ Open*. 2017;7(12):e014188. Published 2017 Dec 19. doi:10.1136/bmjopen-2016-014188.

11. Kastor A, Mohanty SK. Disease and age pattern of hospitalisation and associated costs in India: 1995–2014. *BMJ Open* 2018;8:e016990. doi:10.1136/ bmjopen-2017-016990

12. Duckett S, Breadon P. Controlling costly care: a billion-dollar hospital opportunity. Grattan Institute Report No. 2014-2. March 2014: 51.

13. Banham D, Chen T, Karnon J, et al. Sociodemographic variations in the amount, duration and cost of potentially preventable hospitalisation for chronic conditions among

For peer review only

Aboriginal and nonAboriginal Australians: a period prevalence study of linked public hospital data. *BMJ Open* 2017;7:e017331. doi:10.1136/ bmjopen-2017-017331

14. Khosravizadeh O, Vatankhah S, Bastani P, et al. Factors affecting length of stay in teaching hospitals of a middle-income country. *Electron Physician*. 2016 Oct; 8(10): 3042–3047. Published online 2016 Oct 25. doi: 10.19082/3042

15. Nekoei MM, Amiresmaili M, Goudarzi R, et al. Investigating the Appropriateness of Admission and Hospitalization at a Teaching Hospital: A Case of a Developing Country. *Iran J Public Health*. 2017;46(12):1720-1725.

16. Lekhan VN, Rudiy VM, Shevchenko MV, et al. Ukraine: Health system review. Health Systems in Transition, 2015; 17(2): 1–153. http://www.euro.who.int/__data/assets/pdf_file/0018/280701/UkraineHiT.pdf

17. European Health for All database (HFA-DB). https://gateway.euro.who.int/en/datasets/european-health-for-all-database. Updated: 15 June 2018.

18. Slabkij GO, Podoljaka VL. Use of material and technical resources as the basis of the reform of the stationary health sector of a large industrial city. *Visnyk social'noi' gigijeny ta organizacii' ohorony zdorov'ja Ukrai'ny*. 2004;2:62-66.

19. Smijanov VA. Analysis of outpatient clinics and inpatient facilities in a modern large city (for example, Sumy). *Visnyk social'noi' gigijeny ta organizacii' ohorony zdorov'ja Ukrai'ny*. 2004;2:8-61.

20. Lekhan VM, Shherbinina MB, Volchek VV. Reserves for improving efficiency in the stationary health sector. *Medychni perspektyvy*. 2006;3:73-81.

21. Gapon VO, Lysak VP, Durdykulijeva NI. Expert assessment of the quality of provision of medical care at the secondary level in the agro-industrial region. *Ohorona zdorov'ja Ukrai'ny*. 2008;4(32):22-23.

22. Lekhan VM, Hinzburh VG, Kriachkova LV, et al. A new mode of primary health care--pilot regions experienc. *Wiad Lek*. 2014;67(2 Pt 2):210-4.

23. Pariy VD, Hryshchuk SM, Borys VM. The main approaches to the definition of a hospital intensive care when creating hospital districts *Scientific Journal «ScienceRise»*. 2015;10/3 (15): 97-100.

24. Bugro VI. Study of validity of hospitalization and bed capacity use of specialized hospital health institutions of secondary medical care. *Visnyk naukovyh doslidzhen'*. 2016;1:6-9. doi 10.11603/1681-276X.2016.1.6086.

25. Gertman PM, Restuccia JD. The appropriateness evaluation protocol: a technique for assessing unnecessary days of hospital care. *Med Care*. 1981 Aug;19(8):855-71.

For peer review only

26. Lang T, Liberati A, Tampieri A, et al. A European version of the Appropriateness Evaluation Protocol. Goals and presentation. The BIOMED I Group on Appropriateness of Hospital Use. *Int J Technol Assess Health Care*. 1999 Winter;15(1):185-97.

27. Strumwasser I, Paranjpe NV, Ronis DL, et al. Reliability and validity of utilization review criteria. Appropriateness Evaluation Protocol, Standardized Medreview Instrument, and Intensity-Severity-Discharge criteria. *Med Care*. 1990 Feb;28(2):95-111.

28. Panis LJ, Verheggen FW, Pop P. To stay or not to stay. The assessment of appropriate hospital stay: a Dutch report. *Int J Qual Health Care*. 2002 Feb;14(1):55-67.

29. Kaya S, Erdem Y, Doğrusöz S, et al. Reliability of a hospital utilization review method in Turkey. *Int J Qual Health Care*. 1998 Feb;10(1):53-8.

30. Sangha O, Schneeweiss S, Wildner M, et al. Metric properties of the appropriateness evaluation protocol and predictors of inappropriate hospital use in Germany: an approach using longitudinal patient data. *Int J Qual Health Care*. 2002 Dec;14(6):483-92.

31. Dalkey NC. The Delphi method: An experimental study of group opinion. Santa Monica: Rand.org. 1969: 79. http://www.rand.org/content/dam/rand/pubs/research_memoranda/2005/RM5888.pdf.

32. Altman DG. Practical statistics for medical research. London: Chapman and Hall 1991: 611.

33. Measures of Diagnostic Accuracy: Basic Definitions. *EJIFCC*. 2009;19(4):203-11. Published 2009 Jan 20.

34. McPherson K, Gon G, Scott M. International Variations in a Selected Number of Surgical Procedures. Health Working Papers No. 61. Paris: OECD Publishing 2013. http://dx.doi.org/10.1787/5k49h4p5g9mw-en.

35. Nolte E, McKee M. Caring for people with chronic conditions. A health system perspective. New York: Open University Press 2008: 290.

36. Freund T, Campbell SM, Geissler S, et al. Strategies for Reducing Potentially Avoidable Hospitalizations for Ambulatory Care–Sensitive Conditions. *Ann Fam Med*. 2013 Jul-Aug;11(4):363-70. doi: 10.1370/afm.1498.

37. Assessing health services delivery performance with hospitalizations for ambulatory care sensitive conditions. Working document. World Health Organization : Regional Office for Europe April 2016: 52. http://www.euro.who.int/__data/assets/pdf_file/0010/305875/Assessing-HSD-performance-with-ACSH.pdf.

For peer review only

38. Schüssler-Fiorenza Rose SM, Stineman MG, Pan Q, et al. Potentially Avoidable Hospitalizations among People at Different Activity of Daily Living Limitation Stages. *Health Serv Res*. 2017 Feb;52(1):132-155. doi: 10.1111/1475-6773.12484. Epub 2016 Mar 17.

39. Chun SY, Kim W, Park EC. Disparities in avoidable hospitalization by income in South Korea: data from the National Health Insurance cohort. *Eur J Public Health*. 2018 Sep 26. doi: 10.1093/eurpub/cky198.

40. Afonso MPD, Shimizu HE, MerchanHamann E, et al. Association between hospitalisation for ambulatory care-sensitive conditions and primary health care physician specialisation: a cross-sectional ecological study in Curitiba (Brazil). *BMJ Open* 2017;7:e015322. doi:10.1136/ bmjopen-2016-015322.

41. Gibson OR, Segal L, McDermott RA. A systematic review of evidence on the association between hospitalisation for chronic disease related ambulatory care sensitive conditions and primary health care resourcing. *BMC Health Serv Res*. 2013 Aug 26;13:336. doi: 10.1186/1472-6963-13-336.

42. Corbella X, Ortega B, Juan A, et al. Alternatives to conventional hospitalization for improving lack of access to inpatient beds: A 12-year cross-sectional analysis. *J Hosp Administ*. 2013;2:9-21. doi: https://doi.org/10.5430/jha.v2n2p9.

43. Behrndt EM, Straubmeier M, Seidl H, et al. The German day-care study: multicomponent non-drug therapy for people with cognitive impairment in day-care centres supplemented with caregiver counselling (DeTaMAKS) – study protocol of a cluster-randomised controlled trial. *BMC Health Serv*. Res. 2017; 17: 492. doi: [10.1186/s12913-017-2422-x].

44. Fedotkina SA, Karajlanov MG, Rusev IT. Rational use of hospital-replacing technologies and forms of medical care. *Vestnik Sankt Peterburgskogo gosudarstvennogo universiteta. Medicina.* 2017; 12 (2): 179-189; doi: 10.21638/11701/spbu11.2017.207.

45. Miani C, Ball S, Pitchforth E, et al. Organisational interventions to reduce length of stay in hospital: a rapid evidence assessment. *Health Services and Delivery Research* 2014;2:1–178.

46. Huckfeldt PJ, Kane RL, Yang Z, et al. Degree of Implementation of the Interventions to Reduce Acute Care Transfers (INTERACT) Quality Improvement Program Associated with Number of Hospitalizations. *J Am Geriatr Soc*. 2018 Sep;66(9):1830-1837. doi: 10.1111/jgs.15476. Epub 2018 Aug 10.

For peer review only

47. Fernandez JL, McGuire A, Raikou M. Hospital coordination and integration with social care in England: The effect on post-operative length of stay. *Journal of Health Economics.* 2018 Sep; 61: 233-243. doi.org/10.1016/j.jhealeco.2018.02.005

48. Holder H, Kumpunen S, Castle-Clarke S, Lombardo S. Managing the hospital and social care interface. Interventions targeting older adults. Research report March 2018: 76. https://www.nuffieldtrust.org.uk/files/2018-03/hospital-and-social-care-interface-final-web.pdf

For peer review only

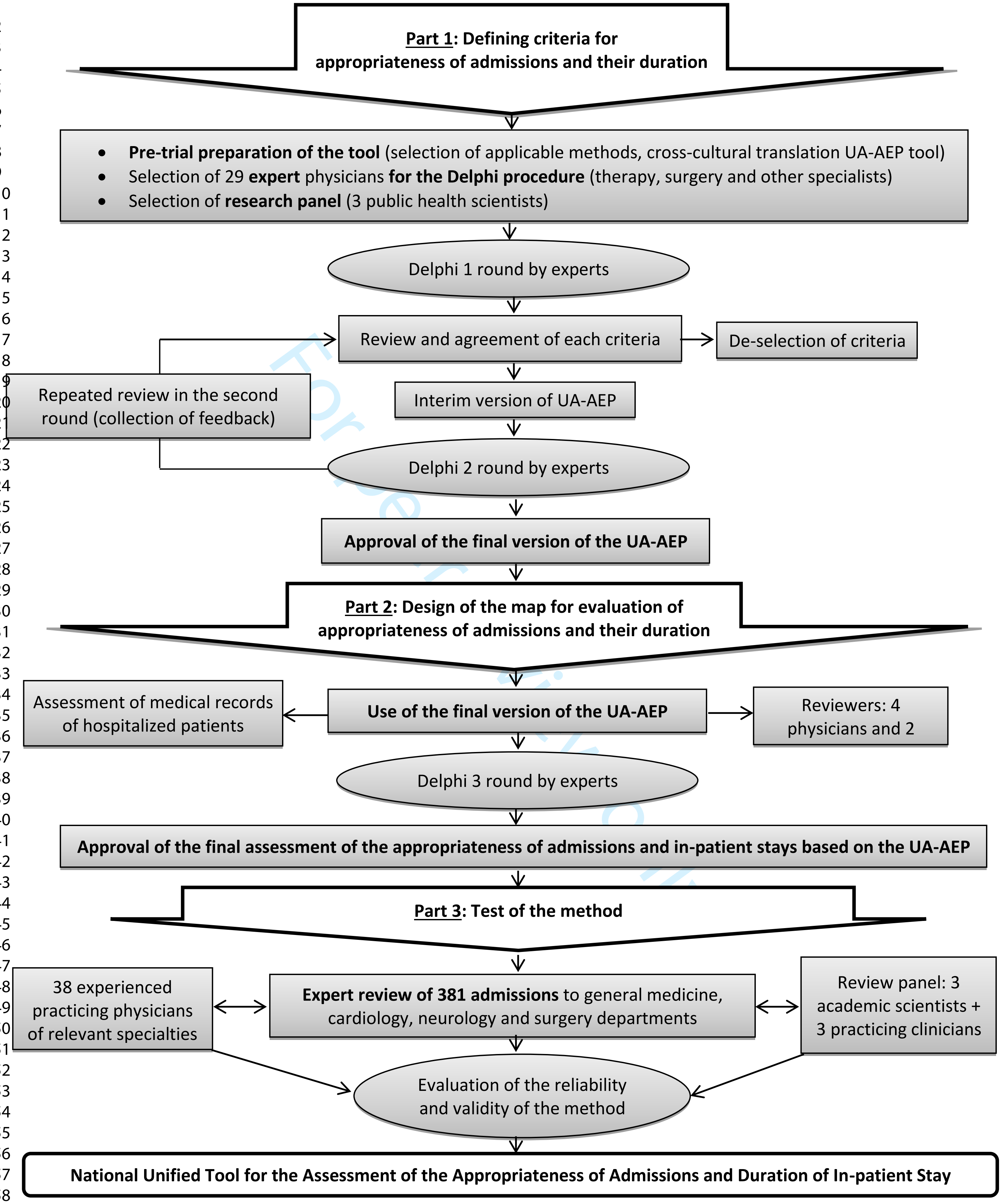

Figure 1 Schematic presentation of the steps followed during the development and evaluation of the method

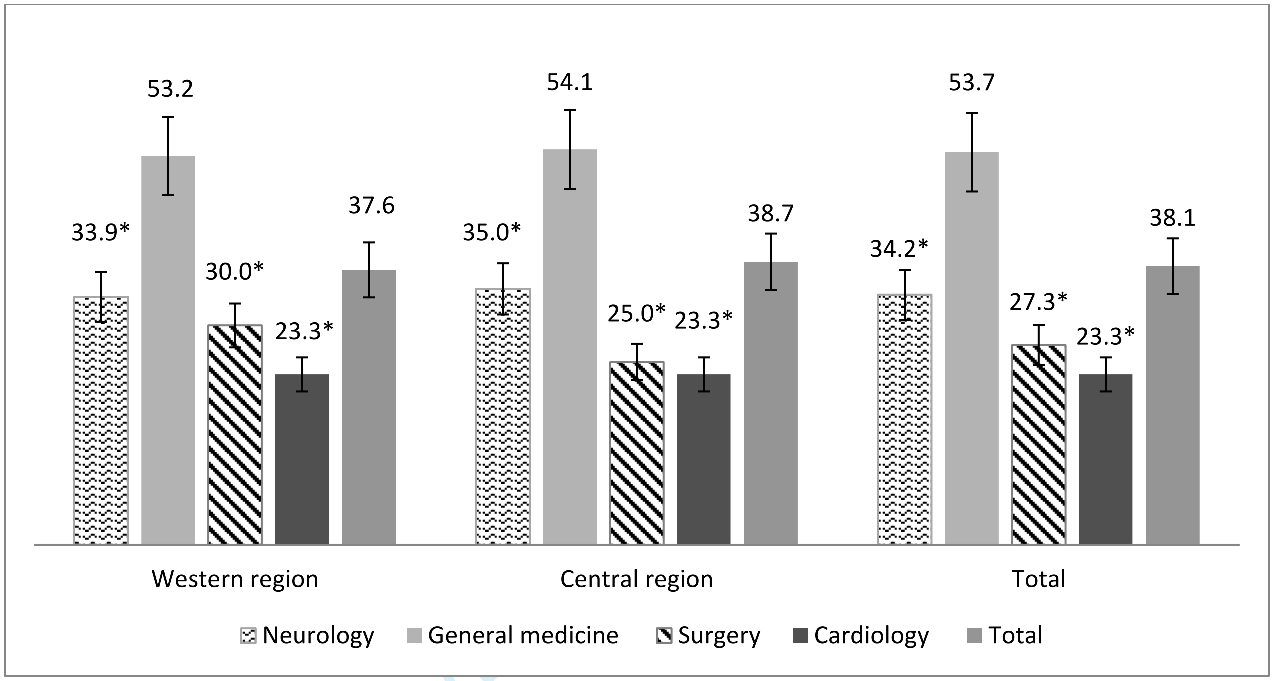

Figure 2 Shares of inappropriate admissions by department types, total and in the two regions of Ukraine (%, 95% CI)

Note: * - p<0.001 compared with the general medicine department

Supplementary file 1

CRITERIA FOR APPROPRIATENESS OF HOSPITAL ADMISSIONS AND LENGTH OF INPATIENT STAY

Admission criteria

A. Severity of illness

1. Sudden loss of consciousness or disorientation (coma or unresponsiveness)
2. Acute confusional state, but not because of alcohol or narcotic intoxication
3. Acute loss of sight or hearing
4. Acute loss of ability to move part of the body
5. Acute ataxia (except for repeated paroxysms of acute ataxia in case of period/ episodic ataxias)
6. Persistent fever >38.0 C for 5 days without established diagnosis
7. Active bleeding which can lead to circulatory violations in case of absent homeostasis
8. Progressing acute neurological disorders
9. Acute or progressive sensory, motor, circulatory or respiratory disorder that can incapacitate the patient (inability to move, feed, breathe, urinate, etc.)
10. Unbearable pain (that cannot be managed by outpatient services (including primary or specialized outpatient or emergency service)
11. Acute abdominal pain with laboratory, ultrasound, X-ray disturbances lasting more than 6 hours
12. Severe pain, sudden edema, paling with cold peripheral limbs and decreased distal pulse
13. Inability to empty bowels or intestine movements (during the previous 24 hours), which cannot be attributed to the main neurological disorder
14. Patient's potentially life-threatening condition - for supervision (head injury, etc.)
15. Severe electrolyte/blood gas /CBC abnormality (any of the following, in mmol/L):
    - 123 < Na > 156
    - 2,5 < K > 6,0
    - 20 < HCO_3 > 36
    - 7.30 < Arterial blood pH > 7.45
    - 20 < CO_2 > 36 (unless chronically abnormal)
    - BUN (blood urea nitrogen) > 15 mg/dL (with increasing dynamics)
    - 3.9 ≤ FBS (Fasting blood sugar) ≥ 22.4 (associated with symptoms of weight reduction for no apparent reasons, polyuria, and polydipsia)
16. ECG evidence of acute ischemia (suspicion of a new myocardial infarction).
17. Acute heart rate disturbances (including ventricular fibrillation) and / or circulation with signs of hemodynamic disorders
18. Signs or symptoms of acute hematological disorders.

B. Intensity of Medical Services

1. Surgical or another procedure requiring equipment or other technologies available only in an inpatient facility (pre-surgery preparation, long – for more than 24 hours – clinical monitoring or supervision)
2. Invasive diagnostic procedures on the central nervous system.
3. Treatment requiring frequent dose adjustments in a potentially severe condition under direct medical supervision

C. Medical Care / Life Support Services

1. Intermittent or continuous use of an artificial lung ventilation at least 3 times per 24 hours.
2. Parenteral therapy - intermittent (2 or more times per day) or continuous intravenous fluid administration with any component / intravenous infusion of medication and / or fluid substitution (does not include tube nutrition).
3. Continuous monitoring of vital signs – at least every 30 mins, for at least 4 hours

For peer review only

D. Other admission criteria
1. Indications for hospitalization defined in the national unified clinical protocols (for specific diseases)<br>2. Advanced hospitalization with sufficiently documented justification in case of a threat of potential deterioration of the patient's condition (e.g., pregnant women with a high perinatal risk during the perinatal period etc.)<br>3. The need for isolation in case of an epidemiological or social threat (e.g., from the side of patients with psychological disorders etc.)<br>4. Other<br><br>**Note.** Hospitalization is not justified for the following cases:<br>• Provision of care is possible on an outpatient basis or using alternative to inpatient services (day care) irrespective of the remoteness of patient's place of living to the care facility (organization of boarding care for these patients if necessary);<br>• The patient mostly requires social care (lonely people etc.);<br>• The decision on hospitalization is made solely based on the referral from the primary care doctor or a specialist doctor;<br>• The need for hospitalization is caused by rigid rules of the Soviet period (for issuance of a disability status, for the conclusion on ability for military service etc.) and is not associated with the real condition of the patient and intensity of care required.
**Duration of inpatient stay**
Durations of inpatient stay are defined based on case-specific assessment of the dynamics of a patient condition.<br><br>**Note.** Inpatient stay is considered unjustified in the following cases:<br>• If a diagnostic test of the patient may be performed without or before patient's hospitalization;<br>• When based on his/her condition, the patient can continue treatment on a day-care or outpatient basis (irrespective of the remoteness of the patient's place of living from the inpatient facility – boarding care can be organized for such patients if necessary);<br>• When stay in an inpatient facility is caused by the patient's social problems (lonely people, those who have unsatisfactory living conditions, etc.).

For peer review only

Supplementary file 2

Assessment tool for the appropriateness of hospitalization and hospital stay

CARD FOR EXPERT ASSESSMENT OF THE APPROPRIATENESS OF HOSPITALIZATION AND DURATION OF INPATIENT STAY

The expert assessment uses data from the medical record of the hospital episode (form 003/o), health care facility passport or audit of the secondary (specialized) care (Order of the MoH of Ukraine no. 637 dd. 17.08.2012 "On Approval of the Guidelines for the Procedure for Audit of Secondary (Specialized) Health Care", and other available data. The expert applies criteria of appropriateness of admission and patient stay provided separately.

City/town/village ______________________________
Hospital ______________________________
Department______________________________
Inpatient's medical record no.______________ **Date of the expert review** _______ ______ ______
Full name of the expert______________________________

I. GENERAL DATA AND CONDITION AT THE TIME OF INPATIENT ADMISSION

1. Age: _________ **full years** **2. Sex:** 1 – male; 2 – female
3. Residence: 1 – rural 2 – urban
4. Living: 1. With a family (specify the number of people, if possible) ______
2. Alone
3. Difficult to tell
5. Clinical diagnosis at the time of admission:
____
6. Diagnosis at discharge (specify the name and ICD code)
Principal ______________________________ICD_______
Complication of the principal disease ______________________________ICD_______
____
Complication of treatment (incl. post-surgical) ______________________________ICD_______
____
Secondary disease(s) that influenced the course of the principal disease ______________________________
______________________________ICD____
7. Inpatient admission:
1. Planned
2. Urgent - ________hours after the start of the disease or injury
8. During the current year, in relation to the disease, the patient:
1. Is admitted for the first time 2. Has been admitted ________ times
9. The patient is referred by:
1 – primary care specialist
2 – outpatient facility specialist *(indicate specialization)* ______________________________
3 – ambulance unit (emergency medicine)
4 – self-referred
5 – other inpatient facility______________________________ *unit specialization* ______________________________
6 - other *(specify)*______________________________
10. Overall condition of the patient state at the time of admission:
1 – satisfactory
2 – moderate severity
3 – severe
4 – critical

II. APPROPRIATENSS OF ADMISSION

11. Please indicate if the admission is appropriate according to the criteria of the appropriateness of admission:
1. Yes, it is appropriate to this inpatient facility
2. Yes, it is appropriate, but to a higher-level inpatient facility (such as another secondary care facility for intensive care, or a tertiary care facility)
3. Yes, it is appropriate but to another secondary inpatient facility for elective care
4. Inappropriate
5. Difficult to tell

Please provide any comments you have here ______________________________
____

For peer review only

12. What justifies the appropriateness of admission (multiple choice allowed)

1. Intensity of the required and provided services
2. Severity of the case
3. Compliance with existing national clinical protocols
4. Other (specify) ____

13. <u>If the admission is not appropriate, or if it is appropriate for another inpatient facility of a different level (see health care facility passport or audit materials), please specify the reasons</u> (multiple choices allowed):

1. Overestimation of the severity of the case (incl. the threat of unexpected progress of the disease)
2. Inpatient admission is likely organized to occupy the beds of the facility
3. Premature admission (e.g., admission on Monday for a surgery planned for Wednesday)
4. There was no time or conditions for a quick examination, or a consultation was required to decide on admission
5. The admission was to reduce the cost of examination or patient's costs, e.g. admission of a patient living far from the hospital to avoid accommodation/hotel costs
6. Ignorance of medical standards of indications for inpatient admission (outdated practice)
7. Admission to a lower level inpatient care was impossible due to funding fragmentation
8. Admission was made at the request of a general practitioner (family doctor) or a specialist doctor.
9. Admission was made on the demand of a patient or his/her family
10. Admission was legally mandatory:
    - 10.1. To receive a medical conclusion on disability
    - 10.2. To receive a medical conclusion on eligibility to military service
    - 10.3. Other (specify) ____
11. Admission is related to social problems of the patient
    - 11.1. No opportunity to receive care at home (the patient is lonely, or other family members are disabled)
    - 11.2. Unfavorable conditions at home
    - 11.3. Other (specify) ____

14. What measures, in your opinion, could improve the appropriateness of admission in this case (multiple choices allowed):

1. Availability of clear indications/criteria for admission to inpatient facilities of different levels
2. Creation of conditions for diagnostic examination in the outpatient setting or day care facility
3. Removal of financial limitations concerning inpatient admission to a health care facility of the required level
4. Bringing facility's capacity (number of beds) in line with the need of certain type and intensity of care
5. Strict observance of existing unified clinical protocols
6. Creation of an opportunity to perform diagnostic examination in the facility to timely decide whether admission is required
7. Organization of a boarding house at the facility for people from remote locations
8. Elimination of the normative for planning number of personnel linked to the number of beds
9. Clear delimitation of indications to medical and social care
10. Creation of conditions for provision of social care where indicated, incl. round the clock care (development of social care facilities network)
11. Other (specify)____

III. JUSTIFICATION OF THE DURATION OF INPATIENT STAY

15. The patient required:

1. Intensive conservative treatment at this inpatient facility
2. Intensive conservative treatment at inpatient facility of a higher level (e.g., s/he was receiving care at a secondary care facility while requiring tertiary care)
3. Intensive conservative treatment at inpatient facility of a lower level (e.g., s/he was receiving care at a tertiary care facility while requiring secondary care)
4. Intensive surgical treatment at this inpatient facility
5. Intensive surgical treatment at inpatient facility of a higher level (e.g., s/he was receiving care at a secondary care facility while requiring tertiary care)
6. Intensive surgical treatment at inpatient facility of a lower level (e.g., s/he was receiving care at a tertiary care facility while requiring secondary care)
7. Elective care at this inpatient facility:
   7.1. With the need for the 24/7 medical assistance at the facility;
   7.2. Without the need for the 24/7 assistance at the facility

For peer review only

8. Elective care at a lower level inpatient facility
9. Social care:
   9.1. At home
   9.2. With temporary accommodation
   9.3. With permanent accommodation
10. No inpatient care
11. Other (specify) ____

IIIA. Diagnostic Stage of Inpatient Stay

16. Duration of the diagnostic stage (the period from inpatient admission to the start of appropriate treatment).
The decision about the final diagnosis is made on of the _________ day or within ________ hours of stay at the facility

17. Duration of the diagnostic stage:
1. Reasonable 2. Unnecessary long 3. Difficult to evaluate

18. The diagnosis recorded at the inpatient facility was:
1. Correct
2. Incorrect (incorrect disease entity/not all the diseases determined)
3. Inaccurate (incorrect form/stage/extent/severity/clinical manifestations of the disease)
4. Difficult to evaluate

IIIB. Treatment Stage of Inpatient Stay

19. Proper treatment (after confirmation of the final diagnosis) started:
on the ________ day or within ________ hours of stay at the inpatient facility

20. Duration of the treatment period (from the final diagnosis to discharge)
__________ days

21. Surgical interventions:
1. None performed 2. Performed (specify)
____Surgical code___________

22. Resuscitation/intensive care
1. No 2. Yes

23. Duration of the treatment stage:
1.Reasonable 2. Unnecessary long 3. Difficult to evaluate

24. <u>If the duration of the inpatient stay was not justified, specify the reasons to support this opinion (multiple choices are allowed</u>):

<u>24.1. General</u>
1. Premature admission
2. The need to occupy excessive beds capacity of the inpatient facility
3. Inadequate or absent clinical protocols
4. Inadequate or absent local protocols, or not adhered
5. Ignorance of medical standards (outdated practice)
6. Admission to a facility where care could not be provided adequately to patient's condition (e.g., the patient required intensive care, but the facility did not have the equipment for it)
7. Lack of equipment and materials at the facility: no possibility to use modern treatment and diagnostic technology (such as MRI machine for diagnostics, laparoscopic equipment for certain surgical interventions, necessary supplies) – please specify____
8. Lack of medical personnel or skills (specify) ____
9. Inefficient organization of specialized medical consultation
10. Lack of opportunity to use remote telemedical assistance
11. Delays in consent (inability of making fast decisions, hesitation) for the patient and his/her family concerning diagnostic/treatment/surgical procedures
12. Patient's refusal to receive the suggested treatment
13. Other (specify) ____

<u>24.2. At the diagnostic stage</u>
1. Unreasonable duplication of diagnostic laboratory and instrumental exams conducted at previous stages of care or in other inpatient care setting
2. Potential financial incentives to increase the scope and number of the examination
3. Inefficient schedule of operation of the diagnostic units (laboratory etc.) (such as impossibility of conducting diagnostic tests round the clock)
4. Inefficiencies in the interaction with other facilities required to confirm a diagnosis
5. Other (specify)____

For peer review only

24.3. At the treatment stage

1. Absent/insufficient capacities in terms of life support services (anesthesiology and intensive care department)
2. Lack of practice/organizational issues not allowing to discharge patients after deciding on inappropriateness of inpatient admission based on a preliminary observation
3. Necessity of moving/transporting the patient to another health care facility (reduction of the duration of inpatient stay)
4. Deficit (absence) of alternative care - no capacity or practice of use of day care facilities for post-acute care
5. Complications of the case due to incorrect diagnosis
6. Complications of the case due to inadequate treatment
7. Doctor's overcautiousness regarding the discharge time, incl. due to stereotypes (e.g., discharge after receiving a pathologist's report etc.), please specify ______________________________
8. Inefficient planning of the discharge (e.g., no discharge on week-ends)
9. Premature discharge in order to improve the performance indicators of the facility because of a high probability of death of the patient
10. The patient residing in remote location stayed longer at the facility because s/he was unable to get home on the day of discharge
11. Social problems of the patient:
    11.1. No opportunity to receive care at home (the patient is lonely, or other family members are disabled)
    11.2. Unfavorable conditions at home
12. Other (specify) ______________________________
____

25. Estimates of the duration of inpatient stay

Treatment stage	***Actual bed-days***	***Recommended bed-days***
**I. Diagnostic**		
**II. Treatment**		
**Total**		

Please provide any comments you have ______________________________
____

26. Which measures, in your opinion, could improve the appropriateness of inpatient admission and stay (multiple choices allowed):

1. Upgrading anesthesiology and intensive care departments to align with the needs in terms of capacity, equipment and staffing
2. Rightsizing capacities of the facility (e.g. number of beds) according to the needs of the specific type and intensity of care
3. Creation of conditions for diagnostics and follow-up care in a day care or outpatient setting
4. Organization of a boarding house at the facility for people from remote locations requiring post-acute care, provision of care at a day care or in an outpatient setting
5. Addressing financial limitations preventing admission to an inpatient facility of the adequate level
6. Setting up wards for short-term stay for patients in emergency health care department/admission and examination department
7. Better adherence to clinical protocols
8. Clear criteria for discharge from inpatient facilities of different levels
9. Provision of needed materials and equipment
10. Elimination of the normative linking the staff size to the number of beds
11. Clear delimitation of indications to medical (including rehabilitation, hospice) and social care
12. Creation of conditions for provision of social care where indicated, incl. round the clock care (development of social care facilities network)
13. Other (specify) ______________________________
____

Other comments: ____________________________________
____
____

Signature of the Expert ______________________

For peer review only

Supplementary table 1

Inappropriate hospitalizations and unnecessary hospital days (results of the pilot)

Department	Facility	Patients	Bed-days	Inappropriate hospitalizations		Admission to a wrong department/facility		Unnecessary inpatient stay		Inappropriate inpatient stay at wrong departments/facilities	
		Number	Number	Number/%	95% CI	Number/%	95% CI	Number/% of bed-days	95% CI	Number/% of bed-days	95% CI
**General medicine**	Facility 1	40	426	29/72.5%	58.7% to 86.3%	2/5.0%	-1.8% to 11.8%	327/76.8%	72.7% to 80.8%	12/2.8%	1.2% to 4.4%
	Facility 2	30	297	12/40.0%	22.5% to 57.5%	3/10.0%	-0.7% to 20.7%	120/40.4%	34.8% to 46%	21/7.1%	4.2% to 10%
	Facility 3	30	311	13/43.3%	25.6% to 61.1%	1/3.3%	-3.1% to 9.8%	170/54.7%	49.1% to 60.2%	12/3.9%	1.7% to 6%
	**Total**	**100**	**1034**	**54/54.0%**	**44.2% to 63.8%**	**6/6.0%**	**1.3% to 10.7%**	**617/59.7%**	**56.7% to 62.7%**	**45/4.4%**	**3.1% to 5.6%**
**Cardiology**	Facility 1	41	537	10/24.4%	11.2% to 37.5%	-	-	231/43.0%	38.8% to 47.2%	-	-
	Facility 3	29	249	6/20.7%	5.9% to 35.4%	-	-	70/28.1%	22.5% to 33.7%	-	-
	**Total**	**70**	**786**	**16/22.9%**	**13.0% to 32.7%**	-	-	**301/38.3%**	**34.9% to 41.7%**	-	-
**Neurology**	Facility 1	30	306	2/6.7%	-2.3% to 15.6%	-	-	97/31.7%	26.5% to 36.9%	-	-
	Facility 2	59	645	14/23.7%	12.9% to 34.6%	-	-	253/39.2%	35.5% to 43%	-	-
	Facility 3	30	301	11/36.7%	19.4% to 53.9%	3/10.0%	-0.7% to 20.7%	149/49.5%	43.9% to 55.1%	25/8.3%	5.2% to 11.4%
	**Total**	**119**	**1252**	**27/22.7%**	**15.2% to 30.2%**	**3/2.5%**	**-0.3% to 5.3%**	**499/39.9%**	**37.1% to 42.6%**	**25/2.0%**	**1.2% to 2.8%**
**Surgery**	Facility 1	41	465	5/12.2%	2.2% to 22.2%	-	-	244/ 52.5%	47.9% to 57%	-	-
	Facility 2	30	181	10/33.3%	16.5% to 50.2%	2/6.7%	-2.3% to 15.6%	40/22.1%	16.1% to 28.1%	10/5.5%	2.2% to 8.9%
	Facility 3	21	144	5/23.8%	5.6% to 42.0%	5/23.8%	5.6% to 42.0%	45/31.3%	23.7% to 38.8%	27/18.8%	12.4% to 25.1%
	**Total**	**92**	**790**	**20/21.7%**	**13.3% to 30.2%**	**7/7.6%**	**2.2% to 13.0%**	**329/41.6%**	**38.2% to 45.1%**	**37/4.7%**	**3.2% to 6.2%**
**Total**		**381**	**3862**	**117/30.7%**	**26.1% to 35.3%**	**16/4.2**	**2.2% to 6.2%**	**1746/45.2%**	**43.6% to 46.8%**	**107/2.8%**	**2.3% to 3.3%**

For peer review only

Supplementary table 2

Medical records included in the study

Region / Level / Profile	Western region			Central region			Total for 2 regions		
	Secondary care	Tertiary care	Both levels	Secondary care	Tertiary care	Both levels	Secondary care	Tertiary care	Both levels
Neurology	161	10	171	40	20	60	201	30	231
General medicine	71	40	111	120	50	170	191	90	281
Surgery	40	50	90	80	28	108	120	78	198
Cardiology	20	10	30	20	40	60	40	50	90
Total	292	110	402	260	138	398	552	248	800

For peer review only

Supplementary table 3

The possible reasons for inappropriate hospital admissions and inpatient stays (expert opinions)

Reason	Frequency, % (95% CI)
An attempt to keep the current capacities in the inpatient facility	37.6 (30.5 to 44.6)
Hospital admission is defined by the need to fill inpatient beds and improve their occupancy	22.7 (16.6 to 28.7)
Absence of alternatives to inpatient types of care or capacities to treat patients after their discharge	21.0 (15.1 to 26.9)
Absence of organizational possibilities to discharge patients after a decision is made that it is unnecessary to extend inpatient stay	9.9 (5.6 to 14.3)
Lack of timely examinations or consultations for necessary treatment	12.7 (7.8 to 17.6)
Inefficient cooperation with other professionals and facilities that need to be involved to establish a diagnosis	6.6 (3.0 to 10.3)
Non-adherence to clinical protocols or their outdated provisions	7.2 (3.4 to 10.9)
Overestimated severity of patient's condition	20.4 (14.6 to 26.3)

For peer review only

Supplementary table 4

Key proposed actions to prevent inappropriate hospital admissions or unnecessary inpatient days

Actions	Frequency of suggesting by experts, % (95% CI)
To use clear indications or criteria for admission to inpatient facilities of different levels	32.6 (25.8 to 39.4)
To create conditions for diagnostic examinations and post-discharge care [follow-up] in outpatient facilities or on a day-care basis	44.8 (37.5 to 52.0)
To stop using personnel employment normative based on a facility or department's beds capacity	24.3 (18.1 to 30.6)
To bring the network and capacities of inpatient care facilities in compliance with the actual needs in a specific type and intensity of care	14.9 (9.7 to 20.1)
To arrange accommodation/ boarding facilities for persons living far from a treatment facility	33.7 (26.8 to 40.6)
To use clear criteria for patients' discharge from hospitals of different levels	19.9 (14.1 to 25.7)

For peer review only

Title and Abstract

1. Title	Assessment of appropriateness of hospitalizations in Ukraine: analytical framework, method, and findings (page 1)
2. Abstract	Objectives: This article reviews the applicability of a customized version of the Appropriateness Evaluation Protocol (AEP) to evaluate the magnitude of inappropriate hospitalizations in two regions of Ukraine.<br><br>Data and methods: The original AEP was modified to develop a customized tool, which included criteria for the appropriateness of hospitalization and duration of inpatient stay. The customization of the tool followed the Delphi procedure. We randomly selected 381 medical records to test the feasibility and reliability of the method and 800 medical records to evaluate the scope of inappropriate hospitalizations. We used descriptive and analytical statistics, ROC analysis, and Cohen's kappa to check the consistency between the findings of primary reviewers and experts.<br><br>Result: We observed high levels of agreement in conclusions of primary reviewers (reference standard) and experts during testing of the reliability and validity of the method. The external validity check showed that the use of the tool by different experts provided high accuracy: 95.1 sensitivity, 76.6 specificity, and AUC=0.948 (p<0.001) for analysis of the appropriateness of admissions; 95.3 sensitivity, 84.7 specificity, and AUC=0.900 (p=0.001) for the duration of hospitalizations. Cohen's kappa coefficient (κ) indicated agreement in expert evaluations of 0.915 (95% CI 0.799 to 1.000) and 0.812 (95% CI 0.749 to 0.875), respectively.<br><br>We found that over one-third of admissions (38.1%; 95% CI 33.9 to 43.5) and over half of total bed-days were unnecessary (57.4%; 95% CI 56.4 to 58.5). The highest levels of stay were observed in hospitals' general medicine departments (64.6%; 95% CI 63.0 to 66.3) compared to other departments included in the analysis.<br><br>Conclusion: The proposed method is robust in assessing the appropriateness of hospitalizations and duration of inpatient stays. The quantified levels of unnecessary hospital care indicate the need for improving efficiency and quality of care and optimizing the excessive hospital capacities in Ukraine. (page 2)

Introduction	*Why did you start?*
3. Problem Description	Hospital care is a core part of any national health system; it influences health outcomes of the population and requires significant funding. [1, 2] Still, the use of hospital care has distinct features across the globe and ranges in terms of the level of effectiveness. [3] If inpatient care fails to improve the health outcome when compared with less invasive or less intensive non-hospital care, it is considered an overuse of inpatient services. [4, 5, 6]<br><br>Overuse of hospital care is typical for many countries around the world, ranging from 1% to 54% in general [5], and up to 90% for some diseases. [7] Unnecessary hospitalizations are a matter of concern for many nations, specifically due to the rising trend of healthcare expenditures and a significant proportion of hospital expenditures in overall health spending. [8 - 12]<br><br>Hospital sector expenditures are mostly dependent on admission processes, length of patient hospital stays, and advancement of medical technologies in hospital facilities. Inpatient length of stay (LOS) is the most important contributing factor influencing the use of resources and cost of care during hospitalization; it closely relates to the effectiveness of hospital treatment, the effectiveness of hospital management, and general efficiency of a health system. [13, 14, 15] (page 4)
4. Available Knowledge	Rationalization of hospital expenditures together with increased, or at least sustained, quality of hospital care are key objectives of health care reform in Ukraine. [15, 16] The hospital sector in Ukraine is oversized and inefficient by both the numbers of beds and hospitals. The number of beds per 10,000 people was 74.3 in 2016, or three times higher than in the EU; the number of hospitals per 10,000 people exceeded EU numbers by a quarter; the average length of stay was 11.2 days, or 3 days longer than for patients in the EU. [17]<br><br>Numerous studies published in Ukraine within the last 15 years have been dedicated to the use of hospital care, [18-24] and the research has shown significant inefficiencies in the hospital sector. Reported results, however, vary substantially: between 8.2 and 70.6% inappropriate hospitalizations, and 26.4 - 86.5% of unnecessary bed-days. (page 4)

5. Rationale	Studies in other countries have reported similar problems with the variability of results, which led to an international recommendation from researchers that a unified method for the assessment of reasons for hospitalizations and their duration should be used, [7, 8] specifically the Appropriateness Evaluation Protocol (AEP). [25, 26] The AEP has high validity and reliability compared with other methods for assessment of inappropriate hospitalizations [2, 27] and was customized for many countries. [7, 8, 28]<br><br>The AEP method has not been used in Ukraine, where assessments are traditionally based on expert evaluations providing retrospective analyses of medical records. Such analyses are typically designed by individual experts through methods that are not validated. [18-24] Although the provision of hospital care is guided by legislation and clinical protocols, concrete and evidence-based criteria for hospitalizations are absent. Additionally, a more accurate estimate of the needs in hospital care is necessary to support the ongoing health reform in Ukraine, which is implemented improve access and quality to health care, and to address inefficiencies in service delivery. (page 4-5)
6. Specific Aims	The objective of this study was to develop and test the customized Ukraine version of the unified method for assessment of the appropriateness of hospitalizations, including reasons for admissions to hospitals and duration of hospital stays, and to evaluate the scope of inappropriate hospitalization of adults in Ukraine using a sample of hospitals from two regions. (page 5)
**Methods**	***What did you do?***
7. Context	Based on available international tools [8, 9, 25-30] and existing approaches used in Ukraine, [18-24] the tool for this study was developed and customized for the Ukrainian context in order to assess the appropriateness of hospitalizations and duration of inpatient stays. (page 5)
8. Intervention(s)	The tool included criteria for appropriateness of hospital admissions and a guide for expert evaluations of justified inpatient lengths of stay. The tool was finalized using the Delphi method for expert evaluations [31] in three rounds as schematically presented in figure 1. (page 5)<br><br>We used criteria from these versions of the UA-AEP to develop a guide that helps to extract medical information about hospital care episodes from medical records (MoH

	form 003/o) for evaluation of the appropriateness of admission decisions and justification of lengths of stay (see online supplementary file 2). (page 6)
9. Study of the Intervention(s)	Criteria for hospital admissions were included in the customized tool based on an analysis of seven versions of the AEP: original US version (US-AEP), [25] European (E-AEP), [26] Turkish (TR-AEP), [29] Dutch (D-AEP), [28] German (DE-AEP), [30] Chinese (C-AEP) [9], and Iranian version of the AEP (IR-AEP). [8] The customized Ukrainian AEP tool defined Ukraine-specific indications for hospital admission and criteria for the justification of inpatient length of stay – summarized in the Ukrainian Appropriateness Evaluation Protocol (UA-AEP) (see online supplementary file 1). (page 6)
10. Measures	To avoid the potential conflict of interests, experts represented different health care facilities, provided informed consent, received only summarized information of inputs provided before each round, and did not communicate about the study before the final approval of the modifications to the AEP. The group of reviewers controlled the adherence to the Delphi procedure. (page 6)<br><br>Piloting of the method<br><br>The proposed tool was piloted in three types of hospitals of different size in one of the largest regions in eastern Ukraine. Four department profiles were selected for the analysis: general medicine, cardiology, neurology, and surgery. These departments are representing a most typical structure of a hospital: on average, 44% of all adult patients are admitted to these departments in Ukraine.<br><br>Standard medical records were used for analysis (MoH approved Form 003/o). The two-stage approach was used to sample records for the year preceding the analysis: first, one month was randomly selected (March 2016); second, from all archived records in the four departments in March 2016, 381 cases were randomly selected. All sampled records were proportionally distributed for evaluation to 38 experts with relevant clinical backgrounds for the profile of sampled cases. Experts were selected based on their area of expertise and the results of the initial test of competencies. The total number of experts was determined based on an average anticipated workload of 10 medical records per one expert.<br><br>The results of the piloting confirmed the feasibility of the tool for data collection and analysis. The pilot data showed that about one-third of all admissions included in the

	analysis were classified as inappropriate. The length of stay in facilities was often excessive: 45.2% of all bed-days were inappropriate (see online supplementary table 1). The yielded results corresponded with the finding from a study of inappropriate hospitalizations in the same region, [20] and this confirmed the feasibility of the proposed method.<br><br>Reliability and validity of the method<br><br>The results of the pilot were verified by a review panel, consisting of three primary reviewers (public health scientists). Each primary reviewer was assisted by a clinician in the relevant clinical field (therapist, surgeon, and neurologist).<br><br>We assessed the reliability and validity of the method by comparing the evaluation results provided by 38 experts with the conclusions of the review panel. The latter was used as a reference standard for the conclusions.<br><br>Statistical methods<br><br>The dataset and statistics were prepared using Microsoft Excel (Microsoft Office 2016 Professional Plus, Open License 67528927) and STATISTICA 6.1 software (StatSoftInc., serial No. AGAR909E415822FA). We used descriptive and analytical statistics for data analysis. [32] For relative values, 95.0% confidence intervals (95% CI) were calculated based on the corrected Wald method. The assessment of the validity in the differences in relative indices was carried out according to Pearson’s Chi-square test (Chi-square test – $\chi 2$).<br><br>For the sample calculations, we used Cochran formula with p=0.95 (α=0.05) and t=1.96. The general number of all hospital admissions N for catchment area served by the hospitals included in pilot sites was 755,350 and the hospital admission rate 23.3%, making the calculation for the minimal powered sample of 275 admissions for the pilot study (sampled 381). For the main stage of the analysis, the recommended sample of 322 records (α=0,05) or 895 records (α=0,03) was calculated using “Power analysis” in STATISTICA 6.1 software for the general number of admissions in Ukraine of 8,594,210, and the level of inappropriate hospitalizations of 29.9% calculated for the pilot (sampled 800). (page 6-7)
**11. Analysis**	To analyze agreement in experts’ reviews, we used the concordance coefficient (W) and Cronbach's alpha (α). Approximation of coefficient values to 1 indicates an increase in agreement rates.

	The inter-rater agreement in experts' reviews was analyzed using overall agreement coefficient and Cohen's kappa coefficient (κ), applying the following ranges: values of 0.61-0.80 were defined as good strength of agreement, values of 0.81 and higher as very good strength of agreement. [32]<br><br>ROC curve analysis was conducted to calculate operational characteristics and the area under ROC-curve (AUC) to evaluate the discriminative capacity of the method. The relationship between the area under the ROC curve and diagnostic accuracy was assessed using the following ranges: 0.7-0.8 – good; 0.8-0.9 – very good; 0.9 – 1.0 – excellent. [33]<br><br>The statistical significance was taken at the level of p <0.05 (5%) for all tests. (page 7-8)
**12. Ethical Considerations**	Ethics statement<br><br>Confidentiality of information from medical records was kept by protecting patient identifiers. Researchers received copies of paper-based records with patient identification fields crossed out or covered. Names of physicians and hospital names were replaced with ID codes for reviewers of medical records.<br><br>All experts working with primary data signed non-disclosure agreements of confidential data and obligations to follow ethics principles of medical research statements, which are endorsed by international agreements and the Ministry of Health of Ukraine. Compliance with bioethics principles and medical deontology was confirmed in the conclusion of the Committee on bioethics and medical deontology in the State Institution "Dnipropetrovsk Medical Academy of the MOH Ukraine" (Protocol No.6 of September 16, 2016).<br><br>Patient and Public Involvement<br><br>We did not involve patients or the public in our work. (page 5)
**Results**	***What did you find?***
**13. Results**	Results of Reliability and Validity tests<br><br>The Ukraine version of the AEP proved its reliability and validity for the assessment of the hospitalizations because sensitivity and specificity measures are high for the

sample and within the selected medical specialties for evaluation of the appropriateness of admissions and duration of inpatient stay (Table 1).

Using Cohen's kappa (κ), we observed a high level of agreement in experts' reviews. Using Cohen's kappa (κ), we observed a high level of agreement in experts' reviews. Results for agreement on the appropriateness of admissions was 0.915 (95% CI (0.799 to 1.000) and duration of inpatient stay was 0.812 (95% CI 0.749 to 0.875).

The proposed method has very high operational characteristics: the area under the ROC curve only slightly varies for different medical specialties and averages to 0.948 for the appropriateness of admissions and to 0.900 for the duration of stay, which defines the potential to properly distinguish different patterns (discriminative capacity) of the method as excellent.

Core sample

Admissions at inpatient departments of secondary and tertiary care facilities were studied in two regions of Ukraine (central and western). The total of 12 healthcare facilities was included in the study, of which 9 facilities are secondary care hospitals, and 3 are tertiary care facilities.

The expert assessment covered a random sample of 800 medical records in four medical specialties: general medicine, neurology, surgery, and cardiology (Supplementary table 2). A similar approach was used to sample records for the core sample as in the pilot. All medical records for patients discharged during the month preceding the beginning of data collection (July 2016) were collected from all departments of the general medicine, surgery, neurology, and cardiology profile. The total number of sample medical records made up 8% to 12% of the total number of patients discharged from the selected departments during that month.

Experts, according to their specialization, reviewed medical records following the agreed guide for the analysis of the appropriateness of hospitalizations.

Results of data analysis for two regions of Ukraine

The findings of the expert review show that over one-third of cases – 38.1% (95% CI 33.9 to 43.5) in the central region, 38.7% (95% CI 34.8 to 41.5) in the western region and 37.6% (95% CI 32.8 to 42.3) in total were inappropriate hospital admissions (figure 2). Every seventh patient (13.3%; 95%CI 6.5% to 20.8%) admitted to an

	inpatient department of tertiary care facilities could have been more appropriately treated at secondary care facilities with adequate equipping and staffing.<br><br>In our sample, over half of the total bed days spent by patients in hospitals were classified as inappropriate or longer than necessary (57.4%). The volume of unnecessary bed-days in the western region was far greater (63.1%) than in the central region (51.7%, p<0.001), which was related to a higher rate of inappropriate admissions at secondary care facilities of patients to all specialized departments except the neurology profile (table 2).<br><br>The analysis of appropriateness of hospital admissions by specialties showed that the highest level of inappropriate admissions was recorded for patients treated in general medicine departments (53.7%); lower levels (p<0.001) were identified for all the other specialties: neurology – 34.2%; surgery – 27.3%; and cardiology – 23.3%.<br><br>The highest share of unnecessary stay was determined for patients of general medicine departments (64.6%), and the lowest share in cardiology (51.2%); for the neurology and surgery departments these shares were also high at 54.6% and 52.4% of total bed-days respectively.<br><br>The experts were asked to specify one of the proposed reasons for inappropriate hospitalization in each medical record reviewed. The following statistics describe the major reasons of inappropriate hospital admissions and unnecessary inpatient stays: to keep patients under care in the absence of other facilities with capacity to provide care to patients after discharge (21.0%; 95% CI 15.1 to 26.9), and to treat patients with overestimated severity of their condition (20.4%; 95% CI 14.6 to 26.3) (see Supplementary table 3 for all reasons). (page 8-11)
**Discussion**	***What does it mean?***
14. Summary	Like in the other countries, [8-9, 28-30] we have customized the European version of the AEP to adapt it to Ukrainian specifics. The scope of changes is marginal: we have amended some criteria, included relevant criteria and modified the wording. Such changes of the AEP are explained by the lower technological intensity of health care provision in Ukraine and peculiarities of service delivery in the country. The proposed Ukrainian version of Appropriateness Evaluation Protocol (AEP-UA) is a reliable and valid tool for assessment of the appropriateness of hospitalizations for Ukraine's

	hospitals, as confirmed by the high levels of Cohen's kappa coefficient (κ) for agreement on criteria of appropriateness and duration of hospitalizations (0.915 and 0.812 respectively).<br><br>Our study generates two key results regarding hospital care in Ukraine. First, there is a need for accurate assessment of the appropriateness of hospital admissions. To increase the objectivity of assessing the use of hospital resources, we suggest applying a unified method, customized for Ukraine, which gives reliable results for analyzing the appropriateness and duration of hospital admissions. Second, the reserves for optimization of the hospital sector in Ukraine are high, as evidenced by the volume of inappropriate hospitalizations in the two regions estimated using the proposed method. (page 11)
15. Interpretation	The results of our study point to a high volume of inappropriate hospitalizations and their unjustified duration in Ukraine. The number of unnecessary bed-days for appropriately hospitalized patients exceeded the number of bed-days from inappropriate admissions to the hospital by 27.8% (95% CI 24.4 to 31.1). This means that appropriately hospitalized patients often stay in the hospital longer than necessary. This share is especially large for patients hospitalized in general medicine departments – the numbers of unnecessary bed-days for appropriately admitted patients exceeds the number of bed-days for inappropriately admitted patients by 37.0% (95% CI 33.7 to 40.2).<br><br>We asked experts engaged in the evaluations to propose financial, economic, and organizational measures to prevent inappropriate hospitalizations or excessively long in-patient treatment (Supplementary table 4). Close to a quarter of respondents (24.3%; 95% CI 18.1 to 30.6) proposed to eliminate the normative regulation specifying the number of staff based on the number of beds in the facility. This principle inherited from the Soviet era, incentivized hospitals to maintain a large number of beds regardless of the actual needs for inpatient care. Although the issue had been included in the reform agenda for several decades, [15] it was only addressed in 2016 when the Ministry of Health abolished the existing normative regulation. This change, however, has not yet resulted in significant optimization of hospital capacities.<br><br>Hospitals in Ukraine do not differentiate by the intensity of interventions. In most hospitals, both acute and chronic patients, as well as those who need nursing and

social care, can stay in the same departments to receive medical care. Another proposed measure for improvement of hospital efficiency was to right-size the hospital network to align it with actual need in hospital care and categorize capacity of hospitals by intensity of care (14.9% of respondents, 95% CI 9.7 to 20.1), as well as the use of clear indications for admission to inpatient departments for facilities of different levels (32.6%, 95% CI 25.8 to 39.4). Such measures were proposed at the preliminary stage of the health reforms (2010-2014) [15] but not yet implemented.

The most popular measure proposed by experts was the introduction of alternatives to inpatient care: the replacement of inpatient services with outpatient services provided at the primary care level and the organization of treatment services without overnight stay (44.8% of respondents, 95% CI 37.5 – 52.0). This suggestion is in line with the current global trend of changes in the health system organization. [34, 35] Currently, the avoidance of hospitalizations of patients with ambulatory care-sensitive conditions (ACSCs) is included as a special policy direction on the agenda of many countries. [36-38] The rate of the appropriateness of hospitalizations can be seen as an indicator of the quality of primary health care. [39, 40] Larger capacity of primary care to treat ACSCs results in a significant reduction of the hospitalizations of patients with these conditions. [41] Day-care clinics and day-care centers, as well as socially appropriate and less expensive forms of medical care, are widely used in different countries as alternatives to hospital services. [3, 42, 43]

To increase the availability of alternative care for patients from remote areas, an organization of day care coupled with discharge to a boarding house facility has proven to be effective. [44]. In such facilities, a patient, after receiving the relevant service or intervention, is placed in a boarding house where a range of services is available, with a capacity for the provision of necessary care in the case of complications. This approach was favored by 33.7% of respondents (95% CI 26.8 to 40.6) [44]

Daycare facilities are also gaining popularity in Ukraine. In 2016, 31.6% more patients received medical care in daycare facilities compared with the total number of hospitalized patients, but the increase in the use of daycare facilities did not replace inpatient care. [15] According to large-scale international studies, the combination of incentives and special training of medical staff can help to improve the use of daycare instead of hospitalizations. [45, 46]

	An additional measure is the timely discharge of patients from hospitals (proposed by 19.9% of experts, 95% CI 14.1 to 25.7). The issue of appropriate discharge is not unique to Ukraine. European experts point out that there are obstacles to timely discharge in multiple countries. [1]<br><br>Interestingly, experts did not consider better coordination with social services as one of the factors to reduce inappropriate hospitalizations and duration of stay (only 5% respondents agreed with this statement, 95% CI 1,7 to 8,2), which can be explained by the low level of social service development and weak coordination between health and social sectors in the country. At the same time, strong evidence supports the need to create an interface between health and social care providers, which can help reduce levels of hospitalizations and their duration for patients, especially elderly [47, 48].<br><br>The current health reform prepared and launched by the Government and the Ministry of Health in 2018 focuses primarily on changing the payment mechanisms, which will influence changes in the hospital sector. At the same time, reforms also cover the primary health care and emergency services to ensure accessibility of services within the scope of limited available resources in the country. The results received and future use of the proposed method for the assessment of the appropriateness of hospitalizations can inform decisions in hospital restructuring. (page 12-14)
16. Limitations	There are several limitations that may limit the generalizability of the findings related to possible seasonal variations for hospital care statistics and selection of regions that may not be fully representative for the whole of Ukraine. To minimize and adjust for limitations, we compared the sample data from two regions with the data collected from an additional region during the pilot stage. We did not find any significant differences between the regional statistics: the level of inappropriate admissions in the pilot was 30.7% (95% CI 26.0 to 35.4), which is slightly lower than the total for the surveyed regions but could be explained by the inclusion of municipal hospitals in the pilot, which had lower levels of inappropriate hospitalizations in the main sample as well. We, therefore, assume that the facilities in our research are representative of inpatient facilities in Ukraine.<br><br>Among other limitations we report that we did not evaluate the actual need in hospital care in Ukraine, and the capacities of health care institutions to adequately address the needs. Because electronic medical records were not available, the quality of the assessment of paper-based medical records was compromised by the structure and

For peer review only - http://bmjopen.bmj.com/site/about/guidelines.xhtml

	legibility of hand-written text. We also suggest that the future use of the proposed method in practice may require additional training for evaluators; its sustainability will depend on uptake of the method at the national and at facility levels. (page 11-12)
17. Conclusions	Optimization of inpatient care by reducing inappropriate hospitalizations and excessive lengths of stay in hospitals, given the current economic situation, may become more than just an important direction in the development of the health care system to increase effectiveness. It can essentially help to sustain good progress in the improvement of health sector organization and health outcomes by adequately meeting the needs of the population in hospital care. Although the suggested transformations of the hospital sector may require additional funds for structural reorganization, training, and motivation of medical staff, these investments are likely to be offset by the large efficiency gains from hospital optimization. (page 14)
Other Information	
18. Funding	The study was supported by the World Bank (within Improving effectiveness in human development and social accountability Project financed by the Department for International Development). (page 15)
